# Synthesis and evaluation of squaramide and thiosquaramide inhibitors of the DNA repair enzyme SNM1A

**DOI:** 10.1016/j.bmc.2021.116369

**Published:** 2021-09-15

**Authors:** Mark Berney, William Doherty, Werner Theodor Jauslin, Manav T Manoj, Eva-Maria Dürr, Joanna Francelle McGouran

**Affiliations:** School of Chemistry & Trinity Biomedical Sciences Institute, Trinity College Dublin, The University of Dublin, Ireland

**Keywords:** SNM1A, Squaramide, Thiosquaramide, Interstrand crosslink repair, Zinc-binding group, Nucleoside, Nuclease inhibitor

## Abstract

SNM1A is a zinc-dependent nuclease involved in the removal of interstrand crosslink lesions from DNA. Inhibition of interstrand crosslink repair enzymes such as SNM1A is a promising strategy for improving the efficacy of crosslinking chemotherapy drugs. Initial studies have demonstrated the feasibility of developing SNM1A inhibitors, but the full potential of this enzyme as a drug target has yet to be explored. Herein, the synthesis of a family of squaramide- and thiosquaramide-bearing nucleoside derivatives and their evaluation as SNM1A inhibitors is reported. A gel electrophoresis assay was used to identify nucleoside derivatives bearing an *N*-hydroxysquaramide or squaric acid moiety at the 3′-position, and a thymidine derivative bearing a 5′-thiosquaramide, as candidate SNM1A inhibitors. Quantitative IC_50_ determination showed that a thymidine derivative bearing a 5′-thiosquaramide was the most potent inhibitor, followed by a thymidine derivative bearing a 3′-squaric acid. UV–Vis titrations were carried out to evaluate the binding of the (thio)squaramides to zinc ions, allowing the order of inhibitory potency to be rationalised. The membrane permeability of the active inhibitors was investigated, with several compounds showing promise for future *in vivo* applications.

## Graphical abstract

**Figure:**
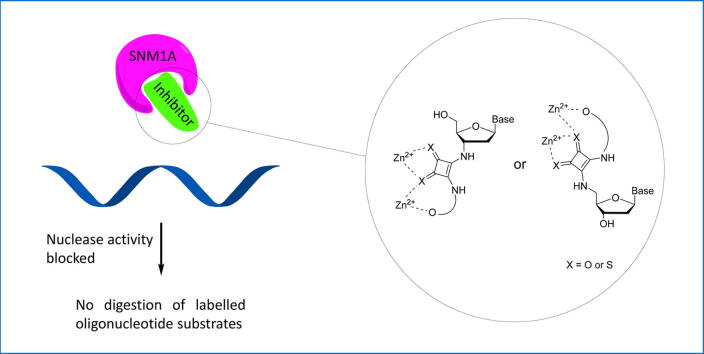

## 1 Introduction

Interstrand crosslinks (ICLs) are a highly cytotoxic form of DNA damage, as they prevent unwinding of the double helix and thereby inhibit DNA replication and transcription. Crosslinking agents are therefore widely used as chemotherapy drugs. However, certain cancers can develop resistance to crosslinking agents through overexpression of the enzymes involved in ICL repair pathways.^1^, ^2^, ^3^, ^4^ Developing inhibitors for these enzymes is a promising strategy for improving the efficacy of chemotherapy for drug-resistant cancers. Inhibitors for DNA repair enzymes such as *O*^6^-methylguanine methyltransferase and poly (ADP-ribose) polymerase, are approved for use in cancer treatment.^5^, ^6^, ^7^

The main ICL repair pathway in eukaryotic cells occurs after stalling of a replication fork at the site of an ICL. A key enzyme in this process is SNM1A, a 5′-3′ exonuclease which hydrolyses the phosphodiester backbone of the DNA past the site of the ICL.^8^, ^9^ Cells deficient in SNM1A show increased sensitivity to crosslinking agents such as SJG-136 and the anticancer drug mitomycin C.^10^ SNM1A is therefore a promising drug target, as inhibitors of this enzyme could be used to resensitise chemotherapy-resistant tumours.

A crystal structure of a truncated version of SNM1A has been reported, and shows a single zinc ion in the active site.^11^ However the crystal structure of the closely related enzyme SNM1B shows two zinc ions in the active site.^11^ It has been suggested that the active form of SNM1A contains a second zinc ion, or perhaps another metal cation, which is more loosely bound and therefore not observed in the crystal structure.^8^ These two zinc ions are predicted to activate a water molecule for nucleophilic attack on a phosphodiester in the DNA backbone, and stabilise a build-up of negative charge on the phosphodiester as it undergoes hydrolysis.

Previously reported inhibitors of SNM1A include the β-lactam antibiotics cephalosporins, which show strong inhibition *in vitro* but have low membrane permeability,^12^ and several hits identified from a high throughput screening of a library of bioactive compounds, which have an unclear mechanism of action.^13^ The development of substrate-mimic inhibitors for SNM1A has shown promising initial results. These compounds are based on a nucleoside scaffold, appended with a zinc-binding group (ZBG) to enhance binding to the active site. Inhibitors of this type which have demonstrated efficacy in *in vitro* testing include a thymidine derivative bearing a hydroxamic acid ZBG at the 5′-position^14^ and several malonate-bearing thymidine derivatives.^15^ There remains a need however for further development of substrate-mimic SNM1A inhibitors to fully investigate the potential of this under-studied enzyme as a drug target.

**Fig. 1:**
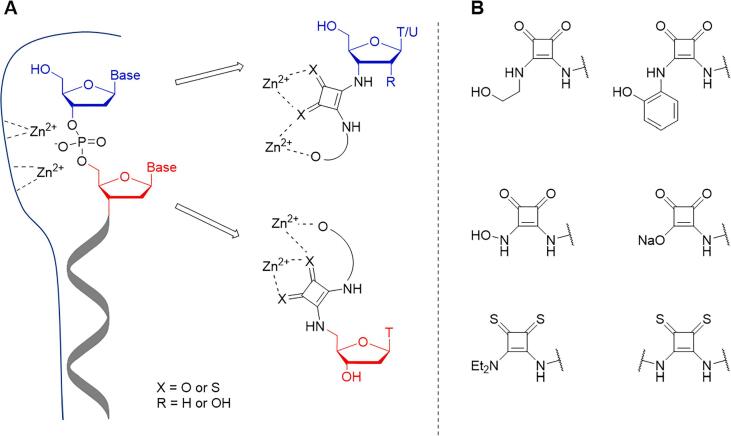
A) Design of squaramide-bearing substrate-mimic inhibitors of SNM1A based on a schematic view of substrate binding to the enzyme active site. B) Structures of squaramide and thiosquaramide motifs used as zinc-binding groups in SNM1A inhibitors.

In this work, we report the synthesis and testing of a series of substrate-mimic SNM1A inhibitors bearing squaramide or thiosquaramide ZBGs. Squaramides can chelate cations through their two carbonyl oxygen atoms,^16^ and *N*-hydroxysquaramides have shown promise as ZBGs in inhibitors for metalloproteases.^17^, ^18^ An oligonucleotide bearing a squaramide at the 5′-terminus has been shown to bind to SNM1A.^19^ However, the use of squaramides as metal chelators in biological applications has not been fully explored. In particular, we hypothesised that squaramides bearing substituents containing an additional oxygen atom could show improved binding to SNM1A due to chelation of the proposed second zinc ion in the SNM1A active site (Fig.  1A). Squaramides can also function as phosphate bioisosteres,^20^, ^21^ which is desirable for mimicking the natural DNA substrate of SNM1A. Thiosquaramides have more acidic NH protons than their oxo analogues,^22^ and up to now have principally been investigated for their anion binding properties^22^ and applications in organocatalysis.^23^, ^24^ However due to the affinity of sulfur for zinc, and the greater polarisation of thiosquaramides, we hypothesised that they could prove to be more potent ZBGs than their oxo analogues. For some squaramide-based metalloprotease inhibitors, replacement of one of the squaramide carbonyl groups with a thiocarbonyl moiety improved the inhibitory effect, however analogues containing two thiocarbonyls have not been explored to date.^17^

A range of nucleoside derivatives bearing differently substituted squaramides or thiosquaramides at the 3′- or 5′-position, as well as a dinucleoside linked through a thiosquaramide in place of a phosphodiester, have been synthesised (Fig.  1B). Several modified ribonucleosides have been prepared for comparison with deoxyribonucleoside inhibitors, as SNM1A can hydrolyse RNA as well as DNA *in vitro*.^8^, ^9^ These compounds have been tested as inhibitors of SNM1A in competition with oligonucleotide substrates, with several showing biological activity. The interaction of these compounds with zinc ions has been studied in UV–Vis titrations, and these results allow the relative potency observed in SNM1A inhibition assays to be rationalised. The membrane permeability of the candidate SNM1A inhibitors has been quantified using a parallel artificial membrane permeability assay (PAMPA).

## 2 Results and discussion

**Scheme 1:**
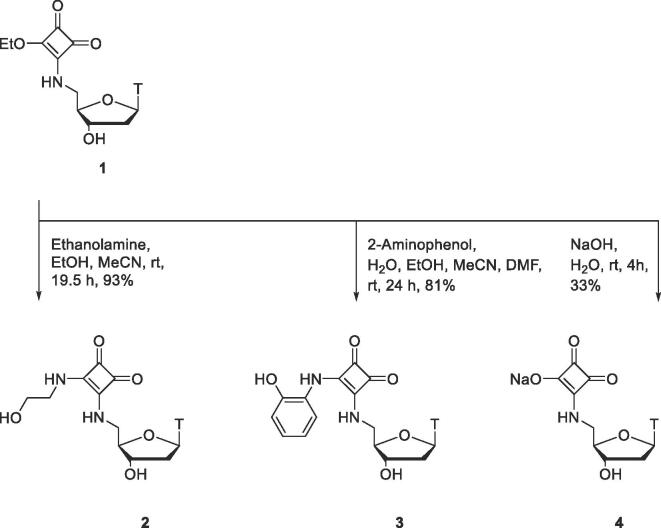
Synthesis of thymidine derivatives **2**–**4** bearing squaramides at the 5′-position.

A series of thymidine derivatives **2**–**4** bearing a squaramide group at the 5′-position were synthesised (Scheme 1). Squaramides **2** and **3** bear substituents containing an additional hydroxyl group, while compound **4** contains a squaric acid moiety, aimed at improving binding to active site zinc ions in SNM1A. Intermediate **1** was prepared as previously described,^14^, ^19^ and used for the generation of further functionalised squaramides **2**–**4**. Intermediate **1** was reacted with ethanolamine to prepare squaramide **2** in 93% yield. Separately, reaction of squaryl monoamide **1** with 2-aminophenol produced squaramide **3**, which is analogous to squaramide **2** in the positioning of the side-chain hydroxyl group, but has less conformational flexibility, which we reasoned could improve binding to zinc due to a lower entropic penalty. Intermediate **1** was also hydrolysed under basic conditions to provide squaric acid **4** in 33% yield.^25^

**Scheme 2:**
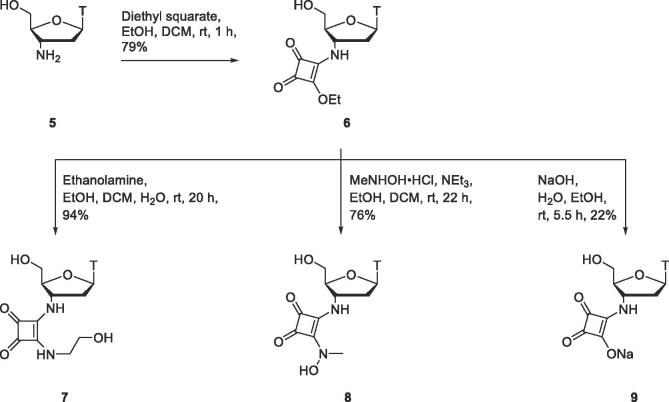
Synthesis of thymidine derivatives **6**–**9** bearing squaramides at the 3′-position.

Thymidine derivatives bearing squaramides at the 3′-position (**6**–**9**) were synthesised for comparison in biological assays with compounds **1**–**4** bearing the squaramide moiety at the 5′-position (Scheme 2). 3′-Amino-3′-deoxythymidine (**5**), prepared as previously described,^15^ was reacted with diethyl squarate to prepare squaryl monoamide **6**, a key intermediate for preparation of further functionalised squaramides **7**–**9**. Intermediate **6** was reacted with ethanolamine, and separately with *N*-methylhydroxylamine hydrochloride, to furnish squaramide **7** and *N*-hydroxysquaramide **8** respectively. Hydrolysis of squaryl monoamide **6** under basic conditions yielded squaric acid derivative **9** in a modest 22% yield.

*In vitro* studies have shown that SNM1A can hydrolyse RNA as well as DNA,^8^, ^9^ indicating that modified ribonucleosides could potentially function as SNM1A inhibitors. Furthermore, use of a ribonucleoside scaffold in place of a deoxyribonucleoside provides the potential for further functionalisation at the 2′-position for future optimisation of lead compounds. We therefore synthesised several uridine derivatives bearing squaramides at the 3′-position.

**Scheme 3:**
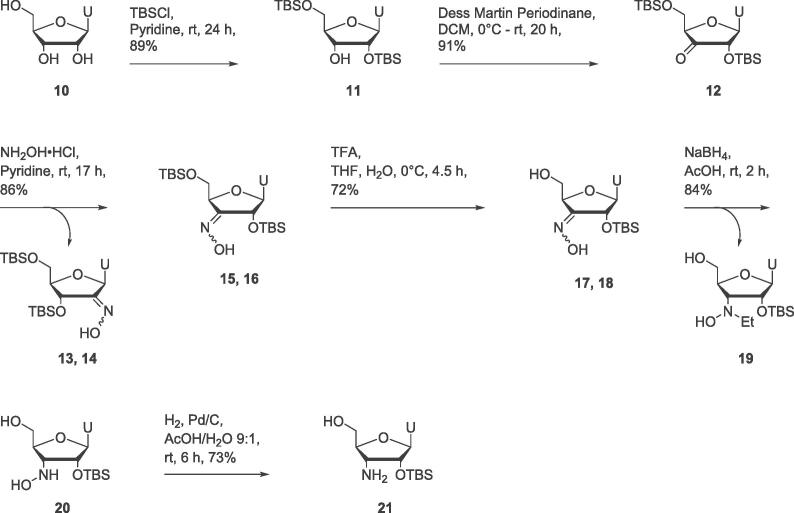
Synthesis of 3′-aminouridine derivative **21**.

To enable addition of a squaramide moiety at the 3′-position, uridine derivative **21** containing a 3′-amino group was prepared (Scheme 3). Reaction of uridine (**10**) with *tert*-butyldimethylsilyl chloride gave the 2′,5′-protected isomer **11** as the major product in 89% yield.^26^ 3′-Amino uridine derivative **21** was prepared from **11** through modification of a previously reported synthetic scheme.^27^, ^28^ Oxidation of protected uridine derivative **11** to ketone **12**, followed by condensation with hydroxylamine produced an oxime-bearing uridine derivative isolated as a mixture of E/Z isomers **15** and **16**. Formation of previously unidentified side products **13** and **14**, was also observed. These were presumably formed *via* silyl migration from the 2′- to the 3′-position in the enolate form of ketone **12**. Reducing the concentration of the reaction by approximately one third (from 0.88 M of hydroxylamine and 0.17 M of compound **12** to 0.56 M of hydroxylamine and 0.11 M of **12**) reduced the formation of side products **13** and **14** from 13% yield to a trace amount, and increased the yield of the desired products **15** and **16** from 67% to 86%. Attempts to reduce oxime isomers **15**/**16** were unproductive. However, after selective deprotection of the 5′-*O*-*tert*-butyldimethylsilyl group, E/Z oxime isomers **17** and **18** were successfully reduced using sodium triacetoxyborohydride formed *in situ* from sodium borohydride and acetic acid. Formation of trace amounts of alkylated side product **19** was observed, likely due to acetylation of hydroxylamine product **20** by boron triacetate followed by reduction of the resulting amide. Hydroxylamine product **20** was formed stereoselectively, which can be attributed to coordination of the 5′-hydroxyl group of oximes **17**/**18** to boron.^29^ Hydrogenation of hydroxylamine **20** afforded amine **21**, the key intermediate allowing further functionalisation with squaramide moieties at the 3′-position of uridine derivatives.

**Scheme 4:**
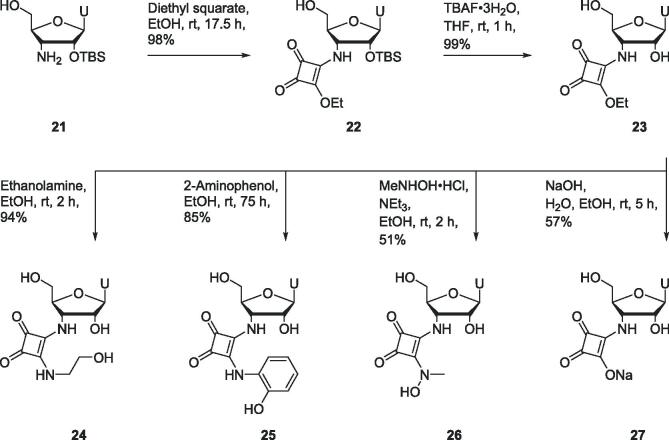
Synthesis of uridine derivatives **23**–**27** bearing squaramides at the 3′-position.

Uridine derivative **22** containing a squaramide at the 3′-position was prepared in 98% yield by reaction of amine **21** with diethyl squarate (Scheme 4). The silyl protecting group of squaramide **22** was removed with TBAF to provide the product **23** in 99% yield. Squaryl monoamide **23** acted as a key intermediate in the preparation of squaramides **24**–**27**. Squaryl monoamide **23** was treated with ethanolamine, and separately with 2-aminophenol to produce squaramides **24** and **25** respectively. *N*-Hydroxysquaramide **26** was prepared in 51% yield by reaction of squaryl monoamide **23** with *N*-methylhydroxylamine hydrochloride. Hydrolysis of squaryl monoamide **23** under basic conditions provided squaric acid derivative **27** in 57% yield.

**Scheme 5:**
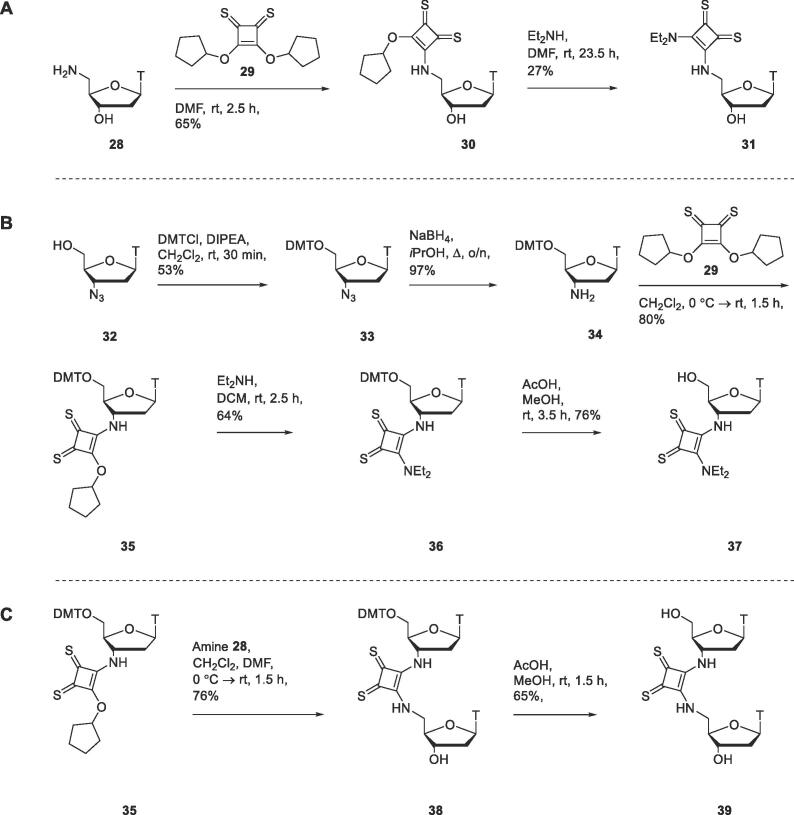
A) Synthesis of a thymidine derivative **31** bearing a 5′-thiosquaramide. B) Synthesis of a thymidine derivative **37** bearing a 3′-thiosquaramide. C) Synthesis of a dinucleoside **39** containing a bridging thiosquaramide in place of a phosphodiester linkage.

A series of thiosquaramide-bearing nucleosides **31**, **37** and **39** were prepared to test whether these sulfur-containing functional groups would prove to be more effective inhibitors of the zinc metalloenzyme SNM1A than their oxo analogues. 5′-Aminothymidine **28**, prepared as previously described,^19^ was reacted with dicyclopentyl dithiosquarate^30^ **29** to prepare thiosquaryl monoamide **30** in 65% yield (Scheme 5A). Thiosquaryl monoamide **30** was reacted with diethylamine to provide thiosquaramide **31** in 27% yield.

To prepare a thymidine derivative bearing a thiosquaramide at the 3′-position, azidothymidine (**32**) was reacted with 4,4′-dimethoxytrityl chloride to generate protected thymidine derivative **33** in 53% yield (Scheme 5B).^31^ Azide **33** was reduced to amine **34**,^32^ which was further reacted with dicyclopentyl dithiosquarate **29** to provide thiosquaryl monoamide **35**. Initially a low yield of **35** was obtained, however when the reaction was carried out in the dark, compound **35** was prepared in 80% yield. It was observed that a number of the other thiosquaramides appeared to decompose after exposure to light for several hours. This is consistent with the reported photoactivity of thiocarbonyls, which can be excited by visible light.^33^, ^34^ Following this observation care was taken to shield thiosquaramides from light during subsequent reactions. Further reaction of thiosquaryl monoamide **35** with diethylamine provided thiosquaryl diamide **36** in 64% yield. Deprotection of thiosquaramide **36** furnished the target compound **37** in 76% yield.

We hypothesised that binding to SNM1A could be improved by using a dinucleoside inhibitor rather than a mononucleoside, more closely mimicking the natural DNA substrate and increasing the number of possible stabilising non-covalent interactions with the enzyme. This strategy was previously applied in the development of SNM1A inhibitors bearing malonate/malonamide ZBGs; dinucleosides linked through a malonamide were slightly more effective inhibitors than their mononucleoside analogues bearing a malonamide at the 3′-position.^15^ A dinucleoside **39** containing a thiosquaramide linkage was therefore prepared (Scheme 5C). Thiosquaryl monoamide **35** was reacted with 5′-amine **28** to produce thiosquaramide **38**. The DMT protecting group was removed under acidic conditions to provide the target dinucleoside **39** in 65% yield.

The ability of the squaramide- and thiosquaramide-bearing nucleoside derivatives to inhibit SNM1A was first evaluated using a gel electrophoresis-based assay, as previously described.^12^, ^19^ The nucleoside derivatives were incubated at a concentration of 1 mM in solution with SNM1A at 37 °C for 5 min, before addition of a 21mer oligonucleotide substrate, labelled with a fluorophore at the 3′-terminus, and further incubation for 1 h. The extent of digestion of the oligonucleotide substrate was examined by polyacrylamide gel electrophoresis (PAGE). SNM1A does not show significant nuclease activity on DNA strands of 8 nucleotides or less,^9^ and digestion of the oligonucleotide substrate to this length therefore indicates full activity of the enzyme. The presence of longer oligonucleotides in the PAGE readout is indicative of effective inhibition of SNM1A. Thymidine was included in this assay as a control to verify that inhibitory effects result from the squaramide or thiosquaramide modifications.

**Fig. 2:**
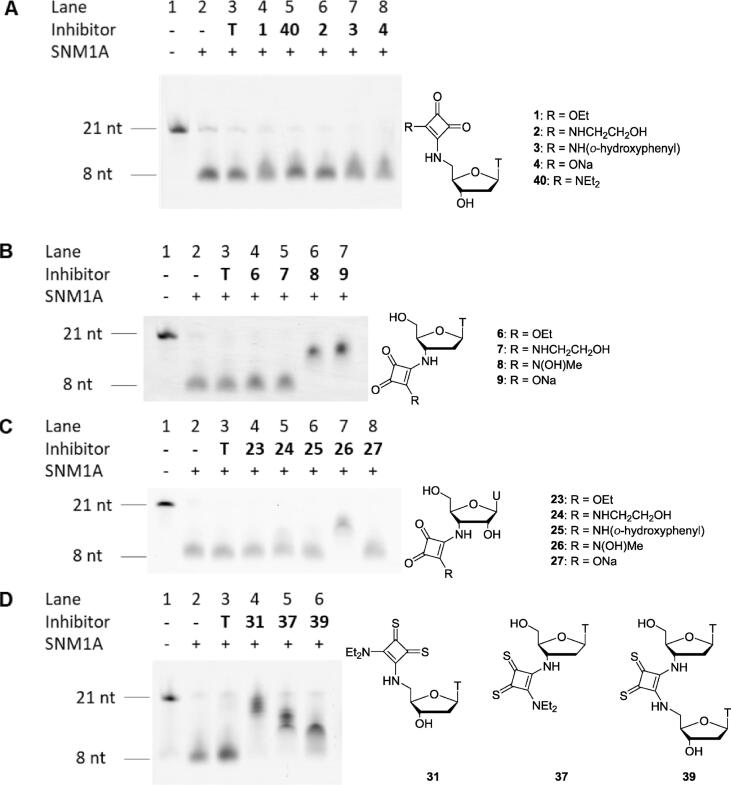
Biological evaluation of SNM1A inhibitors through visualisation of the extent of digestion of a 21mer fluorescent oligonucleotide substrate by denaturing PAGE. SNM1A (2.5 nM) was pre-incubated with the modified nucleosides (1 mM) for 5 min before the oligonucleotide substrate (80 nM) was added, and then incubated for a further 1 h. nt = nucleotides. A) Evaluation of thymidine derivatives **2**–**4** bearing a squaramide at the 5′-position. B) Evaluation of thymidine derivatives **6**–**9** bearing a squaramide at the 3′-position. C) Evaluation of uridine derivatives **23**–**27** bearing a squaramide at the 3′-position. D) Evaluation of thiosquaramides **31**, **37** and **39**.

The results of the inhibitor screen show that thymidine derivatives **1**–**4** bearing squaramide groups at the 5′-positon do not inhibit SNM1A to any significant extent (Fig.  2A). Compound **40**, a squaramide without a substituent that could contribute to chelation of zinc, was prepared as previously described^14^ and tested as an additional control in this experiment, exhibiting no inhibition of SNM1A. This is consistent with previous results that showed compounds **1** and **40** do not inhibit SNM1A.^14^ Squaramides **2**–**4** did not show improved binding to SNM1A despite their additional functionalisation. Testing of thymidine derivatives **6**–**9** bearing squaramides at the 3′-position however revealed that compounds **8** and **9** inhibit SNM1A (Fig.  2B), as does uridine derivative **26** (Fig.  2C), an analogue of thymidine derivative **8**. Squaramide-bearing nucleoside derivatives thus appear to inhibit SNM1A more effectively when the squaramide moiety is placed at the 3′-position rather than the 5′-position of the inhibitor. Compound **9** bearing a 3′-squaric acid inhibits SNM1A, while compound **4** bearing a 5′-squaric acid does not. The squaric acid and *N*-hydroxysquaramide moieties appeared to be more effective than the other squaramides tested.

Thiosquaramides **31**, **37** and **39** were also screened in the gel-electrophoresis based assay. Contrary to the trend observed for squaramides, thymidine derivative **31** bearing a thiosquaramide at the 5′-position showed greater inhibition of SNM1A than thymidine derivative **37** bearing a thiosquaramide at the 3′-position (Fig.  2D). This may indicate a different binding mode to SNM1A due to the larger steric bulk of thiosquaramides relative to their oxo analogues. Thiosquaramide-linked dinucleoside **39** inhibited SNM1A less effectively than either of the thiosquaramide-bearing mononucleosides **31** and **37**. This was likely due to the conformational rigidity of the thiosquaramide linkage of dinucleoside **39**, restricting the molecule to a less favourable binding conformation, different to that of the natural DNA substrate. Inclusion of a squaramide internucleotide linkage in oligonucleotides has previously been found to cause distortion of normal duplex structure.^21^

**Fig. 3:**
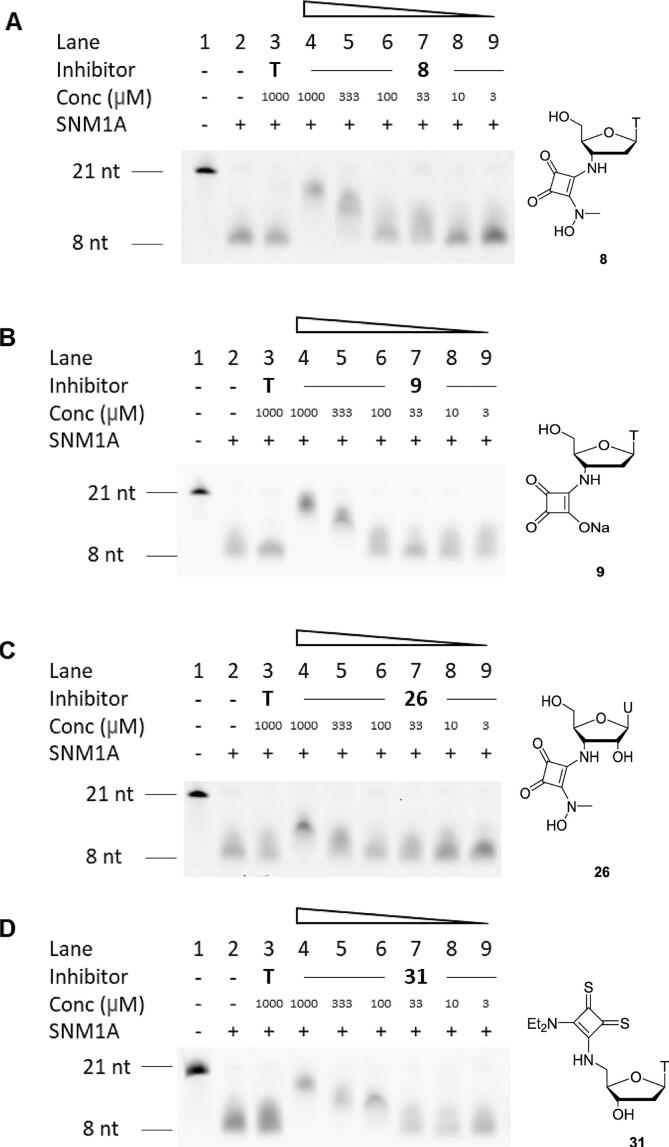
Evaluation of SNM1A inhibitors at a range of concentrations from 1 mM to 3 µM through visualisation of the extent of digestion of a fluorescent oligonucleotide substrate by denaturing PAGE. SNM1A (2.5 nM) was pre-incubated with the modified nucleosides for 5 min before the 21-mer oligonucleotide substrate (125 nM) was added and a further 60 min incubation. nt = nucleotides. A) Evaluation of thymidine 3′-*N*-hydroxysquaramide **8**. B) Evaluation of thymidine 3′-squaric acid **9**. C) Evaluation of uridine 3′-*N*-hydroxysquaramide **26**. D) Evaluation of thymidine 5′-thiosquaramide **31**.

Following their identification as candidate SNM1A inhibitors, compounds **8**, **9**, **26**, and **31** were tested individually at concentrations ranging from 1 mM to 3 µM (Fig. 3). This showed that thymidine *N*-hydroxysquaramide **8** is a slightly stronger inhibitor, showing inhibition at 333 µM, (Fig. 3A) than its uridine analogue **26**, which only shows significant inhibition at 1 mM (Fig. 3C). When tested simultaneously in the same assay (Fig. 2B), thymidine derivative **9** bearing a squaric acid moiety appears to show slightly more inhibition of SNM1A at 1 mM than thymidine derivative **8** bearing an *N*-hydroxysquaramide, although it is difficult to confirm this difference in inhibitory effect based on individual testing of compounds **8** and **9** (Fig. 3A and 3B) as both show a degree of inhibition at 333 µM. *N*-Hydroxysquaramides such as **8** and **26** can chelate to zinc through formation of a 6-membered ring, while chelation of squaric acid **9** to zinc through formation of a 5-membered ring is considered less favourable due to the larger bite angle.^35^ However, these results indicate that increased electrostatic interaction with zinc due to the negative charge of compound **9** in its deprotonated form may be a more important factor in determining inhibitory potency. Thymidine derivative **31** bearing a thiosquaramide at the 5′-position appears to be the most potent of all the SNM1A inhibitors tested, showing inhibition at a concentration of 100 µM (Fig. 3D).

**Fig. 4:**
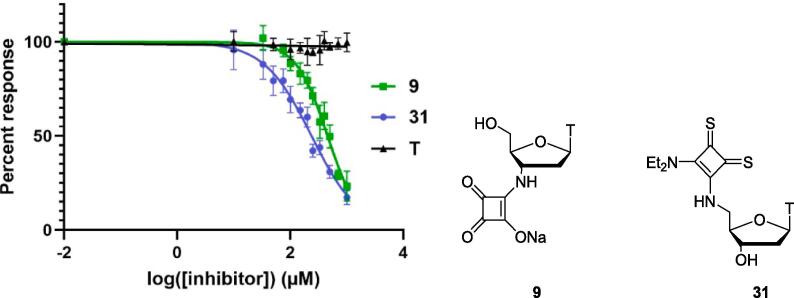
IC_50_ determination of modified nucleosides **9** and **31** in a real-time fluorescence assay. Thymidine (**T**) is included as a control. Error bars were generated from 6 independent repeats.

Following identification of the most promising compounds **9** and **31**, a real-time fluorescence assay was carried out,^12^, ^14^ to determine the IC_50_ values for inhibition of SNM1A (Fig. 4). Unmodified thymidine was also tested in this assay as a control, showing no inhibition of SNM1A. Thymidine derivative **31** bearing a 5′-thiosquaramide again proved to be the most potent inhibitor, with an IC_50_ of 238 µM. The IC_50_ of thymidine derivative **9** with a squaric acid moiety at the 3′-position was found to be 456 µM. Thymidine derivative **8** bearing a 3′-*N*-hydroxysquaramide and thymidine derivative **37** bearing a 3′-thiosquaramide were also tested in this assay for comparison with squaric acid **9** and 5′-thiosquaramide **31**, to confirm the trends in inhibitory potency seen in the gel electrophoresis assays. Some inhibition of SNM1A by *N*-hydroxysquaramide **8**, and to a lesser extent by thiosquaramide **37** was observed (Fig. S1), however the IC_50_ values for these compounds could not be accurately determined as they appeared to be above the concentration range tested. These results confirm the trend observed in the gel electrophoresis assays; 5′-thiosquaramide **31** is a more potent inhibitor than 3′-thiosquaramide **37**, and thymidine derivative **9** with a squaric acid moiety at the 3′-position is a more potent inhibitor than thymidine derivative **8** bearing an *N*-hydroxysquaramide at the 3′-position. 5′-Thiosquaramide **31** is the most effective of all the inhibitors tested.

To investigate whether SNM1A inhibition occurs through coordination of active site zinc ions by the squaramide moieties, and to rationalise the relative inhibitory potency of compounds **8**, **9** and **31**, solutions of these compounds in MeCN were used in UV–Vis titrations with Zn(ClO_4_)_2_ (Fig. S2). The titration data were analysed by global non-linear regression using the ReactLab Equilibria software to elucidate binding modes. The experimental binding constants obtained are summarised in Table 1. For thymidine derivative **8** bearing a 3′-*N*-hydroxysquaramide, the best fit to the data was obtained using a two-species model in which **8** binds to a metal ion in both a 1:1 and 1:2 ligand-metal ratio. The equilibrium constant calculated for formation of the 1:1 species was 3.0 × 10^4^ M^−1^, and the equilibrium constant for formation of the 1:2 species was significantly lower at 6.6 × 10^1^ M^−1^. Thymidine derivative **9** bearing a squaric acid at the 3′-position exhibited stronger binding to zinc, with an equilibrium constant of 4.0 × 10^4^ M^−1^ for 1:1 ligand-metal binding. The data indicated squaric acid **9** could participate 2:1 ligand-metal binding in MeCN solution with an equilibrium constant of 3.2 × 10^6^ M^−1^, although a 2:1 ligand-metal binding mode is unlikely to be accommodated within the SNM1A active site. The strongest zinc binding was observed when Zn(ClO_4_)_2_ was titrated against thymidine derivative **31** bearing a thiosquaramide at the 5′-position. The data from this titration were best fit by a three-species model in which ligand **31** binds to metal ions in a 1:1, 2:1, and 1:2 ratio. The equilibrium constant calculated for 1:1 ligand-metal binding was 6.8 × 10^5^ M^−1^. For 2:1 ligand-metal binding the calculated equilibrium constant was 1.2 × 10^6^ M^−1^, and 1:2 ligand-metal binding was less significant with an equilibrium constant of 1.7 × 10^2^ M^−1^. Although these titrations should be considered as qualitative, given that MeCN solution is not a close representation of the SNM1A active site environment, the order of 1:1 binding strength to zinc ions observed, thiosquaramide **31** > squaric acid **9** > *N*-hydroxysquaramide **8**, is the same as the trend in the potency of SNM1A inhibition observed in both the gel electrophoresis and real-time fluorescence assays. This supports the hypothesis that the compounds inhibit SNM1A through binding to the active site zinc ion(s).

**Table 1:** Equilibrium constants for binding of squaramides to Zn^2+^ calculated from UV–Vis titration data.

	Ligand-metal binding constants		
Ligand	K_1:1_	K_1:2_	K_2:1_
**8**	3.0 × 10^4^ M^−1^	6.6 × 10^1^ M^−1^	–
**9**	4.0 × 10^4^ M^−1^	–	3.2 × 10^6^ M^−1^
**31**	6.8 × 10^5^ M^−1^	1.7 × 10^2^ M^−1^	1.2 × 10^6^ M^−1^

To evaluate the potential of the SNM1A inhibitors for future use in *in vivo* applications, a parallel artificial membrane permeability assay (PAMPA)^36^ was carried out to examine the membrane permeability of the biologically active compounds **8**, **9**, **26**, **31**, **37** and **39** (Fig. S3). Thymidine derivative **8** bearing 3′-*N*-hydroxysquaramide, and thymidine derivative **9** bearing a squaric acid at the 3′-position were found to be membrane permeable, with logP_e_ values of −4.6 ± 0.4 and −4.1 ± 0.6 respectively. Unlike its thymidine analogue **8**, uridine derivative **26** bearing a 3′-*N*-hydroxysquaramide was found to be impermeable, with a logP_e_ of −7.0 ± 0.1. Of the thiosquaramides, 5′-thiosquaramide **31** was found to have a low degree of membrane permeability, with a logP_e_ value of −6.5 ± 0.6. 3′-Thiosquaramide **37** and bridging thiosquaramide **39** showed no membrane permeability, with logP_e_ values of −7.01 ± 0.04 and −7.084 ± 0.005 respectively. The three most potent inhibitors, **8**, **9**, and **31**, all show some degree of membrane permeability, demonstrating their potential for future biological applications.

## 3 Conclusion

In summary, we have synthesised a range of nucleoside derivatives bearing squaramide and thiosquaramide modifications. These compounds were tested as inhibitors of SNM1A in gel electrophoresis and real-time fluorescence assays. Their physical properties were also examined in a PAMPA assay to determine membrane permeability, and in UV–Vis titrations to investigate their interaction with zinc ions. Although compounds bearing a squaramide group at the 5′-position proved ineffective, nucleoside derivatives **8**, **9**, and **26** bearing a squaramide moiety at the 3′-position demonstrated inhibition of SNM1A. Gel electrophoresis assays showed that thymidine derivative **8** bearing an *N*-hydroxysquaramide at the 3′-position is a more effective inhibitor of SNM1A than its uridine analogue **26**. This is consistent with the enzyme’s preference for DNA substrates over RNA. Compound **9** bearing a squaric acid moiety at the 3′-position was a more effective inhibitor than either of the *N*-hydroxysquaramides **8** and **26**. UV–Vis titrations showed that this could be attributed to a stronger interaction of squaric acid **9** with zinc ions compared to *N*-hydroxysquaramide **8**. Interestingly, thymidine derivative **31** bearing a thiosquaramide at the 5′-position proved to be a more effective inhibitor of SNM1A than compound **37** in which the thiosquaramide is placed at the 3′-position, contrary to the trend observed for the oxo squaramides. We hypothesise that this is because when the more sterically bulky thiosquaramide moiety is placed at the 3′-position, it causes the nucleobase and the deoxyribose ring to be displaced from their ideal binding position in the SNM1A active site, resulting in steric clashes with amino acid residues. Although the testing of the oxo squaramides suggests that the ideal placement of a less bulky ZBG appears to be at the 3′-position, 5′-thiosquaramide **31** is nonetheless the most potent of any of the inhibitors tested, with an IC_50_ value of 238 µM. UV–Vis titrations with Zn(ClO_4_)_2_ indicate that this is due to a much stronger interaction of the thiosquaramide with zinc ions compared to that observed for oxo squaramides. Finally, an assay was carried out to determine the membrane permeability of the SNM1A inhibitors to evaluate their potential for future *in vivo* applications. Squaramides **8** and **9** showed a high degree of membrane permeability, while the most potent inhibitor thiosquaramide **31** was permeable to a lesser degree. Taken together, the results of this study demonstrate the potential of squaramide and thiosquaramide moieties for targeting zinc-dependent enzymes in biological applications and provide insights useful for the further development of substrate-mimic inhibitors of SNM1A.

## Funding

This work was supported by the Irish Research Council (GOIPG/2017/1453) and the Wellcome Trust Trinity College Dublin (TCD) institutional strategic support fund.

## Declaration of Competing Interest

The authors declare that they have no known competing financial interests or personal relationships that could have appeared to influence the work reported in this paper.
