# Supplementary material for: Synthesis and evaluation of squaramide and thiosquaramide inhibitors of the DNA repair enzyme SNM1A
Source: Bioorg Med Chem. 2021 Sep 15;46:116369. doi: 10.1016/j.bmc.2021.116369 (PMC8607331; doi:10.1016/j.bmc.2021.116369)
Supplement: Supplementary data 1 [file mmc1.docx]

**Supplementary Information**

Synthesis and Evaluation of Squaramide and Thiosquaramide Inhibitors of the DNA Repair Enzyme SNM1A

Mark Berney, William Doherty, Werner T. Jauslin, Manav T Manoj, Eva-Maria Dürr and Joanna F. McGouran.

[Synthetic methods 1](#_Toc73360310)

[Thymidine 5’-squaramides 1](#_Toc73360311)

[Thymidine 3’-squaramides 4](#_Toc73360312)

[Uridine 3’-squaramides 8](#_Toc73360313)

[Thiosquaramides 24](#_Toc73360314)

[Gel electrophoresis assay 35](#_Toc73360315)

[Real-time fluorescence assay 35](#_Toc73360316)

[UV-vis titrations 37](#_Toc73360317)

[Parallel artificial membrane permeability assay (PAMPA) 39](#_Toc73360318)

[^1^H and ^13^C NMR Spectra of Novel Compounds 41](#_Toc73360319)

[References 63](#_Toc73360320)

# Synthetic methods

## Thymidine 5’-squaramides

**5'-*N*-(2-(2-Hydroxyethyl)amino-3,4-dioxocyclobut-1-en-1-yl)amino-5'-deoxythymidine (2)**

5'-*N*-(2-Ethoxy-3,4-dioxocyclobut-1-en-1-yl)amino-5'-deoxythymidine (**1**) (36 mg, 99 μmol) was suspended in EtOH (1 mL). Ethanolamine (9 μL, 149 μmol) was added and the reaction mixture was stirred at room temperature for 2 hours. TLC analysis (EtOAc-MeOH, 85:15) after this time showed very little consumption of starting material (R_f_ = 0.6) and minimal formation of product (R_f_ = 0.3). MeCN (1 mL) was added and the reaction mixture was stirred at room temperature for a further 18 hours. The solvent was removed under reduced pressure and the residue was purified by flash chromatography (EtOAc‑MeOH, 88:12 then 85:15) to provide the product **2** as a white crystalline solid (35 mg, 93%); mp 197-202 °C (decomp).

ν_max_/cm^-1^ (neat) 3229, 2926, 1802, 1659, 1586, 1535, 1474, 1425, 1349, 1268, 1050, 959.

^1^H NMR (600 MHz, DMSO-d_6_): *δ* = 1.78 (s, 3H, CH_3_^T^), 2.05-2.10 (m, 1H, H-2’a), 2.14-2.20 (m, 1H, H‑2’b), 3.50 (br s, 2H, CH_2_), 3.55 (br s, 2H, CH_2_), 3.69 (br s, 1H, H-5’a), 3.79-3.83 (m, 1H, H-4’), 3.89 (br s, 1H, H-5’b), 4.19 (br s, 1H, H-3’), 4.92 (br s, 1H, OH), 5.42 (br s, 1H, OH), 6.19 (app t, J = 6.9 Hz, H‑1’), 7.44 (s, 1H, H-6), 7.83 (br s, 2H, 2 x NH^Sq^), 10.97 (br s, 1H, NH^T^) ppm.

^13^C NMR (151 MHz, DMSO-d_6_): *δ* = 12.0 (CH_3_^T^), 38.4 (C-2’), 45.3 (C-5’), 45.8 (CH_2_), 60.8 (CH_2_), 70.6 (C‑3’), 83.6 (C-1’), 85.2 (C-4’), 109.9 (C-5), 135.9 (C-6), 150.5 (C-2), 163.7 (C-4), 167.8 (C-Sq), 168.1 (C‑Sq), 182.4 (C-Sq), 182.6 (C-Sq) ppm.

HRMS (APCI^-^): *m/z* calc. 379.1259 [M-H]^-^, 379.1257

**5'-*N*-(2-(2-Hydroxyphenyl)amino-3,4-dioxocyclobut-1-en-1-yl)amino-5'-deoxythymidine (3)**

5'-*N*-(2-Ethoxy-3,4-dioxocyclobut-1-en-1-yl)amino-5'-deoxythymidine (**1**) (32 mg, 88 μmol) was suspended in water (1 mL) and EtOH (1 mL). 2-Aminophenol (51 mg, 467 μmol) was added and the reaction mixture was stirred at room temperature for 24 hours. After this time MeCN (1 mL) was added and the reaction mixture was stirred at room temperature for a further 3 hours. After this time DMF (0.8 mL) was added to fully dissolve the reaction mixture. The reaction mixture was stirred at room temperature for a further 21 hours. TLC analysis (CH_2_Cl_2_-MeOH, 90:10) after this time showed consumption of starting material (R_f_ = 0.4) and formation of product (R_f_ = 0.3). The solvent was removed under reduced pressure. The residue was co-evaporated with toluene and then purified by flash chromatography (CH_2_Cl_2_‑MeOH, 95:5 ‑ 90:10) to obtain the product **3** as a brown crystalline solid (31 mg, 81%); mp 201-207 °C.

ν_max_/cm^-1^ (neat) 3242, 3066, 2975, 1795, 1661, 1586, 1531, 1455, 1366, 1269, 1210, 1086, 1022, 840, 747.

^1^H NMR (600 MHz, DMSO-d_6_): *δ* = 1.76 (s, 3H, CH_3_^T^), 2.11 (ddd, J_2’a,2’b_ = 13.6 Hz, J_1’,2’a_ = 6.6 Hz, J = 4.0 Hz, 1H, H‑2’a), 2.22 (app dt, J_2’a,2’b_ = 13.6 Hz, J_1’,2’b_ = 6.6 Hz, 1H, H-2’b), 3.71-3.79 (m, 1H, H-5’a), 3.84 (app dt, J = 7.6 Hz, J = 3.7 Hz, 1H, H-4’), 4.03 (ddd, J_5’a,5’b_ = 10.3 Hz, J_5’b,NH-5’_ = 6.1 Hz, J_4’,5’b_ = 3.7 Hz, 1H, H‑5’b), 4.20-4.24 (m, 1H, H-3’), 5.46 (br s, 1H, OH-3’), 6.23 (app t, J = 6.6 Hz, 1H, H-1’), 6.76 (app dt, J = 4.1 Hz, J = 8.1 1H, H^Ar4^), 6.86 (app d, J = 4.1 Hz, 2H, H^Ar3^, H^Ar5^), 7.43 (s, 1H, H-6), 7.76 (d, J_Ar2,Ar4_ = 8.1 Hz, 1H, H^Ar2^), 8.45 (t, J_5’,NH-5’_ = 6.1 Hz, 1H, NH-5’), 9.31 (s, 1H, NH^Sq^), 10.18 (br s, 1H, OH-Ar), 11.32 (br s, 1H, NH^T^) ppm.

^13^C NMR (151 MHz, DMSO-d_6_): *δ* = 12.0 (CH_3_^T^), 38.4 (C-2’), 45.6 (C-5’), 70.6 (C-3’), 83.5 (C-1’), 85.0 (C‑4’), 110.0 (C-5), 115.0 (C-Ar5), 119.3 (C-Ar4), 120.0 (C-Ar2), 123.6 (C-Ar3), 127.0 (C-Ar1), 136.0 (C‑6), 146.7 (C-Ar6), 150.5 (C-2), 163.65 (C-4), 163.72 (C-Sq2), 169.2 (C-Sq1), 180.3 (C-Sq3), 184.1 (C‑Sq4) ppm.

HRMS (APCI^-^): *m/z* calc. 427.1259 [M-H]^-^, 427.1258

**5'-*N*-(2-Hydroxy-3,4-dioxocyclobut-1-en-1-yl)amino-5'-deoxythymidine**^1^ **(4)**

5'-*N*-(2-Ethoxy-3,4-dioxocyclobut-1-en-1-yl)amino-5'-deoxythymidine (**1**) (40 mg, 109 μmol) was suspended in water (1.4 mL). NaOH (10 mg, 260 μmol) was added and the reaction mixture was stirred at room temperature for 4 hours. TLC analysis (EtOAc-MeOH, 85:15) after this time showed complete consumption of starting material (R_f_ = 0.6) and formation of product (R_f_ = 0.1). The reaction mixture was eluted through Dialon WT01S ion-exchange resin (H form) and then eluted through Dialon WT01S ion‑exchange resin (Na form). The solvent was removed under reduced pressure and the residue was purified by flash chromatography (water‑*i*PrOH-EtOAc, 1:5:4) to provide the product **4** as a white crystalline solid (13 mg, 33%); mp 218-222 °C (decomp).

ν_max_/cm^-1^ (neat) 3299, 2908, 1792, 1637, 1618, 1515, 1414, 1367, 1273, 1237, 1133, 1052, 1035, 979, 931, 795, 751.

^1^H NMR (400 MHz, D_2_O): *δ* = 1.84 (s, 3H, CH_3_^T^), 2.38-2.43 (m, 2H, H-2’a, H-2’b), 3.80 (dd, J_5’a,5’b_ = 14.6 Hz, J_4’,5’a_ = 4.7 Hz, 1H, H‑5’a), 3.98 (dd, J_5’a,5’b_ = 14.6 Hz, J_4’,5’b_ = 4.1 Hz, 1H, H-5’b), 4.09 (dd, J_4’,5’a_ = 4.7 Hz, J_4’,5’b_ = 4.1 Hz, 1H, H-4’), 4.48-4.56 (m, 1H, H-3’), 6.25 (app t, J = 6.6 Hz, 1H, H‑1’), 7.35 (s, 1H, H-6) ppm.

^13^C NMR (100 MHz, D_2_O): *δ* = 11.5 (CH_3_^T^), 37.8 (C-2’), 44.3 (C-5’), 70.4 (C-3’), 84.7 (C-1’), 84.8 (C-4’), 111.6 (C-5), 136.9 (C-6), 151.7 (C-2), 166.3 (C-4), 181.8 (C-Sq1), 188.1 (C-Sq3), 195.1 (C-Sq2, C-Sq4) ppm.

HRMS (ESI^+^): *m/z* calc. 382.0622 [M+Na]^+^, found: 382.0625

## Thymidine 3’-squaramides

**3'-*N*-(2-Ethoxy-3,4-dioxocyclobut-1-en-1-yl)amino-3'-deoxythymidine (6)**

3’-Amino-3’-deoxythymidine (**5**) (502 mg, 2.08 mmol) was suspended in a mixture of EtOH (12 mL) and CH_2_Cl_2_ (6 mL). Diethyl squarate (0.46 mL, 3.11 mmol) was added, and the reaction mixture was stirred at room temperature. After 10 minutes the suspension dissolved. TLC analysis (CH_2_Cl_2_-EtOH, 92:8) after 45 minutes showed complete consumption of starting material (R_f_ = 0.0) and formation of product (R_f_ = 0.8). The solvent was removed under reduced pressure, and the residue was purified by flash chromatography (CH_2_Cl_2_-MeOH, 96:4). The product **6** was obtained as a white powder (602 mg, 79%); mp 172-176 °C.

Note: compound **6** exhibits rotamers in NMR spectroscopy.

ν_max_/cm^-1^ (neat) 3197, 3040, 1805, 1686, 1596, 1421, 1269, 993, 736, 695, 598.

^1^H NMR (400 MHz, DMSO-d_6_): δ = 1.37 (br s, 3H, CH_3_^Et^), 1.78 (s, 3H, CH_3_^T^), 2.22 ‑ 2.37 (m, 2H, H-2’), 3.56 - 3.67 (m, 2H, H-5’), 3.90 (s, 0.5H, H-4’), 3.93 (s, 0.5H, H-4’), 4.21 (br s, 0.5H, H-3’), 4.57 - 4.75 (m, 2.5H, CH_2_^Et^, H-3’), 5.10-5.17 (m, 1H, OH-5’), 6.22 (app t, J = 6.3 Hz, 1H, H-1’), 7.74 (s, 1H, H-6), 8.96 (d, J_3’,NH_ = 6.3 Hz, 0.5H, NH-3’), 9.15 (d, J_3’,NH_ = 6.3 Hz, 0.5H, NH-3’), 11.30 (s, 1H, NH^T^) ppm.

^13^C NMR (100 MHz, DMSO-d_6_): δ = 12.3 (CH_3_^T^), 15.6 (CH_3_^Et^), 15.7 (CH_3_^Et^), 37.6 (C-2’), 37.9 (C-2’), 54.1 (C-3’), 54.4 (C-3’), 61.3 (C-5’), 61.4 (C-5’), 69.1 (CH_2_^Et^), 83.48 (C-1’), 83.53 (C‑1’), 84.5 (C-4’), 84.9 (C-4’), 109.5 (C-5), 136.3 (C-6), 150.4 (C-2), 163.8 (C-4), 171.8 (C‑Sq2), 172.2 (C-Sq2), 177.1 (C-Sq1), 177.5 (C‑Sq1), 182.3 (C-Sq3), 182.8 (C-Sq3), 189.0 (C-Sq4) ppm.

HRMS (APCI^+^): *m/z* calc. 366.1296 [M+H]^+^, found: 366.1296

**3'-*N*-(2-(2-Hydroxyethyl)amino-3,4-dioxocyclobut-1-en-1-yl)amino-3'-deoxythymidine (7)**

3'-*N*-(2-Ethoxy-3,4-dioxocyclobut-1-en-1-yl)amino-3'-deoxythymidine (**6**) (35 mg, 95 μmol) was dissolved in EtOH (0.8 mL) and CH_2_Cl_2_ (1 mL). Ethanolamine (8 μL, 132 μmol) was added. The reaction mixture was stirred at room temperature for 2.5 hours, after which time a white solid precipitate had formed. Water (1 mL) was added to dissolve the precipitate and the reaction mixture was stirred for a further 18 hours. TLC analysis (CH_2_Cl_2_-MeOH, 92:8) after this time showed consumption of starting material (R_f_ = 0.5) and formation of product (R_f_ = 0.0). The solvent was removed under reduced pressure and the residue was purified by flash chromatography (EtOAc‑MeOH, 75:25) to provide the product **7** as a white crystalline solid (34 mg, 94%); mp 176-182 °C.

Note: compound **7** exhibits rotamers in NMR spectroscopy.

ν_max_/cm^-1^ (neat) 3169, 2925, 1801, 1670, 1572, 1536, 1468, 1432, 1348, 1272, 1212, 1090, 1053, 965, 865, 763, 734.

^1^H NMR (400 MHz, DMSO-d_6_): *δ* = 1.21-1.26 (m, 2H, CH_2_-O), 1.79 (s, 3H, CH_3_^T^), 2.26-2.31 (m, 1H, H‑2’a), 2.35-2.41 (m, 1H, H-2’b), 3.50-3.54 (m, 1H x H-5’a, 0.7H, CH_2_-N), 3.54-3.60 (m, 1.3H, CH_2_-N), 3.61‑3.70 (m, 1H, H-5’b), 3.87-3.91 (1H, H-4’), 4.56 (br s, 1H, H-3’), 4.94 (br s, 1H, OH), 5.18 (br s, 1H, OH), 6.20 (app t, J = 6.3 Hz, 1H, H-1’), 7.69 (br s, 1H, NH), 7.77 (s, 1H, H-6), 8.08 (br s, 1H, NH), 11.31 (s, 1H, NH^T^) ppm.

^13^C NMR (100 MHz, DMSO-d_6_): *δ* = 12.3 (CH_3_^T^), 29.0 (CH_2_-O), 38.2 (C-2’), 45.9 (CH_2_-N), 53.6 (C-3’), 60.7 (C-5’), 83.3 (C-1’), 85.5 (C-4’), 109.5 (C-5), 136.1 (C-6), 150.5 (C-2), 163.7 (C-4), 166.8 (C-Sq), 168.5 (C‑Sq), 182.2 (C-Sq), 182.7 (C-Sq) ppm.

HRMS (ESI^+^): *m/z* calc. 403.1224 [M+Na]^+^, found: 403.1220

**3'-*N*-(2-(*N*-Methyl)hydroxylamino-3,4-dioxocyclobut-1-en-1-yl)amino-3'-deoxythymidine (8)**

3'-*N*-(2-Ethoxy-3,4-dioxocyclobut-1-en-1-yl)amino-3'-deoxythymidine (**6**) (31 mg, 86 μmol) was dissolved in EtOH (1 mL) and CH_2_Cl_2_ (1 mL). *N*-Methylhydroxylamine hydrochloride (12 mg, 140 μmol) was added, followed by triethylamine (35 μL, 251 μmol). The reaction mixture was stirred at room temperature for 24 hours. TLC analysis (EtOAc-MeOH, 75:25) after this time showed complete consumption of starting material (R_f_ = 0.8) and formation of product (R_f_ = 0.3). The solvent was removed under reduced pressure and the residue was purified by flash chromatography (toluene‑acetone, 1:9) to provide the product **8** as a white foam (24 mg, 76%).

ν_max_/cm^-1^ (neat) 3231, 2917, 1689, 1656, 1546, 1453, 1402, 1376, 1270, 1094, 1069, 884, 813, 786, 716, 676.

^1^H NMR (400 MHz, D_2_O): *δ* = 1.91 (d, J_6,CH3_ = 1.1 Hz, 3H, CH_3_^T^), 2.59-2.66 (m, 2H, H-2’a, H-2’b), 3.51 (s, 3H, CH_3_-N), 3.82 (dd, J_5’a,5’b_ = 12.8 Hz, J_4’,5’a_ = 4.6 Hz, 1H, H-5’a), 3.93 (dd, J_5’a,5’b_ = 12.8 Hz, J_4’,5’b_ = 2.3 Hz, 1H, H-5’b), 4.07‑4.14 (m, 1H, H-4’), 4.81-4.89 (m, 1H, H‑3’), 6.28 (app t, J = 5.9 Hz, 1H, H-1’), 7.71 (app d, J = 1.1 Hz, 1H, H-6) ppm.

^13^C NMR (100 MHz, D_2_O): *δ* = 12.5 (CH_3_^T^), 38.4 (C-2’), 42.0 (CH_3_-N), 54.3 (C-3’), 61.2 (C-5’), 85.0 (C-1’), 85.6 (C-4’), 112.4 (C-5), 138.8 (C-6), 152.6 (C-2), 165.5 (C-Sq), 166.7 (C-Sq), 167.6 (C-4), 178.4 (C-Sq), 179.2 (C-Sq) ppm.

HRMS (ESI^+^): *m/z* calc. 389.1068 [M+Na]^+^, found: 389.1067

**3'-*N*-(2-Hydroxy-3,4-dioxocyclobut-1-en-1-yl)amino-3'-deoxythymidine**^1^ **(9)**

3'-*N*-(2-Ethoxy-3,4-dioxocyclobut-1-en-1-yl)amino-3'-deoxythymidine (**6**) (48 mg, 132 μmol) was dissolved in EtOH (1.3 mL) and water (0.3 mL). NaOH (18 mg, 447 μmol) was added, and the reaction mixture was stirred at room temperature for 4 hours. TLC analysis (EtOAc-MeOH, 90:10) after this time showed formation of product (R_f_ = 0.0), but a large amount of starting material (R_f_ = 0.5) remaining. Additional NaOH (17 mg, 425 μmol) was added, and the reaction mixture was stirred at room temperature for a further 1.5 hours. TLC analysis after this time showed complete consumption of starting material and formation of product. The reaction mixture was eluted through Dialon WT01S ion-exchange resin (H form) and then eluted through Dialon WT01S ion‑exchange resin (Na form). The solvent was removed under reduced pressure, and the residue was purified by flash chromatography (water‑*i*PrOH‑EtOAc, 4:56:40) to obtain the product **9** as a white crystalline solid (10 mg, 22%); mp 112-118 °C.

ν_max_/cm^-1^ (neat) 3368, 2508, 1651, 1618, 1529, 1499, 1469, 1445, 1239, 1096, 1070, 897, 766.

^1^H NMR (400 MHz, D_2_O): *δ* = 1.86 (d, J_6,CH3_ = 1.0 Hz, 3H, CH_3_^T^), 2.52-2.57 (m, 2H, H-2’a, H-2’b), 3.76 (dd, J_5’a,5’b_ = 13.0 Hz, J_4’,5’a_ = 4.5 Hz, 1H, H-5’a), 3.86 (dd, J_5’a,5’b_ = 13.0 Hz, J_4’,5’b_ = 2.6 Hz, 1H, H-5’b), 4.01 (ddd, J_3’,4’_ = 7.9 Hz, J_4’,5’a_ = 4.5 Hz, J_4’,5’b_ = 2.6 Hz, 1H, H-4’), 4.73-4.79 (m, 1H, H-3’), 6.23 (app t, J = 5.9 Hz, 1H, H-1’), 7.69 (app d, J = 1.0 Hz, 1H, H-6) ppm.

^13^C NMR (100 MHz, D_2_O): *δ* = 11.5 (CH_3_^T^), 37.4 (C-2’), 52.3 (C-3’), 60.0 (C-5’), 84.3 (C-1’), 84.4 (C-4’), 111.3 (C-5), 137.7 (C-6), 151.6 (C-2), 166.5 (C-4), 180.6 (C-Sq), 188.3 (C-Sq), 195.2 (C-Sq) ppm.

HRMS (APCI^-^): *m/z* calc. 336.0837 [M-Na]^-^, found: 336.0837

## Uridine 3’-squaramides

**2',5'-Bis-*O*-(*tert*-butyldimethylsilyl)uridine**^2^ **(11)**

Uridine (**10**) (5.01 g, 20.5 mmol) was dissolved in pyridine (42 mL). TBDMSCl (9.73 g, 64.6 mmol) was added. The reaction mixture was stirred at room temperature under argon for 17 hours. TLC analysis (CH_2_Cl_2_-MeOH, 95:5) after this time showed formation of the desired product (R_f_ = 0.5), but incomplete consumption of starting material (R_f_ = 0.0). Another portion of TBDMSCl (1.53 g, 10.2 mmol) was added and the reaction mixture was stirred under argon for a further 4.5 hours. After this time another portion of TBDMSCl (1.51 g, 10.0 mmol) was added and the reaction mixture was stirred for a further 2.5 hours. Another portion of TBDMSCl (1.51 g, 10.0 mmol) was added and the reaction was stirred for a further 17.5 hours. The reaction was then quenched by addition of MeOH (5 mL), and the solvent was removed under reduced pressure. The residue was purified by flash chromatography (CH_2_Cl_2_‑MeOH, 98:2), and the product **11** was obtained as a white foam (8.60 g, 89%).

Note: compound **11** exhibits rotamers in NMR spectroscopy.

ν_max_/cm^-1^ (neat) 2962, 2929, 2859, 1685, 1460, 1254, 1123, 1063, 1002, 831, 777.

^1^H NMR (600 MHz, DMSO-d_6_): *δ* = 0.00-0.11 (m, 12H, 4 x CH_3_^TBDMS^), 0.81-0.93 (m, 18H, 2 x *t*Bu^TBDMS^), 3.64‑3.98 (m, 3.6H, 0.6 x H-3’, H-4’, 2 x H-5’), 4.09 ( m, 1.4H, H-2', 0.4 x H-3’), 5.09 (d, J_OH‑3',3'_ = 5.4 Hz, 0.6H, OH-3'), 5.36 (d, J_OH‑3',3'_ = 5.4 Hz, 0.4H, OH-3’), 5.60 (d, J_5,6_ = 8.1 Hz, 1H, H-5), 5.76 (d, J_1',2'_ = 4.9 Hz, 0.4H, H-1'), 5.81 (d, J_1',2'_ = 4.9 Hz, 0.6H, H-1'), 7.73 (d, J_5,6_ = 8.1, 0.4H, H-6), 7.80 (d, J_5,6_ = 8.1, 0.6H, H‑6), 11.37 (s, 1H, NH) ppm.

^13^C NMR (151 MHz, DMSO-d_6_): *δ* = -5.60 (CH_3_^TBDMS^), -5.56 (CH_3_^TBDMS^), -5.2 (CH_3_^TBDMS^), -5.1 (CH_3_^TBDMS^), ‑4.8 (CH_3_^TBDMS^), -4.5 (CH_3_^TBDMS^), 17.87 (qC, *t*Bu^TBMDS^), 17.99 (qC, *t*Bu^TBMDS^), 18.01 (qC, *t*Bu^TBMDS^), 18.03 (qC, tBu^TBMDS^), 25.6 (tBu^TBDMS^), 25.8 (tBu^TBDMS^), 62.4 (C-5’), 62.6 (C-5’), 69.6 (C-3’), 71.6 (C-3’), 72.7 (C‑2’), 75.9 (C-2’), 84.5 (C-4’), 87.6 (C-1’), 87.7 (C-1’), 101.66 (C-5), 101.71 (C-5), 139.7 (C-6), 140.3 (C‑6), 150.5 (C-2), 150.7 (C-2), 162.9 (C-4), 163.0 (C-4) ppm.

HRMS (ESI^+^): *m/z* calc. 473.2498 [M+H]^+^, found: 473.2499

**2',5'-Bis-*O*-(*tert*-butyldimethylsilyl)-3'-oxouridine**^3^ **(12)**

2',5'-Bis-*O*-(*tert*-butyldimethylsilyl)uridine (**11**) (2.16 g, 4.56 mmol) was dissolved in anhydrous CH_2_Cl_2_ (14 mL). A solution of Dess-Martin periodinane (3.10 g, 7.31 mmol) in anhydrous CH_2_Cl_2_ (50 mL) was added to the reaction mixture at 0 °C. The reaction mixture was stirred under argon at 0 °C for 15 minutes, and then at room temperature for a further 20 hours. TLC analysis (petroleum ether-EtOAc, 1:1) after this time showed complete consumption of starting material (R_f_ = 0.3) and formation of product (R_f_ = 0.5). The reaction mixture was diluted with EtOAc (130 mL) and washed with an ice cold aqueous solution of 10% (w/v) sodium thiosulfate (78 mL), followed by an ice cold saturated aqueous NaHCO_3_ solution (130 mL). The organic layer was dried over MgSO_4_ and filtered. The solvent was removed under reduced pressure to provide the pure product **12** as a white foam (2.14 g, 99%).

ν_max_/cm^-1^ (neat) 3109, 2954, 2930, 2858, 1766, 1686 1463, 1410, 1381, 1263, 1083, 897, 834, 777, 667.

^1^H NMR (600 MHz, DMSO-d_6_): *δ* = -0.030 (s, 3H, CH_3_^TBDMS^), 0.031 (s, 3H, CH_3_^TBDMS^), 0.033 (s, 3H, CH_3_^TBDMS^), 0.06 (s, 3H, CH_3_^TBDMS^), 0.82 (s, 9H, *t*Bu^TBDMS^), 0.85 (s, 9H, *t*Bu^TBDMS^), 3.83 (d, J_4',5'_ = 2.8 Hz, 2H, H-5'), 4.26 (d, J_1',2'_ = 8.0 Hz, 1H, H-2'), 4.42 (t, J_4',5'_ = 2.8 Hz, 1H, H-4'), 5.84 (dd, J_5,6_ = 8.1 Hz, J_NH,5_ = 1.5 Hz, 1H, H-5), 6.21 (d, J_1',2'_ = 8.0 Hz, 1H, H-1'), 7.77 (d, J_5,6_ = 8.1 Hz, 1H, H-6), 11.56 (br s, 1H, NH) ppm.

^13^C NMR (151 MHz, DMSO-d_6_): *δ* = -5.8 (CH_3_^TBDMS^), -5.6 (CH_3_^TBDMS^), -5.5 (CH_3_^TBDMS^), -4.9 (CH_3_^TBDMS^), 17.85 (qC, *t*Bu^TBMDS^), 17.88 (qC, *t*Bu^TBMDS^), 25.2 (*t*Bu^TBDMS^), 25.6 (*t*Bu^TBDMS^), 62.6 (C-5'), 76.4 (C-2'), 81.7 (C-4'), 84.0 (C-1'), 103.2 (C-5), 139.1 (C-6), 150.5 (C-2), 162.6 (C-4), 208.4 (C-3') ppm.

HRMS (APCI^+^): *m/z* calc. 471.2341 [M+H]^+^, found: 471.2342

**2',5'-Bis-*O*-(*tert*-butyldimethylsilyl)-3'-hydroxyimino-3'-deoxyuridine**^4^ **(15/16)**

2',5'-Bis-*O*-(*tert*-butyldimethylsilyl)-3'-oxouridine (**12**) (8.15 g, 17.3 mmol) was dissolved in anhydrous pyridine (160 mL). Hydroxylamine hydrochloride (6.22 g, 89.6 mmol) was added. The reaction mixture was stirred at room temperature under argon for 17 hours. After this time TLC analysis (petroleum ether-EtOAc, 1:1, visualised under UV irradiation) showed consumption of starting material (R_f_ = 0.7, turns brown upon heating) and formation of product (R_f_ = 0.7, does not change colour upon heating). The solvent was removed under reduced pressure and the residue was co-evaporated with toluene (150 mL), and then redissolved in EtOAc (300 mL) and water (300 mL). The organic layer was separated, dried over MgSO_4_, and filtered. The solvent was removed under reduced pressure, and the residue was purified by flash chromatography (petroleum ether-EtOAc, 3:1) to obtain the product as a mixture of isomers **15** and **16** as a white foam (7.26 g, 86%). A side product, 3',5'‑bis‑O‑(tert‑butyldimethylsilyl)-2'-hydroxyimino-3'-deoxyuridine (**13**/**14**) was also obtained in a trace amount as a mixture of E/Z isomers as a white foam. The dominant isomer was isolated for characterisation.

2',5'-Bis-*O*-(*tert*-butyldimethylsilyl)-3'-hydroxyimino-3'-deoxyuridine^4^ (**15**/**16**)

ν_max_/cm^-1^ (neat) 3234, 2930, 2858, 1658, 1461, 1382, 1276, 1252, 1110, 1071, 952, 895, 834, 778, 718, 672.

^1^H NMR (600 MHz, DMSO-d_6_): Isomer A: *δ* = -0.04-0.08 (m, 12H, CH_3_^TBDMS^), 0.81-0.84 (m, 18H, *t*Bu^TBDMS^), 3.77-3.82 (m, 1H, H-5’a), 3.91 (dd, J_5’a,5’b_ = 11.6 Hz, J_4’,5’a_ = 2.1 Hz, H-5’b), 4.68-4.72 (m, 1H, H-2’), 4.92‑4.93 (m, 1H, H-4’), 5.63 (d, J_5,6_ = 8.0 Hz, H-5, 5.77 (d, J_1’,2’_ = 4.1 Hz, 1H, H-1’), 7.74 (d, J_5,6_ = 8.0 Hz, 1H, H-6), 11.34 (s, 1H, NH), 11.48 (br s, 1H, N-OH) ppm. Isomer B: *δ* = -0.04-0.08 (m, 12H, CH_3_^TBDMS^), 0.81‑0.84 (m, 18H, *t*Bu^TBDMS^), 3.84 (dd, J_5’a,5’b_ = 11.3 Hz, J_4’,5’a_ = 1.6 Hz, 1H, H-5’a), 4.02 (dd, J_5’a,5’b_ = 11.3 Hz, J_4’,5’b_ = 2.2 Hz, 1H, H-5’b), 4.59 (dd, J_1’,2’_ = 7.4 Hz, J_2’,4’_ = 1.6 Hz, 1H, H-2’), 4.94-4.95 (m, 1H, H-4’), 5.77 (d, J_5,6_ = 8.0 Hz, 1H, H-5), 5.91 (d, J_1’,2’_ = 7.4 Hz, 1H, H-1’), 7.78 (d, J_5,6_ = 8.0 Hz, 1H, H-6), 11.47 (s, 1H, NH), 11.48 (br s, 1H, N-OH) ppm .

^13^C NMR (100 MHz, DMSO-d_6_): *δ* = -5.6 (CH_3_^TBDMS^), -5.42 (CH_3_^TBDMS^), -5.41 (CH_3_^TBDMS^), -5.3 (CH_3_^TBDMS^), ‑5.2 (CH_3_^TBDMS^), -4.9 (CH_3_^TBDMS^), -4.7 (CH_3_^TBDMS^), 18.0 (qC, *t*Bu^TBMDS^), 18.1 (qC, *t*Bu^TBMDS^), 18.3 (qC, *t*Bu^TBMDS^), 25.4 (*t*Bu^TBDMS^), 25.6 (*t*Bu^TBDMS^), 25.8 (*t*Bu^TBDMS^), 25.9 (*t*Bu^TBDMS^), 62.1 (C-5’ isomer B), 64.5 (C‑5’ isomer A), 70.6 (C-4’ isomer A), 74.1 (C-2’ isomer B), 77.3 (C-4’ isomer B), 79.1 (C-2’ isomer A), 86.0 (C‑1’ isomer B), 91.2 (C-1’ isomer A), 102.4 (C-5 isomer A), 103.1 (C-5 isomer B), 139.4 (C‑6 isomer B), 141.6 (C-6 isomer A), 150.6 (C-2 isomer A), 150.7 (C-2 isomer B), 154.4 (C-3’ isomer A), 155.5 (C‑3’ isomer B), 162.9 (C-4 isomer B), 163.2 (C-2 isomer A) ppm.

HRMS (APCI^+^): *m/z* calc. 486.2450 [M+H]^+^, found: 486.2446

3',5'-bis-O-(tert-butyldimethylsilyl)-2'-hydroxyimino-3'-deoxyuridine (**13**/**14**)

ν_max_/cm^-1^ (neat) 3228, 2953, 2929, 2857, 1686, 1461, 1388, 1253, 1091, 982, 954, 901, 832, 777, 671, 630.

^1^H NMR (600 MHz, DMSO-d_6_): 0.06 (s, 6H, 2 x CH_3_^TBDMS^), 0.1 (s, 3H, CH_3_^TBDMS^), 0.14 (s, 3H, CH_3_^TBDMS^), 0.86 (s, 18H, *t*Bu^TBDMS^), 3.80 (d, J_4’,5’_ = 3.4 Hz, 2H, H-5’a, H-5’b), 4.01 (dd, J_3’,4’_ = 6.6 Hz, J_4’,5’_ = 3.4 Hz, 1H, H-4’), 4.89-4.91 (m, 1H, H-3’), 5.62 (d, J_5,6_ = 8.1 Hz, 1H, H‑5), 6.54 (s, 1H, H-1’), 7.54 (d, J_5,6_ = 8.1 Hz, 1H, H-6), 11.47 (br s, 1H, NH^U^), 11.80 (s, 1H, N-OH) ppm .

^13^C NMR (151 MHz, DMSO-d_6_): *δ* = -5.61 (CH_3_^TBDMS^), -5.60 (CH_3_^TBDMS^), -5.2 (CH_3_^TBDMS^), -4.9 (CH_3_^TBDMS^), 17.9 (qC, *t*Bu^TBMDS^), 18.0 (qC, *t*Bu^TBMDS^), 25.6 (*t*Bu^TBDMS^), 25.7 (*t*Bu^TBDMS^), 63.2 (C-5’), 65.8 (C-3’), 81.4 (C‑1’), 85.9 (C‑4’), 102.2 (C-5), 141.7 (C-6), 150.5 (C-2), 154.9 (C-2’), 162.8 (C-4) ppm.

HRMS (APCI^+^): *m/z* calc. 486.2450 [M+H]^+^, found: 486.2455

**2'-*O*-(*tert*-Butyldimethylsilyl)-3'-hydroxyimino-3'-deoxyuridine**^4^ **(17, 18)**

2',5'-Bis-*O*-(*tert*-butyldimethylsilyl)-3'-hydroxyimino-3'-deoxyuridine (**15**/**16**) (6.19 g, 12.7 mmol) was dissolved in a mixture of THF (93 mL) and water (68 mL). Trifluoroacetic acid (12 mL) was added, and the reaction mixture was stirred at 0°C for 4.5 hours. TLC analysis after this time (CH_2_Cl_2_-MeOH, 90:10) showed consumption of starting material (R_f_ = 0.6) and formation of product isomers **17** (R_f_ = 0.46) and **18** (R_f_ = 0.38). The reaction was then quenched by addition of saturated aqueous NaHCO_3_ solution (300 mL). The reaction mixture was extracted with EtOAc (300 mL), and the organic layer was then washed with saturated aqueous NaHCO_3_ solution (3 x 250 mL), followed by water (200 mL) and brine (200 mL). The organic layer was dried over MgSO_4_ and filtered. The solvent was removed under reduced pressure. The residue was purified by flash chromatography (CH_2_Cl_2_-MeOH, 96:4) to obtain the product as a mixture of isomers **17**/**18** as a white foam (3.41 g, 72%). A small amount of each isomer was isolated for separate characterisation.

2'-*O*-(*tert*-Butyldimethylsilyl)-3'-hydroxyimino-3'-deoxyuridine (**17**)

ν_max_/cm^-1^ (neat) 3296, 2929, 2867, 1677, 1460, 1392, 1249, 1149, 1086, 958, 836, 779, 673.

^1^H NMR (600 MHz, DMSO-d_6_): *δ* = -0.03 (s, 3H, CH_3_^TBDMS^), 0.04 (s, 3H, CH_3_^TBDMS^), 0.81 (s, 9H, *t*Bu^TBDMS^), 3.67 (ddd, J_5'a,5'b_ = 11.7 Hz, J_5'a,OH-5'_ = 5.4 Hz, J_4',5'a_ = 2.2 Hz, 1H, H-5'a), 3.83 (ddd, J_5'a,5'b_ = 11.7 Hz, J_5'b,OH‑5'_ = 5.4 Hz, J_4',5'b_ = 3.0 Hz, 1H, H-5'b), 4.70 (dd, J_1',2'_ = 7.5 Hz, J_2',4'_ = 1.7 Hz, 1H, H-2'), 4.86 (m, 1H, H-4'), 5.18 (app t, J = 5.4 Hz, 1H, OH-5'), 5.80 (dd, J_5,6_ = 8.1 Hz, J_5,NH_ = 1.8 Hz, 1H, H-5), 5.85 (d, J_1'2'_ = 7.5 Hz, 1H, H-1'), 7.94 (d, J_5,6_ = 8.1 Hz, H-6), 11.35 (s, 1H, N-OH), 11.47 (d, J_5,NH_ = 1.8 Hz, NH^U^) ppm.

^13^C NMR (151 MHz, DMSO-d_6_): *δ* = -5.5 (CH_3_^TBDMS^), -4.8 (CH_3_^TBDMS^), 17.8 (qC, *t*Bu^TBMDS^), 25.4 (*t*Bu^TBDMS^), 59.8 (C-5'), 73.3 (C-2'), 77.6 (C-4'), 85.9 (C-1'), 103.0 (C-5), 140.2 (C-6), 150.7 (C-2), 155.9 (C-3'), 162.8 (C-4) ppm.

HRMS (APCI^-^): *m/z* calc. 370.1439 [M+H]^-^, found: 370.1448

2'-*O*-(*tert*-Butyldimethylsilyl)-3'-hydroxyimino-3'-deoxyuridine (**18**)

ν_max_/cm^-1^ (neat) 3237, 2930, 2858, 1681, 1462, 1382, 1275, 1252, 1109, 950, 894, 835, 813, 779.

^1^H NMR (400 MHz, DMSO-d_6_): *δ* = 0.04 (s, 3H, CH_3_^TBDMS^), 0.07 (s, 3H, CH_3_^TBDMS^), 0.81 (s, 9H, *t*Bu^TBDMS^), 3.57-3.63 (m, 1H, H-5'a), 3.73 (ddd, J_5'a,5'b_ = 12.1 Hz, J_5'b,OH-5'_ = 5.4 Hz, J_5'b,4'_ = 2.4 Hz, 1H, H-5'b), 4.64‑4.65 (m, 1H, H‑4'), 4.95-4.98 (m, 2H, H-2', OH-5'), 5.67 (d, J_5,6_ = 8.0 Hz, 1H, H-5), 5.77 (d, J_1',2'_ = 4.3 Hz, 1H, H-1'), 7.82 (d, J_5,6_ = 8.0 Hz, H-6), 11.30 (s, 1H, N-OH), 11.44 (br s, 1H, NH) ppm.

^13^C NMR (100 MHz, DMSO-d_6_): *δ* = -5.3 (CH_3_^TBDMS^), -5.1 (CH_3_^TBDMS^), 17.8 (qC, *t*Bu^TBMDS^), 25.5 (*t*Bu^TBDMS^), 62.0 (C-5'), 70.3 (C-2'), 79.2 (C-4'), 90.6 (C-1'), 102.2 (C-5), 141.6 (C-6), 150.5 (C-2), 154.5 (C-3'), 163.0 (C-4) ppm.

HRMS (APCI^-^): *m/z* calc. 370.1439 [M+H]^-^, found: 370.1437

**2'-*O*-(*tert*-Butyldimethylsilyl)-3'-hydroxyamino-3'-deoxyuridine**^4^ **(20)**

2'-*O*-(*tert*-Butyldimethylsilyl)-3'-hydroxyimino-3'-deoxyuridine (**17**/**18**) (461 mg, 1.24 mmol) was dissolved in AcOH (16 mL). NaBH_4_ (196 mg, 5.19 mmol) was added. The reaction mixture was stirred at room temperature for 2.5 hours. TLC analysis (CH_2_Cl_2_-MeOH, 93:7) after this time showed consumption of starting material (R_f_ = 0.4) and formation of product (R_f_ = 0.3). The reaction mixture was then diluted with EtOAc (50 mL) and washed with saturated aqueous NaHCO_3_ solution (3 x 50 mL), water (50 mL), and brine (50 mL). The organic layer was dried over MgSO_4_ and filtered. The solvent was removed under reduced pressure and the residue was purified by flash chromatography (CH_2_Cl_2_‑MeOH, 94:6) to provide the product **20** as a white foam (391 mg, 84%). A side product, 2'‑*O*‑(*tert*-butyldimethylsilyl)-3'-(*N*-hydroxy-*N*-ethyl)amino-3'-deoxyuridine (**19**), was obtained in a trace amount as a white foam.

ν_max_/cm^-1^ (neat) 3363, 2934, 2859, 1676, 1461, 1386, 1255, 1104, 1052, 842, 779.

^1^H NMR (600 MHz, DMSO-d_6_): *δ* = 0.03 (s, 3H, CH_3_^TBDMS^), 0.06 (s, 3H, CH_3_^TBDMS^), 0.85 (s, 9H, *t*Bu^TBDMS^), 3.38 (app t, J = 5.1 Hz, 1H, H-4'), 3.53-3.55 (m, 1H, H-5'a), 3.69-3.72 (m, 1H, H-5'b), 3.98 (m, 1H, H-3'), 4.32 (app t, J = 4.9 Hz, 1H, H-2'), 5.23 (app t, J = 4.6 Hz, 1H, NH-3’), 5.41 (br s, 1H, N-OH), 5.67 (d, J_5,6_ = 8.1 Hz, 1H, H-5), 5.82 (d, J_1',2'_ = 4.9 Hz, 1H, H-1'), 7.58 (s, 1H, OH-5'), 8.02 (d, J_5,6_ = 8.1 Hz, 1H, H-6), 11.33 (s, 1H, NH^U^) ppm.

^13^C NMR (151 MHz, DMSO-d_6_): *δ* = -5.4 (CH_3_^TBDMS^), -5.2 (CH_3_^TBDMS^), 17.7 (qC, *t*Bu^TBMDS^), 25.6 (*t*Bu^TBDMS^), 61.5 (C-5'), 62.7 (C-4'), 74.5 (C-2'), 81.9 (C-3'), 88.4 (C-1'), 101.8 (C-5), 140.3 (C-6), 150.6 (C-2), 163.1 (C-4) ppm.

HRMS (APCI^-^): *m/z* calc. 372.1596 [M-H]^-^, found: 372.1596

2'-*O*-(*tert*-Butyldimethylsilyl)-3'-(*N*-hydroxy-*N*-ethyl)amino-3'-deoxyuridine (**19**)

ν_max_/cm^-1^ (neat) 3442, 3055, 2932, 2858, 1674, 1464, 1387, 1255, 1146, 1112, 1082, 836, 778, 760.

^1^H NMR (600 MHz, DMSO-d_6_): *δ* = 0.01 (s, 3H, CH_3_^TBDMS^), 0.05 (s, 3H, CH_3_^TBDMS^), 0.83 (s, 9H, *t*Bu^TBDMS^), 1.02 (t, J_CH2,CH3_ = 6.9 Hz, 3H, CH_3_^Et^), 2.62 (dq, J_CH2Eta,CH2Etb_ = 13.7 Hz, J_CH2,CH3_ = 6.9 Hz, 1H, CH_2_^Et^-a), 2.82 (dq, J_CH2Eta,CH2Etb_ = 13.7 Hz, J_CH2,CH3_ = 6.9 Hz, 1H, CH_2_^Et^-b), 3.15 (dd, J_2’,3’_ = 6.2 Hz, J_3’,4’_ = 3.9 Hz, 1H, H-3’), 3.57 (d, J_5’a,5’b_ = 10.9 Hz, 1H, H-5’a), 3.68 (d, J_5’a,5’b_ = 10.9 Hz, 1H, H-5’b), 4.25 (app t, J = 6.2 Hz, 1H, H‑2’), 4.38-4.41 (m, 1H, H-4’), 5.11 (br s, 1H, OH-5’), 5.67 (d, J_5,6_ = 8.1 Hz, 1H, H-5), 5.93 (d, J_1’,2’_ = 6.2 Hz, 1H, H-1’), 7.72 (s, 1H, N‑OH), 7.97 (d, J_5,6_ = 8.1 Hz, 1H, H-6), 11.29 (s, 1H, NH^U^) ppm.

^13^C NMR (151 MHz, DMSO-d_6_): *δ* = -5.1 (CH_3_^TBDMS^), -5.0 (CH_3_^TBDMS^), 12.9 (CH_3_^Et^), 17.9 (qC, *t*Bu^TBMDS^), 25.6 (*t*Bu^TBDMS^), 51.6 (CH_2_^Et^), 62.7 (C-5’), 66.0 (C-3’), 75.7 (C-2’), 80.1 (C-4’), 87.8 (C-1’), 101.8 (C-5), 140.3 (C‑6), 150.8 (C-2), 163.1 (C-4) ppm.

HRMS (APCI^+^): *m/z* calc. 402.2055 [M+H]^+^, found: 402.2050

**2'-*O*-(*tert*-Butyldimethylsilyl)-3'-amino-3'-deoxyuridine (21)**

2'-*O*-(*tert*-Butyldimethylsilyl)-3'-hydroxyamino-3'-deoxyuridine (**20**) (360 mg, 0.74 mmol) was dissolved in a mixture of AcOH (8.9 mL) and water (1 mL). The solution was degassed by bubbling with argon. 10% Pd/C (75 mg) was added. The reaction vessel was purged with H_2_, and the reaction mixture was stirred under an atmosphere of H_2_ for 6 hours. TLC analysis (CH_2_Cl_2_-MeOH, 90:10) after this time showed complete consumption of starting material (R_f_ = 0.4) and formation of product (R_f_ = 0.3). The reaction mixture was filtered through celite, washing through with MeOH. The solvent was removed under reduced pressure. The residue was co-evaporated with toluene (50 mL) and then purified by flash chromatography (CH_2_Cl_2_‑MeOH-NEt_3_, 91:8:1) to afford the product **21** as a white foam (250 mg, 73%).

ν_max_/cm^-1^ (neat) 3371, 2930, 2863, 1685, 1460, 1378, 1254, 1105, 1060, 993, 838, 779, 699.

^1^H NMR (600 MHz, DMSO-d_6_): *δ* = 0.09 (s, 3H, CH_3_^TBDMS^), 0.11 (s, 3H, CH_3_^TBDMS^), 0.88 (s, 9H, *t*Bu^TBDMS^), 3.20 (dd, J_3',4'_ = 7.6 Hz, J_2',3'_ = 4.9 Hz, 1H, H-3'), 3.60-3.67 (m, 2H, H-5'a, H-4'), 3.73-3.76 (m, 1H, H-5'b), 4.06 (dd, J_2',3'_ = 4.9 Hz, J_1',2'_ = 2.1 Hz, 1H, H-2'), 5.12 (br s, 1H, OH-5'), 5.59 (d, J_5,6_ = 8.1 Hz, 1H, H-5), 5.66 (d, J_1',2'_ = 2.1 Hz, 1H, H-1'), 8.07 (d, J_5,6_ = 8.1 Hz, 1H, H-6) ppm.

^13^C NMR (151 MHz, DMSO-d_6_): *δ* = -5.1 (CH_3_^TBDMS^), -4.8 (CH_3_^TBDMS^), 17.8 (qC, *t*Bu^TBMDS^), 25.7 (*t*Bu^TBDMS^), 51.8 (C-3'), 59.6 (C-5'), 77.1 (C-2'), 85.1 (C-4'), 89.3 (C-1'), 101.1 (C-5), 140.4 (C-6), 150.5 (C-2), 163.2 (C-4) ppm.

HRMS (APCI^-^): *m/z* calc. 356.1647 [M+H]^-^, found: 356.1638

**2'-*O*-(*tert*-Butyldimethylsilyl)-3'-*N*-(2-ethoxy-3,4-dioxocyclobut-1-en-1-yl)amino-3'-deoxyuridine (22)**

2'-*O*-(*tert*-Butyldimethylsilyl)-3'-amino-3'-deoxyuridine (**21**) (56 mg, 0.16 mmol) was dissolved in EtOH (1 mL). Diethyl squarate (35 µL, 0.24 mmol) was added, and the reaction mixture was stirred at room temperature under argon for 17.5 hours. TLC analysis (CH_2_Cl_2_‑MeOH, 90:10) after this time showed formation of product (R_f_ = 0.4) and consumption of starting material (R_f_ = 0.3). The solvent was removed under reduced pressure, and the residue was purified by flash chromatography (CH_2_Cl_2_‑MeOH, 97:3) to obtain the product **22** as a white foam (73 mg, 98%).

Note: compound **22** exhibits rotamers in NMR spectroscopy.

ν_max_/cm^-1^ (neat) 3224, 2931, 2858, 1807, 1681, 1595, 1464, 1428, 1380, 1333, 1264, 1216, 1090, 1061, 835, 778.

^1^H NMR (600 MHz, DMSO-d_6_): *δ* = -0.082 (s, 1.5H, CH_3_^TBDMS^ ), ‑0.078 (s, 1.5H, CH_3_^TBDMS^), -0.038 (s, 1.5H, CH_3_^TBDMS^ ), -0.035 (s, 1.5H, CH_3_^TBDMS^ ), -0.77 (s, 4.5H, *t*Bu^TBDMS^), -0.78 (s, 4.5H, *t*Bu^TBDMS^), 1.33 (t, J_CH2,CH3_ = 7.0 Hz, 1.5H, CH_3_^Et^), 1.37 (t, J_CH2,CH3_ = 7.0 Hz, 1.5H, CH_3_^Et^), 3.57-3.61 (m, 1H, H-5'a), 3.65-3.70 (m, 1H, H-5'b), 4.09-4.15 (m, 1.5H, H-3', 0.5 x H-4'), 4.21-4.34 (m, 1H, H-2’), 4.57-4.73 (m, 2.5H, CH_2_^Et^, 0.5 x H‑4'), 5.29 (dd, J_5’a,OH‑5’_ = 11.7 Hz, J_5’a,OH-5’_ = 5.1 Hz, 1H, OH-5'), 5.71-5.74 (2 x d, 1H, H‑5), 5.93 (d, J_1’,2’_ = 5.1 Hz, 0.5H, H-1') 5.97 (d, J_1’,2’_ = 5.9 Hz, 0.5H, H-1'), 7.93 (app t, J = 9.0 Hz, 1H, H-6), 9.03 (d, J_3’,NHSq_ = 9.3 Hz, 0.5H, NH^Sq^), 9.24 (d, J_3’,NHSq_ = 7.9 Hz, 0.5H, NH^Sq^), 11.43 (s, 1H, NH^U^) ppm.

^13^C NMR (151 MHz, DMSO-d_6_): *δ* = -5.5 (CH_3_^TBDMS^), -5.4 (CH_3_^TBDMS^), -5.3 (CH_3_^TBDMS^), -5.2 (CH_3_^TBDMS^), 15.5 (CH_3_^Et^), 15.7 (CH_3_^Et^), 17.45 (qC, *t*Bu^TBMDS^), 17.49 (qC, *t*Bu^TBMDS^), 25.3 (*t*Bu^TBDMS^), 55.3 (C-4'), 55.4 (C‑4'), 60.2 (C-5'), 60.8 (C-5'), 69.0 (CH_2_^Et^), 69.1 (CH_2_^Et^), 74.6 (C-2'), 75.0 (C-2'), 82.1 (C-3'), 82.5 (C-3'), 86.8, (C‑1'), 87.3 (C-1'), 102.1 (C-5), 102.4 (C-5), 140.1 (C-6), 140.2 (C-6), 150.6 (C-2), 150.7 (C-2), 162.88 (C‑4), 162.91 (C-4), 172.5 (C-Sq2), 172.8 (C-Sq2), 176.8 (C-Sq1), 177.1 (C-Sq1), 182.3 (C-Sq3), 182.5 (C‑Sq3), 188.7 (C-Sq4), 189.2 (C-Sq4) ppm.

HRMS (APCI^-^): *m/z* calc. 480.1807 [M+H]^-^, found: 480.180

**3'-*N*-(2-Ethoxy-3,4-dioxocyclobut-1-en-1-yl)amino-3'-deoxyuridine (23)**

2'-*O*-(*tert*-Butyldimethylsilyl)-3'-*N*-(2-ethoxy-3,4-dioxocyclobut-1-en-1-yl)amino-3'-deoxyuridine (**22**) (64 mg, 133 μmol) was dissolved in THF (1 mL). Tetrabutylammonium fluoride trihydrate (60 mg, 190 μmol) was added and the reaction mixture was stirred at room temperature for 2 hours. TLC analysis (CH_2_Cl_2_‑MeOH, 90:10) after this time showed consumption of starting material (R_f_ = 0.7) and formation of both rotamers of the product (R_f_ = 0.33, 0.41). The solvent was removed under reduced pressure and the residue was purified by flash chromatography (CH_2_Cl_2_-MeOH, 93:7) to obtain the product **23** as a white powder (40 mg, 83%); mp 225-235 °C (decomp).

ν_max_/cm^-1^ (neat) 3214, 2055, 2926, 1806, 1673, 1590, 1425, 1382, 1345, 1258, 1097, 1052, 987, 864, 812, 766.

^1^H NMR (600 MHz, acetone-d_6_): *δ* = 1.40 (t, J_CH2,CH3_ = 7.0 Hz, 3H, CH_3_^Et^), 3.83-3.89 (m, 1H, H-5’a), 3.93‑4.01 (m, 1H, H-5’b), 4.22-4.37 (m, 1H, H-4’), 4.43-4.53 (m, 2H, H-2’, OH-5’), 4.71 (q, J_CH2,CH3_ = 7.0 Hz, 2H, CH_2_^Et^), 4.80 (br s, 1H, H-3’), 5.46 (br s, 1H, OH-2’), 5.63 (d, J_5,6_ = 8.2 Hz, 1H, H-5), 5.91 (d, J_1’,2’_ = 1.9 Hz, 1H, H-1’), 7.60 (br s, 0.5H, NH^Sq^), 7.68 (br s, 0.5H, NH^Sq^), 8.09 (br s, 1H, H-6), 10.07 (br s, 1H, NH^U^) ppm.

^13^C NMR (151 MHz, acetone-d_6_): *δ* = 16.0 (CH_3_^Et^), 55.2 (C-3’), 55.9 (C-3’), 61.1 (C-5’), 70.6 (CH_2_^Et^), 75.8 (C-2’), 83.5 (C-4’), 84.0 (C-4’), 91.2 (C-1’), 102.3 (C-5), 141.2 (C-6), 151.5 (C-2), 163.7 (C-4), 173.5 (C‑Sq1), 174.4 (C-Sq1), 178.6 (C-Sq2), 179.4 (C-Sq2), 183.7 (C-Sq4), 184.5 (C-Sq4), 189.5 (C-Sq3), 190.6 (C-Sq3) ppm.

HRMS (APCI^+^): *m/z* calc. 368.1088 [M+H]^+^, found: 368.1091

**3'-*N*-(2-(2-Hydroxyethyl)amino-3,4-dioxocyclobut-1-en-1-yl)amino-3'-deoxyuridine (24)**

3'-*N*-(2-Ethoxy-3,4-dioxocyclobut-1-en-1-yl)amino-3'-deoxyuridine (**23**) (41 mg, 0.113 mmol) was dissolved in EtOH (2 mL). Ethanolamine (9 μL, 0.147 mmol) was added. The reaction mixture was stirred at room temperature under argon. After 5 minutes a white precipitate formed. After 2 hours TLC analysis (EtOAc‑MeOH, 87:13) showed complete consumption of both rotamers of the starting material (R_f_ = 0.66, 0.73) and formation of product (R_f_ = 0.3). The solvent was removed under reduced pressure. The residue was purified by flash chromatography (EtOAc‑MeOH, 88:12) to afford the product **24** as a white foam (41 mg, 94%).

Note: compound **24** exhibits rotamers in NMR spectroscopy.

ν_max_/cm^-1^ (neat) 3472, 3331, 3169, 2963, 2795, 1804, 1697, 1675, 1648, 1562, 1493, 1466, 1415, 1379, 1357, 1308, 1270, 1206, 1170, 1111, 1046, 945, 868, 830, 710, 626.

^1^H NMR (600 MHz, DMSO-d_6_): *δ* = 3.30 (m, 0.4H, CH_2_-N), 3.50-3.66 (m, 1.6H x CH_2_-N, 2H x CH_2_-O, 1H x H-5’a), 3.72 (dd, J_5’a,5’b_ = 12.1 Hz, J_5’b,OH-5’_ = 5.1 Hz, 1H, H-5’b), 3.93 (br s, 1H, H-4’), 4.21 (br s, 0.2H, H‑2’), 4.26 (br s, 0.8H, H-2’), 4.38 (br s, 0.2H, H-3’), 4.49 (br s, 0.8H, H-3’), 4.94 (m, 1H, OH), 5.24 (app t, J = 5.1 Hz, OH-5’), 5.67 (d, J_5,6_ = 8.1 Hz, 1H, H-5), 5.73 (d, J_1’,2’_ = 3.6 Hz, 0.2H, H-1’), 5.76 (m, 0.8H, H-1’), 6.03 (br s, 0.2H, OH-2’), 6.37 (br s, 0.8H, OH-2’), 7.75 (d, J_3’,NH-3’_ = 7.1 Hz, 1H, NH-3’), 7.84 (br s, 0.2H, NH^Sq^), 7.91 (br s, 0.8H, NH^Sq^), 7.99 (d, J_5,6_ = 8.1 Hz, 1H, H-6), 11.35 (s, 1H, NH^U^) ppm.

^13^C NMR (151 MHz, DMSO-d_6_): *δ* = 45.9 (CH_2_-N), 53.9 (C-3’), 55.2 (C-3’), 60.0 (C-5’), 60.7 (CH_2_-O), 71.0 (C-2’), 73.6 (C-2’), 82.3 (C-4’), 83.8 (C-4’), 89.1 (C-1’), 89.7 (C-1’), 101.6 (C-5), 140.4 (C-6), 150.5 (C-2), 152.9 (C-Sq2), 163.2 (C-4), 166.9 (C-Sq), 168.7 (C-Sq), 182.5 (C-Sq) ppm.

HRMS (APCI^-^): *m/z* calc. 381.1052 [M-H]^-^, found: 381.1050

**3'-*N*-(2-(2-Hydroxyphenyl)amino-3,4-dioxocyclobut-1-en-1-yl)amino-3'-deoxyuridine (25)**

3'-*N*-(2-Ethoxy-3,4-dioxocyclobut-1-en-1-yl)amino-3'-deoxyuridine (**23**) (35 mg, 95 μmol) was dissolved in EtOH (2 mL). 2-Aminophenol (15 mg, 137 μmol) was added. The reaction mixture was stirred at room temperature for 48 hours. More 2-aminophenol (15 mg, 137 μmol) was added, and the reaction mixture was stirred at room temperature for a further 20 hours. 2-Aminophenol (11 mg, 101 μmol) was again added and the reaction mixture was stirred for a further 7 hours. TLC analysis (CH_2_Cl_2_‑MeOH, 90:10) after this time showed consumption of both rotamers of the starting material (R_f_ = 0.33, 0.41) and formation of product (R_f_ = 0.26). The solvent was removed under reduced pressure, and the residue was purified by flash chromatography (CH_2_Cl_2_‑MeOH, 92:8) to provide the product **25** as a brown crystalline solid (35 mg, 85%); mp 199-206 °C.

Note: compound **25** exhibits rotamers in NMR spectroscopy.

ν_max_/cm^-1^ (neat) 3240, 3063, 2971, 2932, 1795, 1672, 1573, 1518, 1454, 1440, 1381, 1256, 1082, 995, 875, 814, 737, 696.

^1^H NMR (600 MHz, DMSO-d_6_): *δ* = 3.54-3.59 (m, 0.2H, H-5’), 3.60-3.64 (m, 0.2H, H-5’), 3.64-3.69 (m, 0.8H, H-5’), 3.71-3.76 (m, 0.8H, H-5’), 3.79-3.84 (m, 0.2H, H-4’), 3.98-4.03 (m, 0.8H, H-4’), 4.23‑4.27 (m, 0.2H, H-2’), 4.29-4.34 (m, 0.8H, H-2’), 4.50-4.56 (m, 0.2H, H-3’), 4.59-4.69 (m, 0.8H, H-3’), 4.96 (app t, J = 5.2 Hz, 0.2H, OH‑5’), 5.24 (app t, J = 5.2 Hz, 0.8H, OH‑5’), 5.70 (d, J_5,6_ = 8.1 Hz, 1H, H-5), 5.77 (d, J_1’,2’_ = 3.8 Hz, 0.2H, H‑1’), 5.81 (d, J_1’,2’_ = 3.8 Hz, 0.8H, H-1’), 6.00 (d, J_2’,OH-2’_ = 4.9 Hz, 0.2H, OH-2’), 6.32 (d, J_2’,OH-2’_ = 4.9 Hz, 0.8H, OH-2’), 6.74-6.81 (m, 1H, H^Ar^), 6.84-6.93 (m, 2H, H^Ar^), 7.71-7.83 (m, 1H, H^Ar^), 7.99 (d, J_5,6_ = 8.1 Hz, 1H, H-6), 8.59 (d, J_3’,NH-3’_ = 8.6 Hz, 1H, NH-3’), 9.50 (br s, 0.2H, NH^Sq^), 9.53 (br s, 0.8H, NH^Sq^), 10.14 (br s, 1H, OH‑Ar), 10.31 (br s, 0.2H, NH^U^), 11.38 (br s, 0.8H, NH^U^) ppm.

^13^C NMR (151 MHz, DMSO-d_6_): *δ* = 54.7 (C-3’), 55.8 (C-3’), 60.2 (C-5’), 61.1 (C-5’), 70.9 (C-2’), 73.6 (C‑2’), 81.9 (C-4’), 83.5 (C-4’), 88.9 (C-1’), 89.5 (C-1’), 101.7 (C-5), 115.0 (C-Ar), 119.3 (C-Ar), 120.05 (C-Ar), 120.12 (C-Ar), 123.6 (C-Ar), 123.7 (C-Ar), 126.9 (C-Ar), 127.0 (C-Ar), 140.4 (C-6), 146.8 (C-Ar), 150.6 (C‑2), 163.2 (C-4), 164.0 (C-Sq2), 164.2 (C-Sq2), 168.9 (C-Sq1), 169.1 (C-Sq1), 180.5 (C-Sq3), 180.6 (C‑Sq3), 184.0 (C-Sq4) ppm.

HRMS (ESI^-^): *m/z* calc. 429.1052 [M-H]^-^, found: 429.1059

**3'-*N*-(2-(*N-*Methyl)hydroxylamino-3,4-dioxocyclobut-1-en-1-yl)amino-3'-deoxyuridine (26)**

3'-*N*-(2-Ethoxy-3,4-dioxocyclobut-1-en-1-yl)amino-3'-deoxyuridine (**23**) (41 mg, 112 μmol) was dissolved in EtOH (2 mL). *N*-Methylhydroxylamine hydrochloride (14 mg, 169 μmol) and triethylamine (69 μL, 494 μmol) were added. The reaction mixture was stirred at room temperature for 2 hours. TLC analysis (CH_2_Cl_2_‑MeOH, 90:10) after this time showed complete consumption of starting material (R_f_ = 0.3) and formation of product (R_f_ = 0.1). The solvent was removed under reduced pressure and the residue was purified by flash chromatography (CH_2_Cl_2_‑MeOH, 90:10, then 87:13) to provide the product **26** as a white crystalline solid (21 mg, 51%); mp 200-215 °C (decomp).

ν_max_/cm^-1^ (neat) 3448, 3238, 3045, 2926, 2797, 1801, 1683, 1667, 1557, 1451, 1411, 1380, 1263, 1252, 1226, 1155, 1096, 1062, 1025, 892, 844, 781, 675, 628.

^1^H NMR (600 MHz, DMSO-d_6_): *δ* = 3.37 (s, 3H, CH_3_), 3.57-3.63 (m, 1H, H-5’a), 3.70 (d, J_5’a,5’b_ = 11.6 Hz, 1H, H-5’b), 4.12-4.14 (m, 1H, H-4’), 4.15-4.18 (m, 1H, H-2’), 4.56-4.61 (m, 1H, H-3’), 5.16 (br s, 1H, OH‑5’), 5.65 (d, J_5,6_ = 8.0 Hz, 1H, H-5), 5.83 (d, J = 3.6 Hz, 1H, H-1’), 6.06 (br s, 1H, OH-2’), 7.32 (br s, 1H, NH-3’), 7.93 (d, J_5,6_ = 8.0 Hz, 1H, H-6), 10.76 (br s, 1H, N-OH), 11.35 (br s, 1H, NH^U^) ppm.

^13^C NMR (151 MHz, DMSO-d_6_): *δ* = 40.9 (CH_3_), 54.3 (C-3’), 60.3 (C-5’), 73.7 (C-2’), 82.4 (C-4’), 88.9 (C‑1’), 101.7 (C-5), 140.6 (C-6), 150.6 (C-2), 163.2 (C-4), 165.9 (C-Sq2), 166.8 (C-Sq1), 178.8 (C-Sq), 179.9 (C‑Sq) ppm.

HRMS (ESI^+^): *m/z* calc. 391.0860 [M+Na]^+^, found: 391.0862

**3'-*N*-(2-Hydroxy-3,4-dioxocyclobut-1-en-1-yl)amino-3'-deoxyuridine (27)**

3'-*N*-(2-Ethoxy-3,4-dioxocyclobut-1-en-1-yl)amino-3'-deoxyuridine (**23**) (36 mg, 98 μmol) was dissolved in EtOH (0.96 mL) and water (0.24 mL). NaOH (11 mg, 285 μmol) was added and the reaction mixture was stirred at room temperature for 3 hours. TLC analysis (EtOAc‑MeOH, 85:15) after this time showed consumption of starting material (R_f_ = 0.6) and formation of product (R_f_ = 0.0). The reaction mixture was eluted through Dialon WT01S ion-exchange resin (H form) and then eluted through Dialon WT01S ion exchange resin (Na form). The solvent was removed under reduced pressure, and the residue was purified by flash chromatography (water-*i*PrOH-EtOAc, 3:57:40) to obtain the product **27** as a white crystalline solid (16 mg, 40%); mp 188-194 °C (decomp).

ν_max_/cm^-1^ (neat) 3260, 2982, 1631, 1509, 1439, 1353, 1294, 1223, 1132, 1094, 1029, 988, 819, 792, 741, 701, 653.

^1^H NMR (600 MHz, D_2_O): *δ* = 3.85 (dd, J_5’a,5’b_ = 13.2 Hz, J_4’,5’a_ = 4.1 Hz, 1H, H-5’a), 3.98 (dd, J_5’a,5’b_ = 13.2 Hz, J_4’,5’b_ = 2.1 Hz, 1H, H-5’b), 4.18-4.22 (m, 1H, H-4’), 4.45 (dd, J_2’,3’_ = 5.7 Hz, J_1’,2’_ = 1.8 Hz, 1H, H‑2’), 4.63 (dd, J_3’,4’_ = 9.1 Hz, J_2’,3’_ = 5.7 Hz, 1H, H-3’), 5.83 (d, J_5,6_ = 7.7 Hz, 1H, H-5), 5.94 (d, J_1’,2’_ = 1.8 Hz, 1H, H‑1’), 7.82 (d, J_5,6_ = 7.7 Hz, 1H, H-6) ppm.

^13^C NMR (151 MHz, D_2_O): *δ* = 53.6 (C-3’), 60.0 (C-5’), 74.5 (C-2’), 82.1 (C-4’), 91.4 (C-1’), 102.4 (C-5), 140.4 (C-6), 159.3 (C-2), 177.1 (C-4), 180.9 (C-Sq1), 188.4 (C-Sq3), 195.7 (C-Sq2, C-Sq4) ppm.

HRMS (ESI^+^): *m/z* calc. 384.0414 [M+Na]^+^, found: 384.0417

## Thiosquaramides

**5'-*N*-(2-Cyclopentyloxy-3,4-dithionecyclobut-1-en-1-yl)amino-5'-deoxythymidine (30)**

5’-Amino-5’-deoxythymidine (**28**) (100 mg, 440 μmol) was dissolved in anhydrous DMF (1.2 mL). Dicyclopentyl dithiosquarate (**29**) (125 mg, 440 μmol) was added and the reaction mixture was stirred under argon at room temperature for 4 hours. TLC analysis (water-*i*PrOH-EtOAc, 1:2:2) after this time showed complete consumption of starting material (R_r_ = 0.0) and formation of product (R_f_ = 0.4). The solvent was removed under reduced pressure, and the residue was co‑evaporated with toluene (50 mL) to provide the product as a yellow amorphous solid (190 mg, 99%). The product was used in the next step without further purification.

ν_max_/cm^-1^ (neat) 3236, 2958, 2518, 2160, 2024, 1659, 1577, 1465, 1404, 1263, 1230, 1046, 959, 883, 764, 692.

^1^H NMR (400 MHz, DMSO-d_6_) δ = 1.46 – 1.72 (m, 9H, 4 x CH_2_^Cp^, CH^Cp^), 1.78 (d, J_CH3,6_ = 1.0 Hz, 3H, CH_3_^T^), 2.10 (ddd, J_2’a,2’b_ = 13.6 Hz, J_1’,2’a_ = 6.6 Hz, J_2’a,3’_ = 3.6 Hz, 1H, H‑2’a), 2.23 (dt, J = 6.6 Hz, J = 3.6 Hz, 1H, H-2’b), 3.86 – 3.93 (m, 1H, H-4’), 4.12 (ddd, J_5’a,5’b_ = 13.3 Hz, J_NH,5’a_ = 8.4 Hz, J_4’,5’a_ = 5.4 Hz, 1H, H-5’a), 4.21 (app dt, J = 6.6 Hz, J = 3.6 Hz, 1H, H-3’), 4.62 (ddd, J_5’a,5’b_ = 13.3 Hz, J_NH,5’b_ = 7.2 Hz, J_4’,5’b_ = 4.1 Hz, 1H, H‑5’b), 6.22 (app t, J = 6.6 Hz, 1H, H-1’), 7.46 (app d, J = 1.0 Hz, 1H, H-6), 8.68 (dd, J_NH,5’a_ = 8.4 Hz, J_NH,5’b_ = 7.2 Hz, 1H, NH‑5’), 11.32 (s, 1H, NH^T^) ppm.

^13^C NMR (100 MHz, DMSO-d_6_) δ = 12.1 (CH_3_^T^), 22.9 (CH_2_^Cp^), 23.1 (CH_2_^Cp^), 23.25 (CH_2_^Cp^), 23.30 (CH_2_^Cp^), 30.1 (CH^Cp^), 38.2 (C-2’), 45.0 (C-5’), 70.8 (C-3’), 83.8 (C-1’), 84.5 (C-4’), 110.1 (C-5), 136.1 (C-6), 150.5 (C-2), 163.6 (C-4), 170.5 (C-Sq), 204.2 (C-Sq) ppm.

HRMS (ESI^-^): *m/z* calc. 436.1006 [M-H]^-^, found: 436.1017

**5'-*N*-(2-Diethylamino-3,4-dithionecyclobut-1-en-1-yl)amino-5'-deoxythymidine (31)**

5'-*N*-(2-Cyclopentyloxy-3,4-dithionecyclobut-1-en-1-yl)amino-5'-deoxythymidine (**30**) (43 mg, 98 μmol) was dissolved in anhydrous DMF (1 mL). Diethylamine (99 μL, 957 μmol) was added and the reaction mixture was stirred under argon at room temperature for 23.5 hours. The solvent was removed under reduced pressure and the residue was purified by flash chromatography (EtOAc‑*i*PrOH, 75:25). Remaining impurities were removed through further purification by flash chromatography (CH_2_Cl_2_-MeOH, 95:5 then 90:10, then EtOAc-*i*PrOH, 75:25) to provide the product **31** as a yellow amorphous solid (11 mg, 27%).

Note: compound **31** exhibits rotamers in NMR spectroscopy.

ν_max_/cm^-1^ (neat) 3229, 3053, 2969, 2926, 1686, 1561, 1461, 1432, 1379, 1320, 1263, 1234, 1199, 1178, 1127, 1075, 1022, 951, 804, 764, 613.

^1^H NMR (400 MHz, DMSO-d_6_): *δ* = 1.19-1.27 (m, 6H, 2 x CH_3_^Et^), 1.78 (s, 3H, CH_3_^T^), 2.05-2.14 (m, 2H, H‑2’a, H-2’b), 3.51-3.60 (m, 2H, CH_2_^Et^), 3.87-3.91 (m, 0.2H, H-4’), 4.00 (td, J_4’,5’_ = 7.0 Hz, J_3’,4’_ = 3.0 Hz, 0.8H, H‑4’), 4.11-4.32 (m, 4H, H-3’, H-5’a, CH_2_^Et^), 4.53-4.62 (m, 0.2H, H-5’b), 4.67-4.77 (m, 0.8H, H-5’b), 5.38 (d, J_3’,OH-3’_ = 4.3 Hz, 0.8H, OH-3’), 5.44 (d, J_3’,OH-3’_ = 4.3 Hz, 0.2H, OH-3’), 6.16 (app t, J = 7.0 Hz, 0.8H, H-1’), 6.21 (app t, J = 7.0 Hz, 0.2H, H-1’), 7.44 (s, 0.8H, H-6), 7.47 (s, 0.2H, H-6), 8.79 (br s, 0.8H, NH-5’), 8.90 (br s, 0.2H, NH-5’), 11.32 (br s, 1H, NH^T^) ppm.

^13^C NMR (151 MHz, DMSO-d_6_): *δ* = 12.1 (CH_3_^T^), 15.0 (CH_3_^Et^), 15.3 (CH_3_^Et^), 38.4 (C-2’), 43.7 (CH_2_^Et^), 45.0 (C-5’), 45.2 (CH_2_^Et^), 70.7 (C-3’), 84.0 (C-1’), 84.5 (C-4’), 109.8 (C-5), 135.8 (C-6), 150.4 (C-2), 163.7 (C-4), 169.1 (C-Sq1), 169.9 (C-Sq2), 201.7 (C-Sq4), 204.9 (C-Sq3) ppm.

HRMS (APCI^-^): *m/z* calc. 423.1166 [M-H]^-^, found: 423.1162

**5'-*O*-(4,4'-Dimethoxytrityl)-3'-azido-3'-deoxythymidine**^5^ **(33)**

3’-Azido-3’-deoxythymidine **32** (2.67 g, 10.0 mmol) was suspended in anhydrous CH_2_Cl_2_ (35 mL). DIPEA (5.23 mL, 30.0 mmol) was added and the reaction mixture was stirred under argon at room temperature. 4,4’-Dimethoxytrityl chloride (3.73 g, 11.0 mmol) was added in 4 equal portions over 10 minutes and the reaction mixture was stirred under argon at room temperature for a further 1 hour. TLC analysis (CH_2_Cl_2_-MeOH, 95:5) after this time showed consumption of the starting material (R_f_ = 0.3) and the formation of product (R_f_ = 0.7). The reaction mixture was diluted with CH_2_Cl_2_ (65 mL) and washed with water (50 mL) and brine (50 mL). The organic layer was dried over Na_2_SO_4_ and filtered. The solvent was removed under reduced pressure, and the residue was purified by flash chromatography (CH_2_Cl_2_-MeOH, 100:0 - 96:4) to provide the product **33** as a white foam (3.02 g, 53%).

^1^H NMR (400 MHz, DMSO-d_6_): *δ* = 1.54 (d, J_CH3,6_ = 1.0 Hz, 3H, CH_3_^T^), 2.31-2.40 (m, 1H, H-2’a), 2.45-2.54 (m, 1H, H-2’b), 3.24 (d, J_3’,4’_ = 3.7 Hz, 1H, H-3’), 3.28-3.37 (m, 1H, H-5’a), 3.73 (s, 6H, 2 x OCH_3_), 3.88 (app dt, J = 5.9 Hz, J = 3.7 Hz, 1H, H-4’), 4.56 (dd, J_5’a,5’b_ = 13.3 Hz, J_4’,5’b_ = 5.9 Hz, 1H, H-5’b), 6.13 (app t, J = 6.4 Hz, 1H, H-1’), 6.82-6.95 (m, 4H, H^Ar^), 7.16-7.36 (m, 7H, H^Ar^), 7.34-7.44 (m, 2H, H^Ar^), 7.52 (app d, J = 1.0 Hz, 1H, H-6), 11.37 (s, 1H, NH^T^) ppm.

HRMS (ESI^+^): *m/z* calc. 592.2167 [M+Na]^+^, found: 592.2168

**5'-*O*-(4,4'-Dimethoxytrityl)-3'-amino-3'-deoxythymidine**^6^ **(34)**

5'-*O*-(4,4'-Dimethoxytrityl)-3'-azido-3'-deoxythymidine (**33**) (300 mg, 0.53 mmol) was suspended in *i*PrOH (10 mL). NaBH_4_ (107 mg, 2.83 mmol) was added and the reaction mixture was heated to reflux for 16 hours. TLC analysis (CH_2_Cl_2_-MeOH, 90:10) after this time indicated complete consumption of the starting material (R_f_ = 0.8) and the formation of product (R_f_ = 0.3). The homogeneous reaction mixture was cooled to room temperature and the resulting heterogeneous mixture was poured onto CH_2_Cl_2_ (20 mL). The solution was washed with half-saturated aqueous NaCl solution (10 mL). The layers were separated, and the aqueous layer was extracted with CH_2_Cl_2_ (10 mL). The combined organic layers were washed with brine (10 mL), dried over Na_2_SO_4_, and filtered. The solvent was removed under reduced pressure to provide the product **34** as a white foam (276 mg, 97%).

^1^H NMR (400 MHz, DMSO-d_6_): *δ* = 1.47 (d, J_CH3,6_ = 1.0 Hz, 3H, CH_3_^T^), 2.03 (app dt, J = 13.0 Hz, J = 6.7 Hz, 1H, H-2’a), 2.20 (ddd, J_2’a,2’b_ = 13.0 Hz, J_2’b,3’_ = 7.2 Hz, J_1’,2’b_ = 5.5 Hz, 1H, H-2’b), 3.17-3.24 (m, 1H, H-3’), 3.50 (dd, J_5’a,5’b_ = 13.2 Hz, J_4’,5’a_ = 6.8 Hz, 1H, H-5’a), 3.68 (app dt, J = 6.8 Hz, J = 3.7 Hz, 1H, H-4’), 3.73 (s, 6H, 2 x OCH_3_), 3.73-3.81 (m, 1H, H-5’b), 6.14 (dd, J_1’,2’a_ = 6.7 Hz, J_1’,2’b_ = 5.5 Hz, 1H, H-1’), 6.80-6.97 (m, 4H, H^Ar^), 7.16-7.36 (m, 7H, H^Ar^), 7.35-7.45 (m, 2H, H^Ar^), 7.50 (app d, J = 1.0 Hz, 1H, H-6) ppm.

HRMS (ESI^+^): *m/z* calc. 542.2369 [M-H]^-^, found: 542.2363

**5'-*O*-(4,4'-Dimethoxytrityl)-3'-(2-cyclopentoxy-3,4-dithionecyclobuten-1-yl)-amino-3'-deoxythymidine (35)**

Dicyclopentyl dithiosquarate **29** (286 mg, 1.01 mmol) was dissolved in anhydrous CH_2_Cl_2_ (3 mL). The reaction mixture was cooled to 0 °C and stirred under argon. Freshly prepared 5'‑*O*‑(4,4'‑dimethoxytrityl)-3'-amino-3'-deoxythymidine (**34**) (500 mg, 0.92 mmol) was added and the reaction mixture was stirred for 15 minutes at 0 °C and then for 4 hours at room temperature. After this time, TLC analysis (CH_2_Cl_2_‑MeOH, 90:10) showed complete consumption of the starting material (R_f_ = 0.3) and formation of product (R_f_ = 0.7). The solvent was removed under reduced pressure, and the residue was purified by flash chromatography (CH_2_Cl_2_-MeOH, 97:3) to provide the product **35** as a yellow foam (544 mg, 80%). This material was used immediately in the next step.

Notes: in the ^1^H NMR spectrum the signal for H-2’ is obscured by the residual protonated DMSO-d_6_ peak and the signal for H-5’b is obscured by H_2_O peak. Compound **35** exhibits four rotamers in NMR spectroscopy

ν_max_/cm^-1^ (neat) 3160, 2953, 1676, 1607, 1508, 1444, 1362, 1286, 1248, 1175, 1112, 1031, 960.

^1^H NMR (600 MHz, DMSO-d_6_) δ = 1.48-1.69 (m, 6.5H, CH_2_^Cp^, CH_3_^T^), 1.71-2.08 (m, 4.5H, CH_2_^Cp^, CH_3_^T^), 2.34-2.40 (m, 0.15H, H-2’a), 2.44 (app dt, J = 13.6 Hz, J = 6.3 Hz, 0.35H, H-2’a), 2.47-2.55 (m, 1.5 H, H‑2’a, H-2’b), 3.25 (dd, J_5’a,5’b_ = 10.7 Hz, J_4’,5’a_ = 3.5 Hz, 0.5H, H-5’a), 3.27-3.28 (m, 0.5H, H-5’a), 3.34‑3.38 (m, 0.5H, H‑5’b), 3.72 (s, 3H, OCH_3_), 3.73 (s, 3H, OCH_3_), 4.01-4.05 (m, 0.15H, H-4’), 4.05-4.11 (m, 0.85H, H-4’), 4.41-4.50 (m, 0.7H, H-3’), 5.77-5.85 (m, 0.3H, H-3’), 6.22 (app t, J = 6.3 Hz, 0.5H H‑1’), 6.24 (app t, J = 6.3 Hz, 0.1H, H-1’), 6.27-6.38 (m, 1.3H, CH^Cp^, H-1’), 6.36-6.40 (m, 0.1H, CH^Cp^), 6.82-6.89 (m, 4H, H^Ar^), 7.06-7.10 (m, 0.5H, H^Ar^), 7.17-7.25 (m, 4.5H, H^Ar^), 7.28 (app t, J = 7.6 Hz, 2H, H^Ar^), 7.35 (app t, J = 8.3 Hz, 2H, H^Ar^), 7.49 (s, 0.5H, H-6), 7.57 (s, 0.35H, H-6), 7.74 (s, 0.05H, H-6), 7.79 (s, 0.1H, H-6), 9.98 (d, J_3’,NH-3’_ = 8.5 Hz, 0.4H, NH-3’), 10.06 (d, J_3’,NH-3’_ = 8.5 Hz, 0.5H, NH‑3’), 10.08 (d, J_3’,NH-3’_ = 8.5 Hz, 0.1H, NH-3’), 11.32 (s, 0.05H, NH^T^), 11.33 (s, 0.1H, NH^T^), 11.35 (s, 0.05H, NH^T^), 11.38 (s, 0.8H, NH^T^) ppm.

^13^C NMR (150 MHz, DMSO-d_6_): δ = 11.9 (CH_3_^T^), 11.9 (CH_3_^T^), 12.2 (CH_3_^T^), 23.1 (CH_2_^Cp^), 23.2 (CH_2_^Cp^), 23.30 (CH_2_^Cp^), 23.32 (CH_2_^Cp^), 33.4 (CH_2_^Cp^), 33.7 (CH_2_^Cp^), 33.8 (CH_2_^Cp^), 33.9 (CH_2_^Cp^), 37.3 (C-2’), 37.7 (C-2’), 53.4 (C-3’), 54.4 (C-3’), 55.0 (OCH_3_), 55.1 (OCH_3_), 55.3 (C‑3’), 62.6 (C-5’), 63.1 (C-5’), 81.6 (C-4’), 82.0 (C-4’), 83.4 (C-1’), 83.8 (C-4’), 84.0 (C-1’), 86.0 (qC^DMT^), 86.1 (qC^DMT^), 87.41 (CH^Cp^), 87.43 (CH^Cp^), 109.6 (C-5), 109.8 (C-5), 112.8 (Ar), 113.1 (Ar), 113.17 (Ar), 113.21 (Ar), 126.4 (Ar), 126.8 (Ar), 127.4 (Ar), 127.5 (Ar), 127.6 (Ar), 127.7 (Ar), 127.8 (Ar), 127.9 (Ar), 128.9 (Ar), 129.6 (Ar), 129.7 (Ar), 135.14 (Ar), 135.18 (Ar), 135.25 (Ar), 135.8 (C-6), 136.0 (C-6), 140.2 (Ar), 144.5 (Ar), 144.6 (Ar), 148.3 (Ar), 150.2 (C-2), 150.4 (C-2), 150.5 (C-2), 157.8 (Ar), 158.1 (Ar), 158.2 (Ar), 163.6 (C-4), 163.7 (C-4), 172.7 (C-Sq2), 174.8 (C-Sq2), 182.1 (C-Sq1), 182.4 (C-Sq1), 182.6 (C-Sq1), 206.01 (C-Sq3), 206.04 (C-Sq3), 216.9 (C-Sq4), 217.5 (C-Sq4) ppm.

HRMS (ESI^+^): *m/z* calc. 762.2278 [M + Na]^+^, found: 762.2257

**5'-*O*-(4,4'-Dimethoxytrityl)-3'-*N*-(2-diethylamino-3,4-dithionecyclobut-1-en-1-yl)amino-3'-deoxythymidine (36)**

5'-*O*-(4,4'-Dimethoxytrityl)-3'-*N*-(2-cyclopentyloxy-3,4-dithionecyclobut-1-en-1-yl)amino-3'-deoxythymidine (**35**) (41 mg, 56 μmol) was dissolved in CH_2_Cl_2_ (2 mL). Diethylamine (87 μL, 841 μmol) was added and the reaction mixture was stirred under argon at room temperature for 2.5 hours. TLC analysis (toluene-acetone, 65:35) after this time showed complete consumption of starting material (R_f_ = 0.5) and formation of product (R_f_ = 0.3). The solvent was removed under reduced pressure and the residue was purified by flash chromatography (toluene-acetone, 65:35) to provide the product **36** as an orange amorphous solid (26 mg, 64%).

ν_max_/cm^-1^ (neat) 3200, 2943, 1800, 1665, 1568, 1509, 1441, 1331, 1249, 1203, 1176, 1076, 1026, 826, 728, 698.

^1^H NMR (600 MHz, acetone-d_6_): *δ* = 1.22-1.34 (m, 6H, 2 x CH_3_^Et^), 1.65 (d, J_6,CH3_ = 0.7 Hz, 3H, CH_3_^T^), 2.62‑2.68 (m, 2H, H-2’a, H-2’b), 3.45 (dd, J_5’a,5’b_ = 10.6 Hz, J_4’,5’a_ = 3.3 Hz, 1H, H-5’a), 3.49 (dd, J_5’a,5’b_ = 10.6 Hz, J_4’,5’b_ = 3.3 Hz, 1H, H-5’b), 3.56 (br s, 1H, CH_2_^Et^), 3.65 (br s, 1H, CH_2_^Et^), 3.772 (s, 3H, OCH_3_), 3.774 (s, 3H, OCH_3_), 4.21 (app dt, J = 6.7 Hz, J = 3.3 Hz, 1H, H-4’), 4.30 (br s, 1H, CH_2_^Et^), 4.45 (br s, 1H, CH_2_^Et^), 6.32 (app t, J = 6.4 Hz, 1H, H-1’), 6.69 (m, 1H, H-3’), 6.82-6.89 (m, 4H, H^Ar^), 7.19-7.25 (m, 2H, H^Ar^), 7.27-7.32 (m, 2H, H^Ar^), 7.34-7.39 (m, 3H, H^Ar^), 7.47-7.54 (m, 2H, H^Ar^), 7.68 (app d, J = 0.7 Hz, 1H, H‑6), 7.75 (d, J_3’,NH-3’_ = 9.1 Hz, 1H, NH-3’), 9.97 (br s, 1H, NH^T^) ppm.

^13^C NMR (151 MHz, acetone-d_6_): *δ* = 12.5 (CH_3_^T^), 15.5 (CH_3_^Et^), 15.8 (CH_3_^Et^), 40.3 (C-2’), 44.9 (CH_2_^Et^), 46.2 (CH_2_^Et^), 54.6 (C-3’), 55.5 (OCH_3_), 63.3 (C-5’), 82.9 (C-4’), 83.9 (C-1’), 87.4 (qC^DMT^), 111.5 (C-5), 113.93 (Ar), 113.94 (Ar), 127.6 (Ar), 128.7 (Ar), 129.1 (Ar), 131.11 (Ar), 131.13 (Ar), 136.5 (C-6), 145.9 (Ar), 151.4 (C-2), 159.66 (Ar), 159.68 (Ar), 164.2 (C-4), 170.3 (C-Sq), 171.1 (C-Sq), 204.4 (C-Sq), 209.2 (C-Sq) ppm.

HRMS (APCI^-^): *m/z* calc. 725.2473 [M-H]^-^, found: 725.2484

**3'-*N*-(2-Diethylamino-3,4-dithionecyclobut-1-en-1-yl)amino-3'-deoxythymidine (37)**

5'-*O*-(4,4'-Dimethoxytrityl)-3'-*N*-(2-diethylamino-3,4-dithionecyclobut-1-en-1-yl)amino-3'-deoxythymidine (**36**) (56 mg, 77 μmol) was suspended in MeOH (1.5 mL). AcOH (1.5 mL) was added and the reaction mixture was stirred at room temperature for 3.5 hours. TLC analysis (toluene‑acetone, 1:1) after this time showed complete consumption of starting material (R_f_ = 0.5) and formation of product (R_f_ = 0.1). The solvent was removed under reduced pressure, co-evaporating with toluene. The residue was purified by flash chromatography (toluene‑acetone, 1:1 then 2:3) to provide the product as a yellow amorphous solid (25 mg, 76%).

ν_max_/cm^-1^ (neat) 3191, 3050, 2972, 2927, 1795, 1664, 1537, 1441, 1331, 1267, 1246, 1229, 1120, 1178, 1086, 1024, 962, 803, 753.

^1^H NMR (400 MHz, acetone-d_6_): *δ* = 1.29 (br s, 3H, CH_3_^Et^), 1.37 (br s, 3H, CH_3_^Et^), 1.85 (d, J_6,CH3_ = 1.1 Hz, 3H, CH_3_^T^), 2.57 (m, 1H, H-2’a), 2.65 (app dt, J = 14.0 Hz, J = 7.0 Hz, 1H, H-2’b), 3.69 (br s, 2H, CH_2_^Et^), 3.88 (ddd, J_5’a,5’b_ = 12.1 Hz, J_5’a,OH-5’_ = 5.7 Hz, J_4’,5’a_ = 2.6 Hz, 1H, H-5’a), 3.94 (ddd, J_5’a,5’b_ = 12.1 Hz, J_5’b,OH‑5’_ = 5.7 Hz, J_4’,5’b_ = 3.3 Hz, 1H, H-5’b), 4.15 (m, 1H, H-4’), 4.27 (app t, J = 5.7 Hz, 1H, OH-5’), 4.38 (br s, 2H, CH_2_^Et^), 6.27 (dd, J_1’,2’b_ = 7.0 Hz, J_1’,2’a_ = 5.2 Hz, 1H, H-1’), 6.38 (m, 1H, H-3’), 7.84 (d, J_3’,NH-3’_ = 9.0 Hz, 1H, NH-3’), 7.87 (app d, J = 1.1 Hz, 1H, H-6), 9.96 (br s, 1H, NH^T^) ppm.

^13^C NMR (151 MHz, acetone-d_6_): *δ* = 12.7 (CH_3_^T^), 15.6 (CH_3_^Et^), 15.8 (CH_3_^Et^), 40.1 (C-2’), 45.0 (CH_2_^Et^), 46.5 (CH_2_^Et^), 54.2 (C-3’), 61.5 (C-5’), 84.3 (C-1’), 85.6 (C-4’), 111.0 (C-5), 137.0 (C-6), 151.5 (C-2), 164.3 (C-4), 170.6 (C-Sq2), 171.0 (C-Sq1), 204.1 (C-Sq4), 208.7 (C-Sq3) ppm.

HRMS (APCI^-^): *m/z* calc. 423.1166 [M-H]^-^, found: 423.1174

***N*-(5'-Deoxythymidine-5'-yl)-*N*'-(5'-*O*-(4,4'-dimethoxytrityl)-3’-deoxythymidine-3'-yl)-1,2-diamino-3,4‑dithionecyclobut-1-ene (38)**

5'-*O*-(4,4'-Dimethoxytrityl)-3'-(2-cyclopentoxy-3,4-dithionecyclobuten-1-yl)-amino-3'-deoxythymidine (**35**) (510 mg, 0.69 mmol) was dissolved in anhydrous CH_2_Cl_2_ (5 mL) and the reaction mixture was cooled to 0 °C and stirred under argon. In a separate flask, under argon, freshly prepared 5’-amino-5’-deoxythymidine (**28)** (175 mg, 0.73 mmol) was dissolved in a mixture of anhydrous CH_2_Cl_2_ (9 mL) and anhydrous DMF (3 mL). The solution of amine **28** was added dropwise to the reaction mixture. The reaction mixture was stirred for 15 minutes at 0 °C and then for 1 hour at room temperature. TLC analysis (CH_2_Cl_2_-MeOH, 90:10) after this time showed complete consumption of the starting material (R_f_ = 0.7) and the formation of product (R_f_ = 0.2). The solvent was removed under reduced pressure. The residue was co-evaporated successively with toluene (3 × 5 mL), MeOH (5 mL) and CH_2_Cl_2_ (5 mL). Purification by flash chromatography (CH_2_Cl_2_-MeOH, 90:10) provided the product **38** as a yellow foam (470 mg, 76%).

Notes: in the ^1^H NMR spectrum the signal for ^5’T^H-2’a is obscured by the residual protonated DMSO‑d_6_ peak and the signal for ^5’T^H-5’ is obscured by the H_2_O peak. Compound **38** exhibits rotamers in NMR spectroscopy.

ν_max_/cm^-1^ (neat) 3184, 2925, 1685, 1608, 1561, 1508, 1466, 1228, 1175, 1088, 1064, 1029.

^1^H NMR (600 MHz, DMSO-d_6_): δ = 1.53 (s, 2.4H, ^5’T^CH_3_^T^), 1.58 (s, 0.6H, ^3’T^CH_3_^T^), 1.66 (s, 0.6H, ^5’T^CH_3_^T^), 1.79 (s, 2.4H, ^3’T^CH_3_^T^), 2.06-2.15 (m, 1.2H, ^3’T^H-2’a, ^3’T^H-2’b), 2.26 (app dt, J = 14.1 Hz, J = 7.2 Hz, 0.8H, ^3’T^H‑2’b), 2.29-2.42 (m, 0.4H, ^5’T^H-2’a, ^5’T^H-2’b), 2.46-2.52 (m, 0.8H, ^5’T^H-2’a), 2.54-2.63 (m, 0.8H, ^5’T^H‑2’b), 3.25 (dd, J_5’a,5’b_ = 10.4 Hz, J_4’,5’a_ = 2.7 Hz, 1H, ^5’T^H-5’a), 3.34-3.37 (m, 1H, ^5’T^H-5’b), 3.71 (s, 0.5H, OCH_3_), 3.72 (s, 0.5H, OCH_3_), 3.73 (s, 5H, OCH_3_), 3.74-3.78 (m, 0.4H, ^3’T^H-5’a, ^3’T^H-5’b), 3.79-3.83 (m, 0.2H, ^3’T^H-4’), 3.91 (app dt, J = 8.5 Hz, J = 4.0 Hz, 0.8H, ^3’T^H-4’), 4.04-4.12 (m, 1H, ^5’T^H-4’), 4.16 (ddd, J_5’a,5’b_ = 13.7 Hz, J_4’,5’a_ = 8.5 Hz, J_5’a,NH-5’_ = 6.9 Hz, 0.8H, ^3’T^H-5’a), 4.21-4.29 (m, 0.8H, ^3’T^H-3’), 4.26-4.31 (m, 0.2H, ^3’T^H-3’), 4.60 (ddd, J_5’a,5’b_ = 13.7 Hz, J_5’b,NH-5’_ = 6.9 Hz, J_4’,5’b_ = 4.0 Hz, 0.8H, ^3’T^H-5’b), 5.41 (d, J_3’,OH-3’_ = 4.7 Hz, 0.2H, ^3’T^OH-3’), 5.46 (d, J_3’,OH-3’_ = 4.7 Hz, 0.8H, ^3’T^OH-3’), 5.85-5.92 (m, 0.8H, ^5’T^H-3’), 6.15 (app t, J = 6.8 Hz, 0.2H, ^5’T^H-1’), 6.21- 6.28 (m, 1.6H, ^3’T^H-1’, ^5’T^H-1’), 6.29-6.39 (m, 0.4H, ^5’T^H-3’, ^3’T^H‑1’), 6.78-6.82 (m, 0.8H, H^Ar^), 6.82-6.87 (m, 3.2H, H^Ar^), 7.15-7.30 (m, 7H, H^Ar^), 7.33 (d, J = 7.4 Hz, 0.3H, H^Ar^), 7.37 (d, J = 7.4 Hz, 1.7H, H^Ar^), 7.41 (s, 0.15H, ^3’T^H-6), 7.50 (s, 0.8H, ^3’T^H-6), 7.55 (s, 0.8H, ^5’T^H‑6), 7.58 (s, 0.2H, ^5’T^H-6), 7.59 (s, 0.05H, ^3’T^H‑6), 8.52 (app t, J = 6.9 Hz, 0.8H, ^3’T^NH-5’), 8.62 (d, J_3’,NH-3’_ = 9.1 Hz, 0.15H, ^5’T^NH‑3’), 8.92 (app t, J = 6.9 Hz, 0.05H, ^3’T^NH-5’), 8.99 (d, J_3’,NH-3’_ = 9.1 Hz, 0.8H, ^5’T^NH-3’), 9.21 (d, J_3’,NH‑3’_ = 9.1 Hz, 0.05H, ^5’T^NH-3’), 9.29 (app t, J = 6.9 Hz, 0.15H, ^3’T^NH-5’), 11.24 (s, 0.05H, ^3’T^NH^T^), 11.27 (s, 0.15H, ^5’T^NH^T^), 11.33 (s, 0.8H, ^3’T^NH^T^), 11.38 (s, 0.15H, ^3’T^NH^T^), 11.40 (s, 0.8H, ^5’T^NH^T^), 11.44 (s, 0.05H, ^5’T^NH^T^) ppm.

^13^C NMR (150 MHz, DMSO-d_6_): δ = 11.9 (^5’T^CH_3_^T^), 12.0 (^3’T^CH_3_^T^), 12.1 (^3’T^CH_3_^T^), 12.7 (^5’T^CH_3_^T^), 38.2 (^3’T^C‑2’), 38.3 (^5’T^C-2’), 45.0 (^3’T^C-5’), 52.5 (^5’T^C-3’), 53.3 (^5’T^C-3’), 55.00 (OCH_3_), 55.02 (OCH_3_), 55.05 (OCH_3_), 55.06 (OCH_3_), 62.2 (^5’T^C-5’), 62.5 (^5’T^C-5’), 69.7 (^3’T^C-3’), 70.7 (^3’T^C-3’), 81.4 (^5’T^C-4’), 82.5 (^5’T^C‑4’), 82.7 (^5’T^C-1’), 82.8 (^3’T^C-1’), 83.1 (^5’T^C-1’), 83.78 (^3’T^C-4’), 83.85 (^3’T^C-1’), 84.5 (^3’T^C-4’), 85.8 (qC^DMT^), 86.1 (qC^DMT^), 109.9 (^5’T^C-5, ^3’T^C-5), 110.1 (^3’T^C-5), 110.3 (^5’T^C-5), 113.1 (CH^DMT^), 113.1 (CH^DMT^), 113.2 (CH^DMT^), 113.2 (CH^DMT^), 126.6 (CH^DMT^), 126.8 (CH^DMT^), 127.68 (CH^DMT^), 127.72 (CH^DMT^), 127.74 (CH^DMT^), 127.9 (CH^DMT^), 129.8 (CH^DMT^), 135.05 (^5’T^C-5), 135.09 (qC^DMT^), 135.16 (qC^DMT^), 135.21 (qC^DMT^), 135.6 (^5’T^C-6), 135.9 (^3’T^C-6), 136.2 (^3’T^C-6), 144.5 (qC^DMT^), 150.41 (^5’T^C‑2), 150.44 (^5’T^C-2), 150.48 (^3’T^C-2), 158.04 (qC^DMT^), 158.06 (qC^DMT^), 158.1 (qC^DMT^), 158.2 (qC^DMT^), 163.4 (^5’T^C-4), 163.6 (^5’T^C-4, ^3’T^C-4), 163.7 (^3’T^C-4), 169.3 (C-Sq2), 170.0 (C-Sq1), 170.4 (C-Sq2), 173.0 (C-Sq1), 203.0 (C-Sq4), 203.9 (C-Sq4), 204.9 (C-Sq3), 205.7 (C-Sq3) ppm.

HRMS (ESI^+^): *m/z* calc. 917.2609 [M + Na]^+^, found: 917.2613

***N*-(5'-Deoxythymidine-5'-yl)-*N*'-(3’-deoxythymidine-3'-yl)-1,2-diamino-3,4-dithionecyclobut-1-ene (39)**

*N*-(5'-Deoxythymidine-5'-yl)-*N*'-(5'-*O*-(4,4'-dimethoxytrityl)-3’-deoxythymidine-3'-yl)-1,2-diamino-3,4-dithionecyclobut-1-ene (**38**) (31 mg, 52 μmol) was suspended in MeOH (1 mL). AcOH (1 mL) was added and the reaction mixture was stirred at room temperature for 1.5 hours. TLC analysis (CH_2_Cl_2_‑MeOH, 85:15) after this time showed consumption of starting material (R_f_ = 0.7) and formation of product (R_f_ = 0.1). The reaction mixture was diluted with water (5 mL) and EtOAc (5 mL). The organic layer was separated and extracted with water (4 x 5 mL). The aqueous layers were combined, and the solvent was removed under reduced pressure. The residue was purified by flash chromatography (toluene‑acetone-MeOH, 4:5:1) to provide the product as a yellow amorphous solid (20 mg, 98%).

ν_max_/cm^-1^ (neat) 3223, 2969, 1682, 1560, 1461, 1432, 1320, 1263, 1234, 1199, 1075, 1023, 950, 804.

^1^H NMR (600 MHz, DMSO-d_6_): *δ* = 1.788 (s, 3H, CH_3_^T^), 1.794 (s, 3H, CH_3_^T^), 2.07-2.12 (m, 1H, ^3’T^H-2’a), 2.20-2.27 (m, 1H, ^3’T^H-2’b), 2.36-2.41 (m, 1H, ^5’T^H-2’a), 2.43-2.48 (m, 1H, ^5’T^H-2’b), 3.69 (br s, 2H, ^5’T^H‑5’a, ^5’T^H‑5’b), 3.90 (app dt, J = 7.7 Hz, J = 3.7 Hz, 1H, ^3’T^H-4’), 3.99-4.03 (m, 1H, ^5’T^H-4’), 4.20 (dd, J_5’a,5’b_ = 13.5 Hz, J_4’,5’a_ = 7.7 Hz, 1H, ^3’T^H-5’a), 4.25-4.28 (m, 1H, ^3’T^H-3’), 4.53 (dd, J_5’a,5’b_ = 13.5 Hz, J_4’,5’b_ = 3.7 Hz, 1H, ^3’T^H-5’b), 5.18 (br s, 1H, OH), 5.45 (br s, 1H, OH), 5.47-5.54 (m, 1H, ^5’T^H-3’), 6.20-6.27 (m, 2H, ^3’T^H-1’, ^5’T^H-1’), 7.51 (s, 1H, ^3’T^H-6), 7.75 (s, 1H, ^5’T^H-6), 8.87 (br s, 1H, NH^Sq^), 9.41 (br s, 1H, NH^Sq^), 11.32 (br s, 2H, ^3’T^NH^T^, ^5’T^NH^T^) ppm.

^13^C NMR (151 MHz, DMSO-d_6_): *δ* = 12.1 (^3’T^CH_3_^T^), 12.3 (^5’T^CH_3_^T^), 38.3 (^3’T^C-2’), 38.5 (^5’T^C-2’), 44.9 (^3’T^C‑5’), 53.7 (^5’T^C-3’), 60.7 (^5’T^C-5’), 70.7 (^3’T^C-3’), 83.3 (^5’T^C-1’), 83.8 (^3’T^C-1’), 84.5 (^3’T^C-4’), 85.0 (^5’T^C‑4’), 109.6 (^5’T^C-5), 110.1 (^3’T^C-5), 136.0 (^5’T^C-6), 136.1 (^3’T^C-6), 150.48 (^3’T^C-2), 150.49 (^5’T^C-2), 163.68 (^3’T^C-4), 163.71 (^5’T^C-4), 170.1 (C-Sq), 170.8 (C-Sq), 204.0 (C-Sq), 204.5 (C-Sq) ppm.

HRMS (APCI^-^): *m/z* calc. 591.1337 [M-H]^-^, found: 591.1335

# Gel electrophoresis assay

Truncated human SNM1A (698-1040) was stored as a 1.0 μM (0.04 mg/mL) solution in reaction buffer (20 mM HEPES‑KOH pH 7.5, 50 mM KCl, 10 mM MgCl_2_, 0.05% Triton-X, 0.1 mg/mL BSA, 5% glycerol, 0.5 mM). Thymidine (control) and modified nucleosides (1 mM in reaction mixture or as specified) were treated with SNM1A (25 fmol) in reaction buffer (10 μL) containing 4% DMSO on ice, and then incubated at 37 °C for 5 minutes. A solution of the oligonucleotide substrate (1 μL, 0.8 pmol/μL) was added and each reaction was incubated at 37 °C for a further 60 minutes. The reactions were stopped by addition of 2 μL of stop solution (95% formamide, 10 mM EDTA) followed by heating at 95 °C for 3 minutes. Oligonucleotides were separated on 15% acrylamide 6.5 M urea gels (2.9 g urea, 2.7 mL 40% acrylamide-bisacrylamide 25:1, 0.7 mL 10X TBE (0.9 M Tris, 0.9 M boric acid, 0.02 M EDTA pH 8.0), 1.3 mL H_2_O) in 1X TBE at 150 V for between 75 and 90 minutes, alongside bromophenol blue and xylene cyanol as markers for 8 nt and 28 nt respectively. The gels were imaged using a Typhoon FLA 9500.

# Real-time fluorescence assay

Real-time fluorescence assays were performed using a modified literature producedure^7,8^ utilising a 20-nucleotide ssDNA substrate of the following sequence: 5’‑A[FamT]AATTTGA[BHQT]CATCTATTAT‑3’ (Eurogentec). This oligonucleotide contained a fluorescein-conjugated thymine (FamT) as the second residue from the 5’-end, and a black-hole quencher moiety conjugated to a thymine residue (BHQT) eight nucleotides away. The oligonucleotide substrate was phosphorylated at the 5’-end using T4 polynucleotide kinase (New England Biolabs) according to the manufacturer’s protocol, and made up to 1.25 μM for addition to reactions. Nuclease reactions were carried out in black 384-well microplates in a total volume of 25 μL in nuclease buffer (20 mM HEPES‑KOH, pH 7.5, 50 mM KCl, 10 mM MgCl_2_, 0.5 mM DTT, 0.05% (v/v) Triton-X100, 5% (v/v) glycerol), with 125 nM oligonucleotide substrate and 2.5 nM SNM1A (698-1040). Reactions were carried out in the presence of twelve different concentrations (0–1000 μM) of each inhibitor. Six replicates of each reaction were performed. SNM1A was incubated with the inhibitor in the above nuclease buffer for 6.5 minutes at room temperature, before the reactions were started by the addition of the DNA substrate. The fluorescence spectra were measured at 37 °C using a SpectraMax i3x microplate reader (excitation at 495 nm, emission at 525 nm) with 7 readings taken every 30 seconds. The fluorescence intensity for each reaction was plotted against time, and the rate of increase was determined and normalised to the zero-inhibitor control. This was plotted against inhibitor concentration and the data were fitted using a “log[inhibitor] vs. normalised response” nonlinear regression algorithm on GraphPad Prism software (GraphPad Software, Inc., La Jolla, CA, USA) to calculate the IC_50_ values.


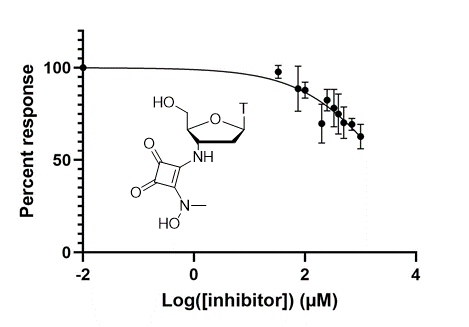


IC_50_ of *N*-hydroxysquaramide **8** – n.d.


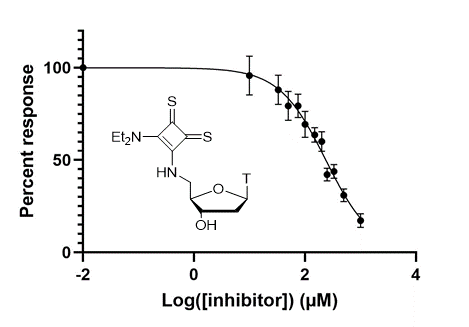
IC_50_ of 5’-thiosquaramide **31** – 238 µM (95% CI: 218‑259 µM)
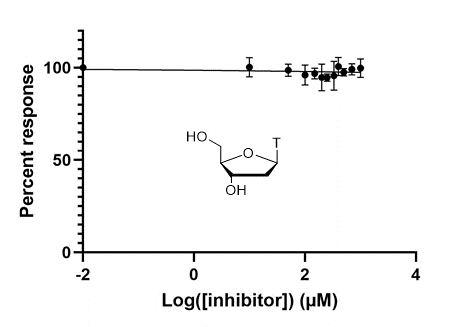
IC_50_ of thymidine – n.d.


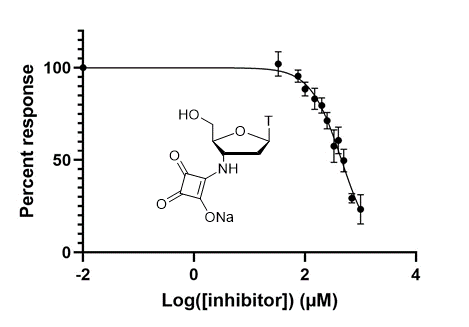


IC_50_ of squaric acid **9** – 456 µM (95% CI: 427.1‑487.7 µM)
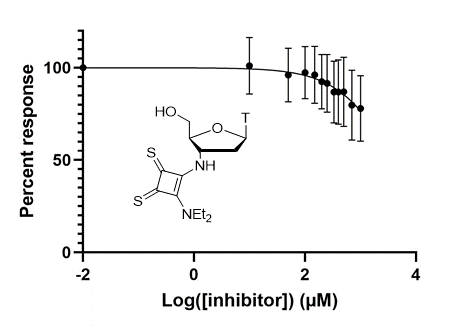
IC_50_ of 3’-thiosquaramide **37** – n.d.

Figure S1 - Dose-response curves obtained from real-time fluorescence assay. Error bars are ± one standard deviation.

# UV-vis titrations

UV-vis absorption spectra were recorded using a Varian Cary 50 spectrophotometer. A spectroscopic window of 600 – 200 nm was used for all spectra. Baseline correction from blank solvent was used for all spectra. Spectra were measured in a 1 cm quartz cuvette at room temperature.

Solutions of modified nucleosides in DMSO (spectroscopic grade), or in water in the case of squaric acid **9**, were made up to *ca.* 2.5 – 4 mM and diluted to *ca.* 10 μM in MeCN (spectroscopic grade) for the titrations. Solutions of zinc salts were made up in MeCN (spectroscopic grade) and aliquoted into the cuvette such that the volume in the cuvette did not increase by more than 10% in total during the titration. The data obtained were fitted to trial models of metal-ligand binding through global nonlinear regression analysis using the ReactLab Equilibria software (Jplus Consulting Pty Ltd., East Fremantle, WA, Australia).

High [Zn(ClO_4_)_2_]

Low [Zn(ClO_4_)_2_]

High [Zn(ClO_4_)_2_]

Low [Zn(ClO_4_)_2_]

Low [Zn(ClO_4_)_2_]

High [Zn(ClO_4_)_2_]

Figure S2 – UV-vis titration data for titrations of compounds **8**, **9** and **31** with Zn(ClO_4_)_2_.

# Parallel artificial membrane permeability assay (PAMPA)

The PAMPA assay was performed using a 96-well MultiScreen Filter Plate (Merck), with underdrain removed, as the donor plate, and a 96-well MultiScreen Transport Receiver Plate (Merck) as the acceptor plate, following the manufacturer’s protocol.^9^ Two known drug compounds, carbamazepine and furosemide were used as controls. Solutions of squaramides **8**, **9**, **26**, **31**, **37** and **39**, and carbamazepine and furosemide (500 µM) in PBS buffer (pH 7.4) containing 5% DMSO were prepared. PBS buffer (pH 7.4) containing 5% DMSO (300 µL) was added to each well of the acceptor plate. A solution of lecithin in dodecane (5 µL, 1% w/v) was added onto the filter at the bottom of donor well to form an artificial membrane. The drug solutions (150 μL, 500 µM) were immediately added to each well of the donor plate. The donor plate was then placed into the acceptor plate and incubated at room temperature for 16 h. After the incubation, a sample of each donor well solution (100 µL) and of each acceptor well solution (250 µL) were transferred into a UV-star 96‑well plate (Greiner Bio‑one). Solutions of each compound at the equilibrium concentration expected in both the donor and acceptor wells if the compounds are permeable were made up in PBS (pH 7.4) containing 5% DMSO and samples of the equilibrium solution (250 µL) were also transferred to the UV-star 96‑well plate (Greiner Bio-one). Absorbance was measured from 250-500 nm using a SpectraMax i3x microplate reader. Concentrations in each well were determined using calibration curves for each compound. The effective permeability P_e_ was calculated using the following equation:

$\boldsymbol{P}_{\boldsymbol{e}}\boldsymbol{=-ln(1-r)(}\frac{\boldsymbol{V}_{\boldsymbol{D}}\boldsymbol{V}_{\boldsymbol{A}}}{\left( \boldsymbol{V}_{\boldsymbol{D}}\boldsymbol{+}\boldsymbol{V}_{\boldsymbol{A}} \right)\boldsymbol{At}}\boldsymbol{)}$ where $\boldsymbol{r=}\frac{\boldsymbol{[drug]}_{\boldsymbol{acceptor}}}{\boldsymbol{[drug]}_{\boldsymbol{equilibrium}}}$

V_D_ = volume of donor well, 0.15 cm^3^ V_A_ = volume of acceptor well, 0.30 cm^3^

A = area of the filter, 0.3 cm^2^ t = incubation time, 57 600 s

Each compound was tested in this way in quadruplicate, and values reported are mean ± one standard deviation. The results obtained for carbamazepine and furosemide, logP_e_ = -5.3±0.07 and logP_e_ = ‑7.1±0.007 respectively, were in good agreement with previously reported values.^9^


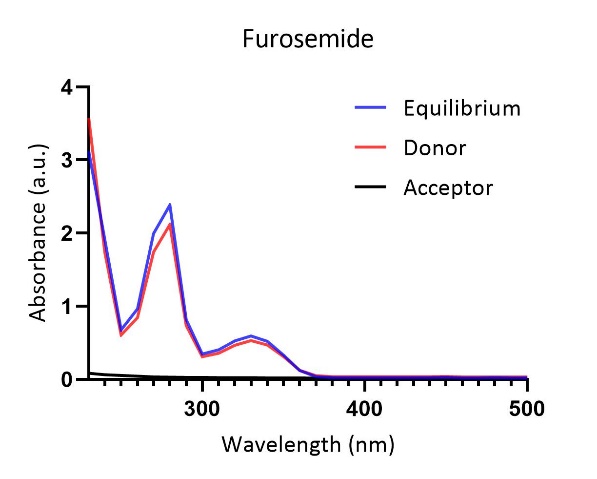

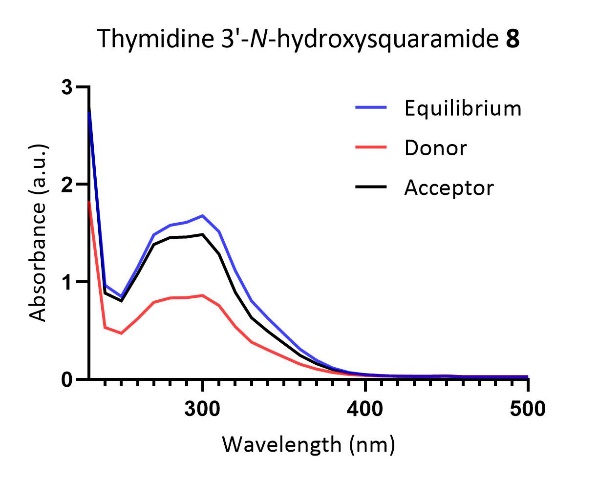

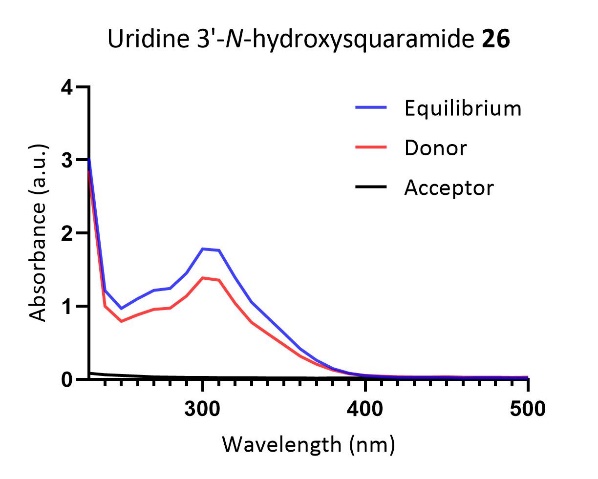

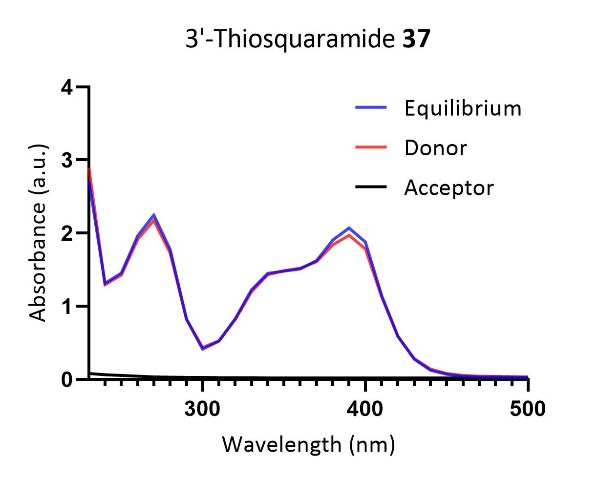


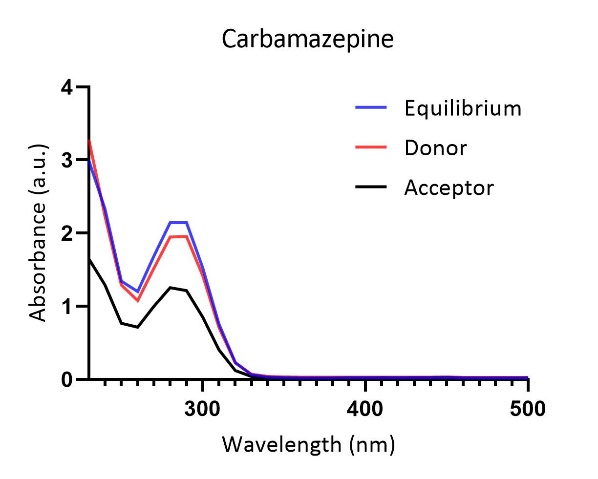

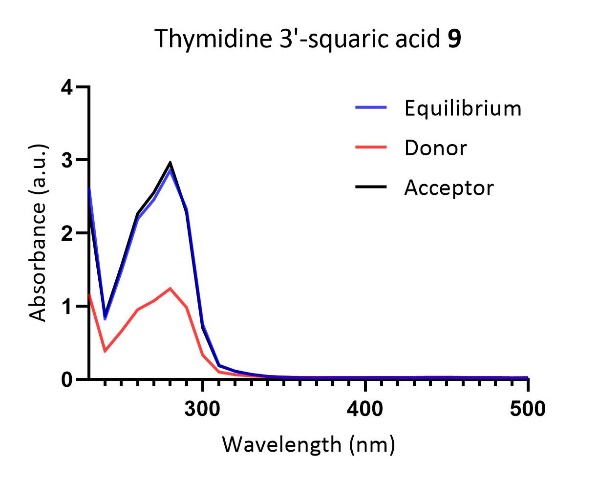

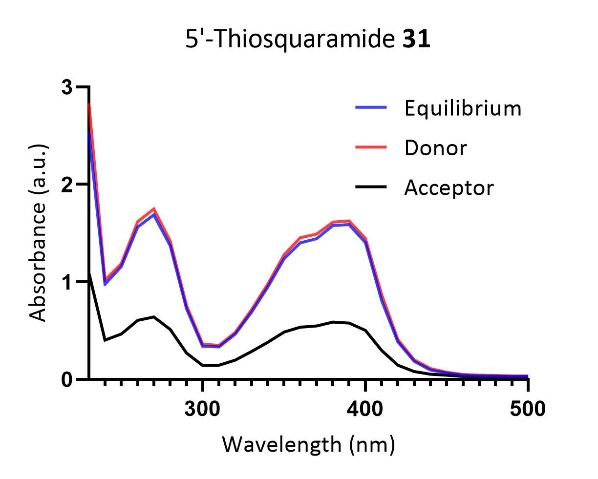

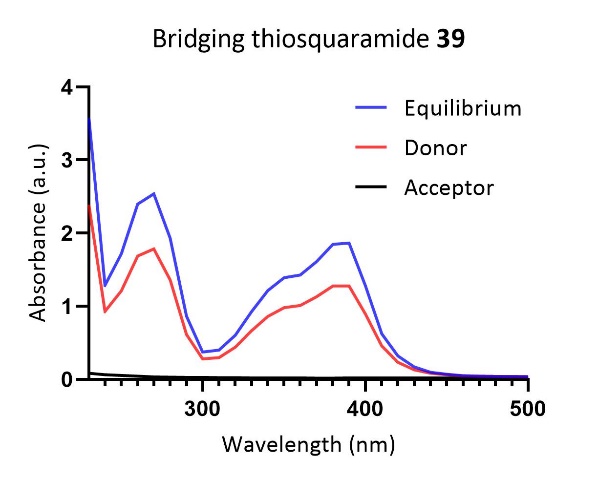


Figure S3 – PAMPA results: UV-vis absorption spectra of donor wells (100 µL), acceptor wells (250 µL), and equilibrium concentration solutions (250 µL). One of four replicates shown for each compound.

# ^1^H and ^13^C NMR Spectra of Novel Compounds

^1^H (600 MHz, DMSO-d_6_) and ^13^C (151 MHz, DMSO-d_6_) NMR spectra of **2**
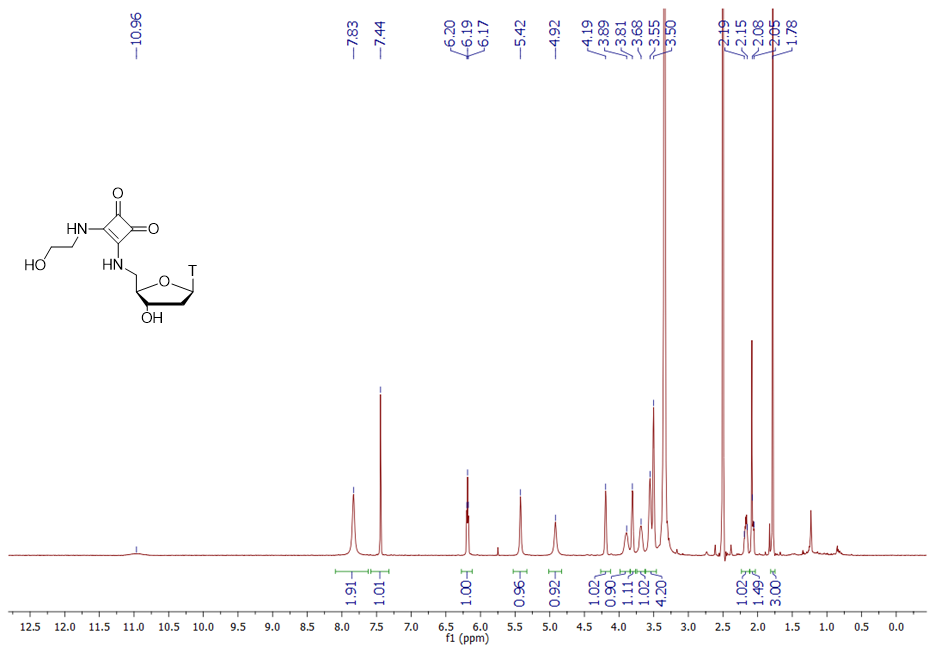


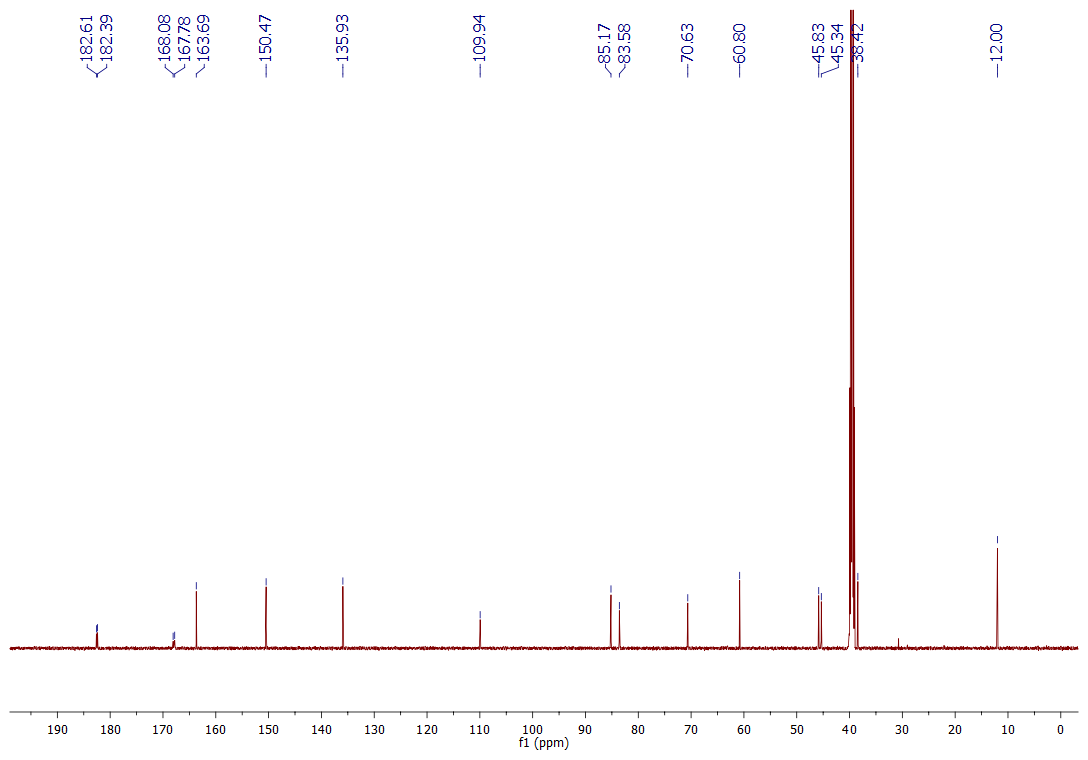


^1^H (600 MHz, DMSO-d_6_) and ^13^C (151 MHz, DMSO-d_6_) NMR spectra of **3**
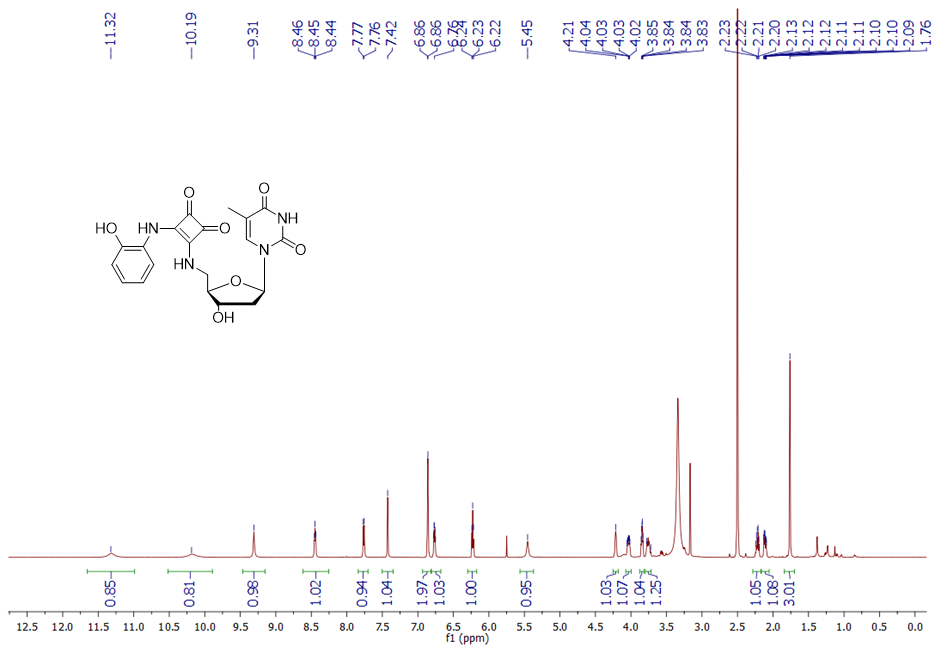


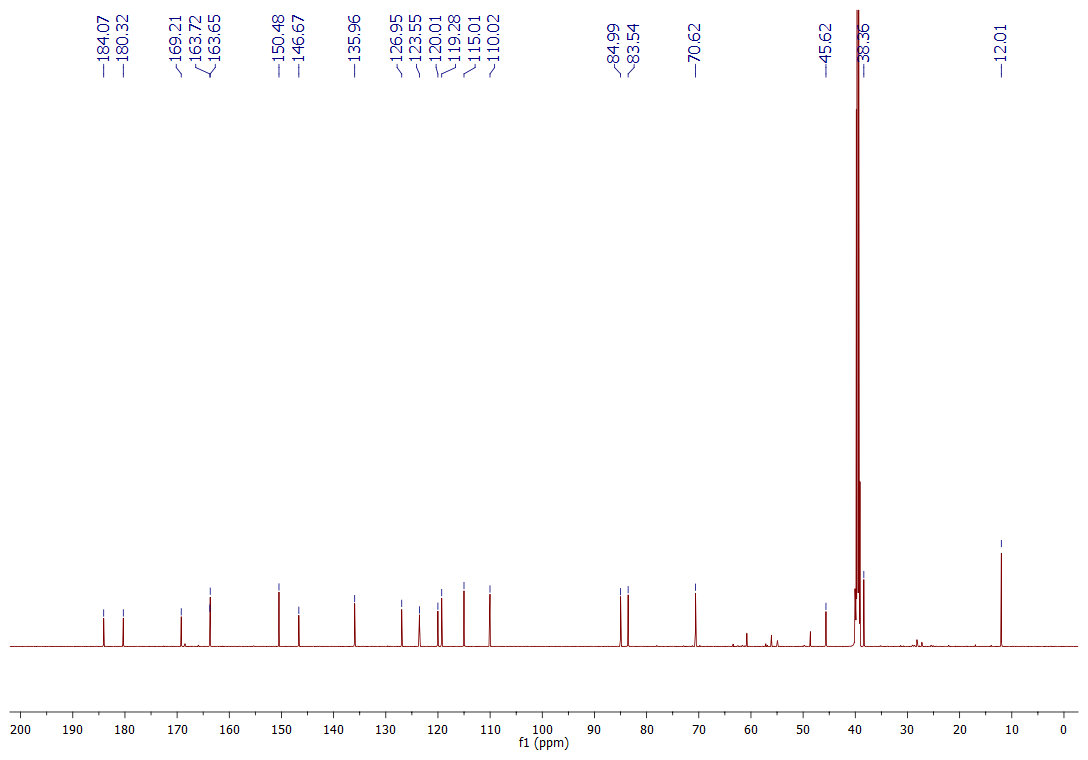


^1^H (400 MHz, D_2_O) and ^13^C (100 MHz, D_2_O) NMR spectra of **4**
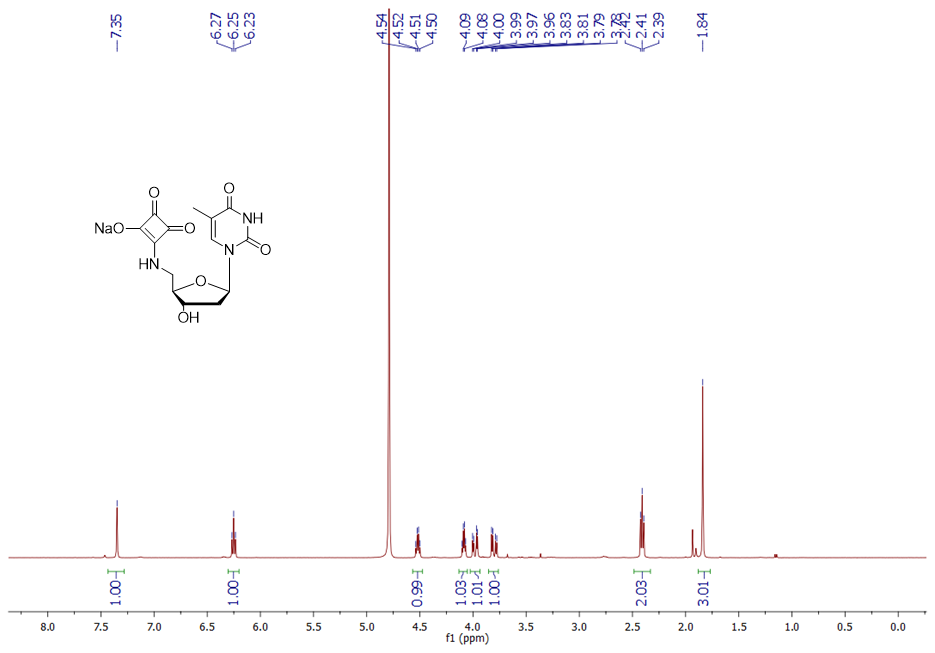


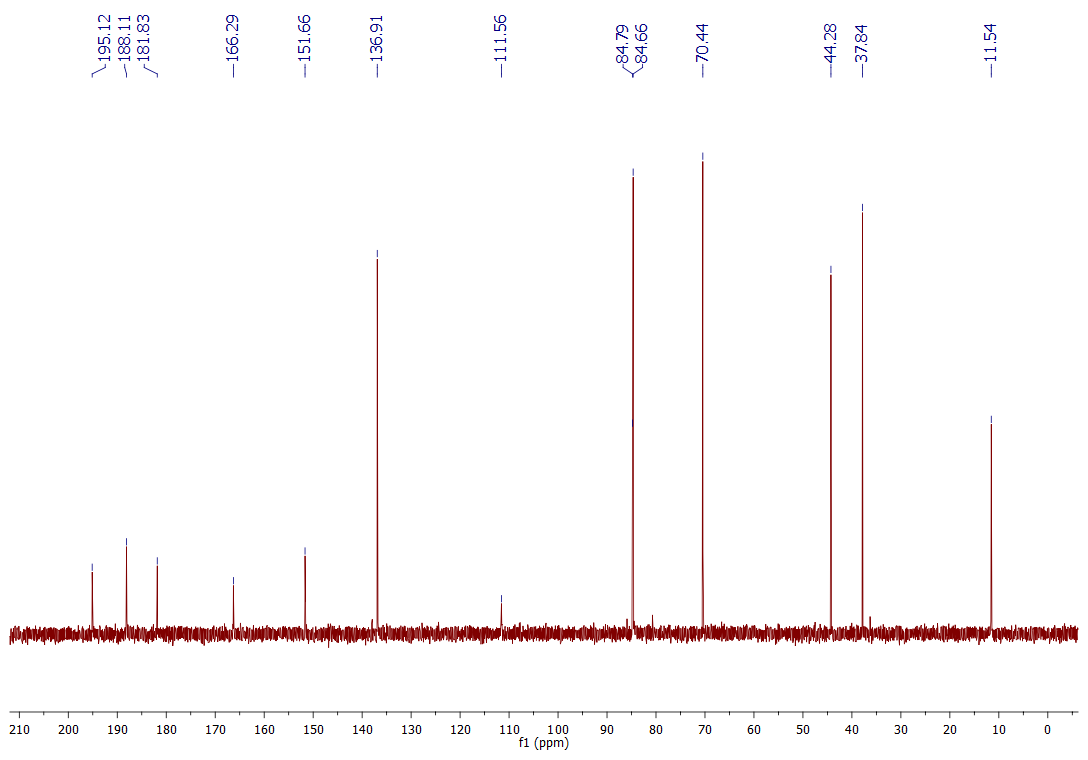


^1^H (400 MHz, DMSO-d_6_) and ^13^C (100 MHz, DMSO-d_6_) NMR spectra of **6**


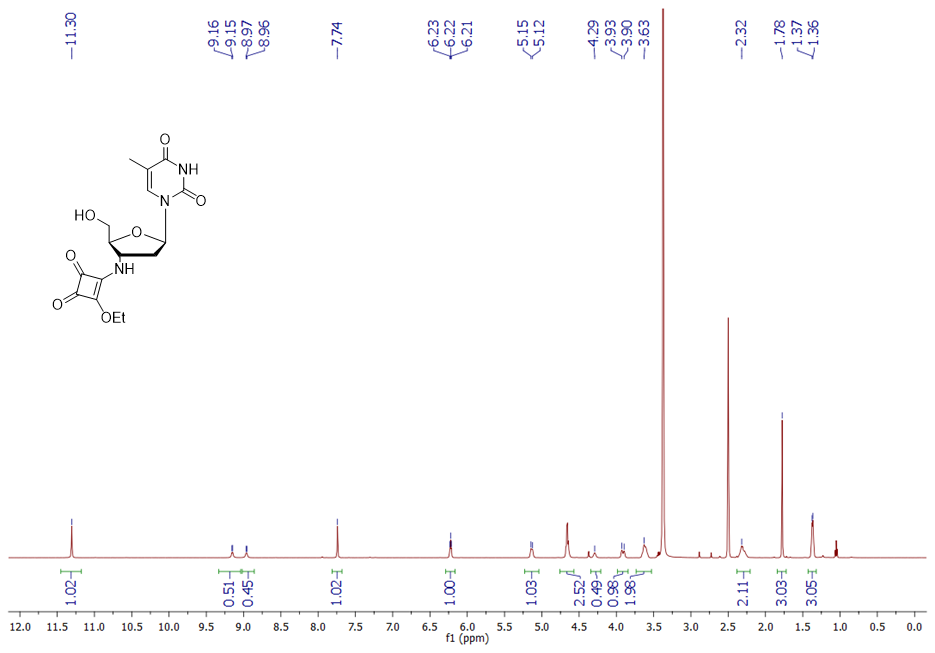


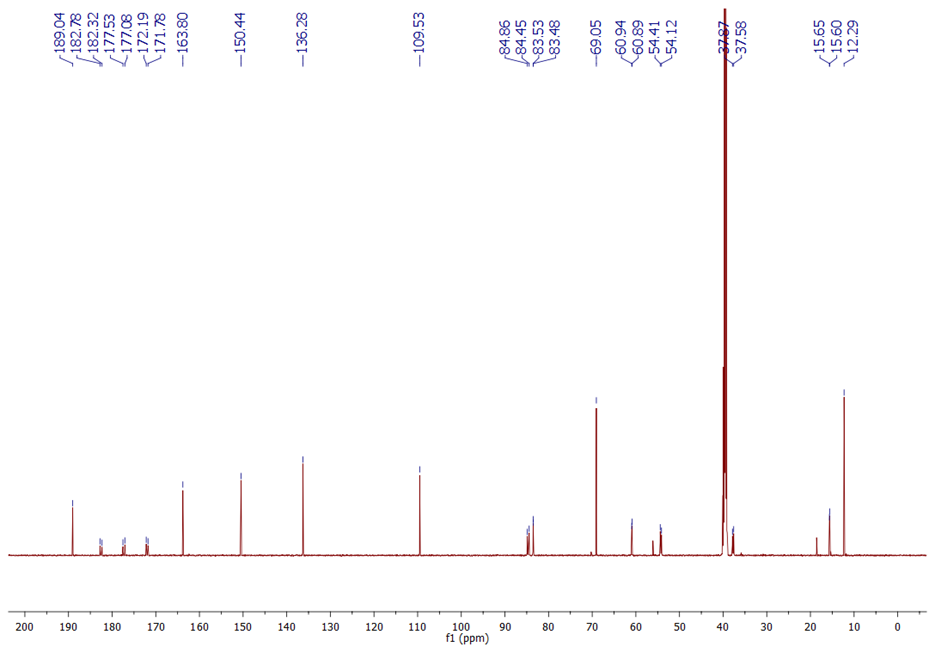


^1^H (400 MHz, DMSO-d_6_) and ^13^C (100 MHz, DMSO-d_6_) NMR spectra of **7**


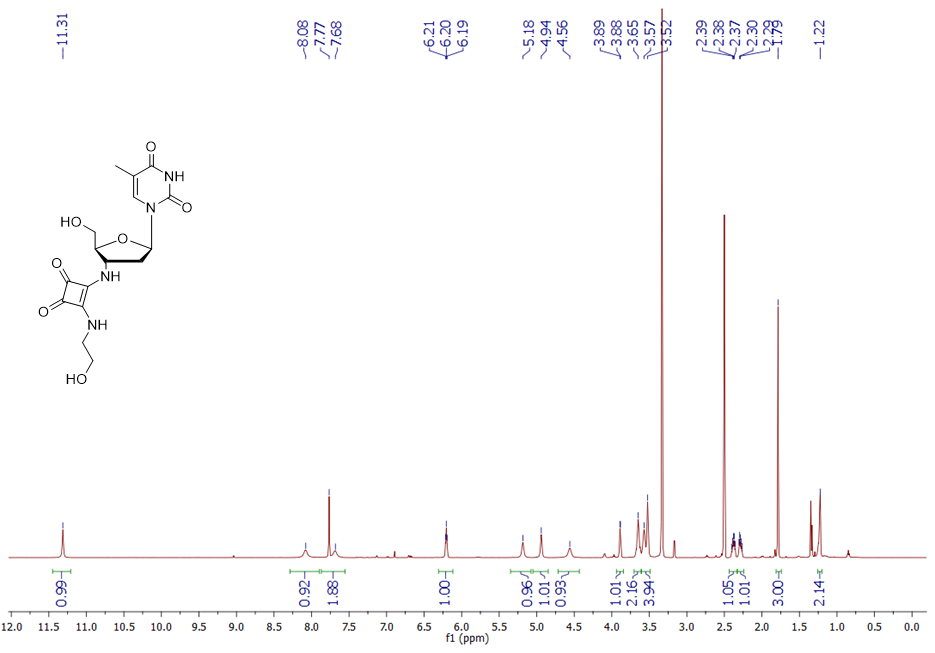


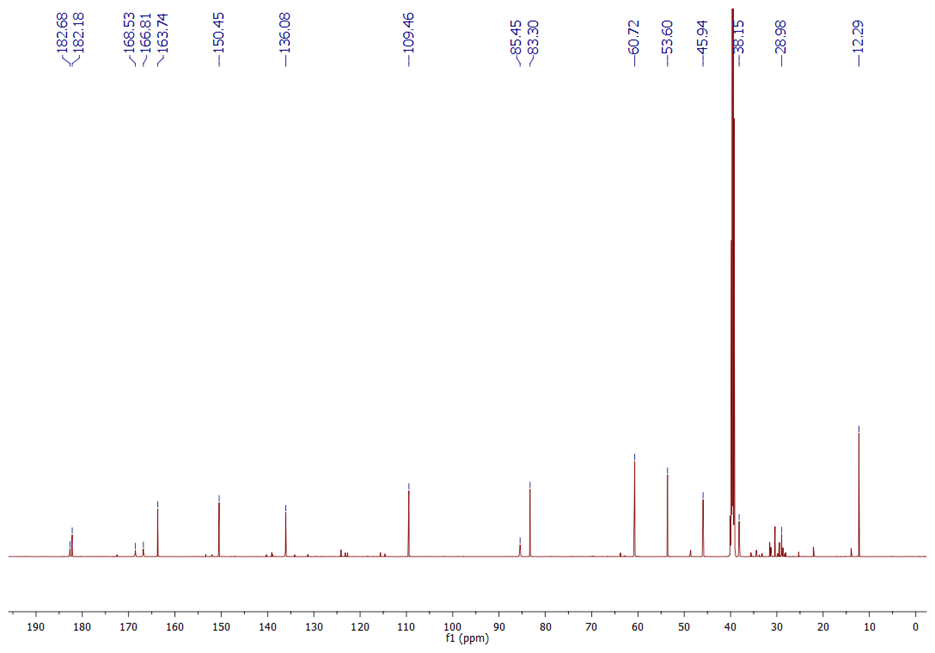


^1^H (400 MHz, D_2_O) and ^13^C (100 MHz, D_2_O) NMR spectra of **8**


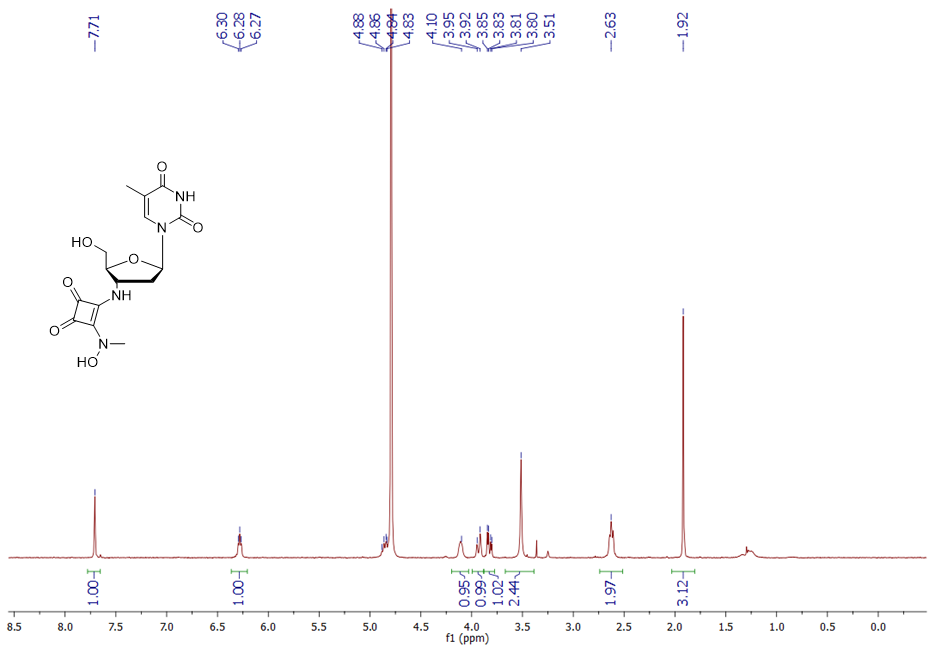


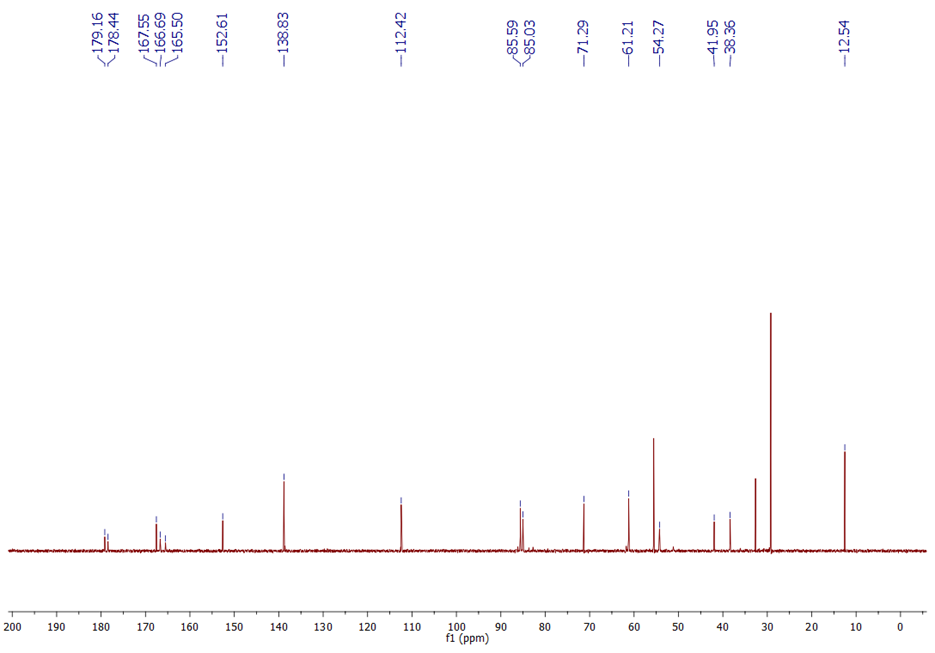


^1^H (400 MHz, D_2_O) and ^13^C (100 MHz, D_2_O) NMR spectra of **9**


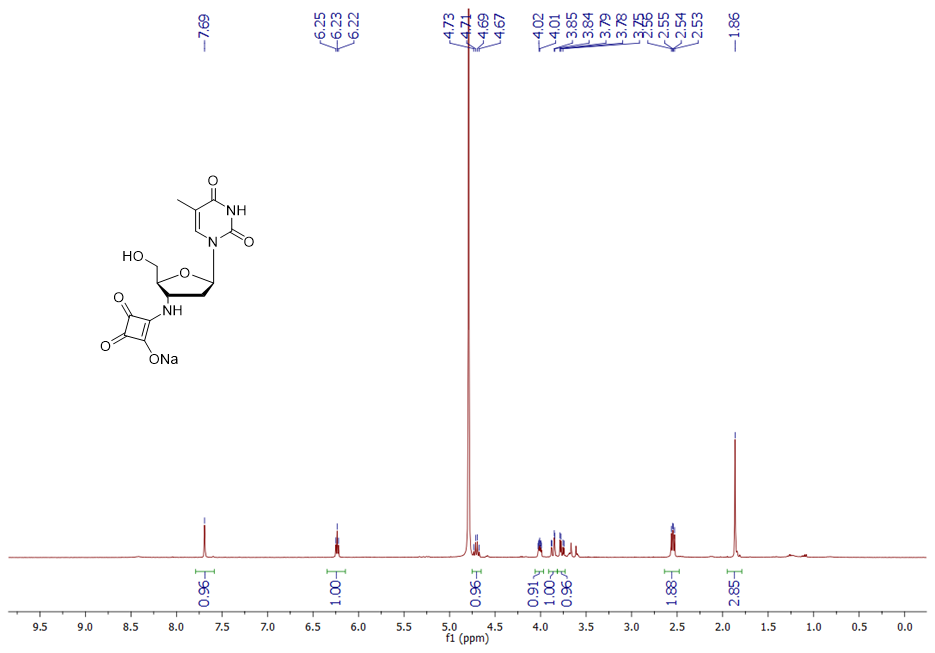


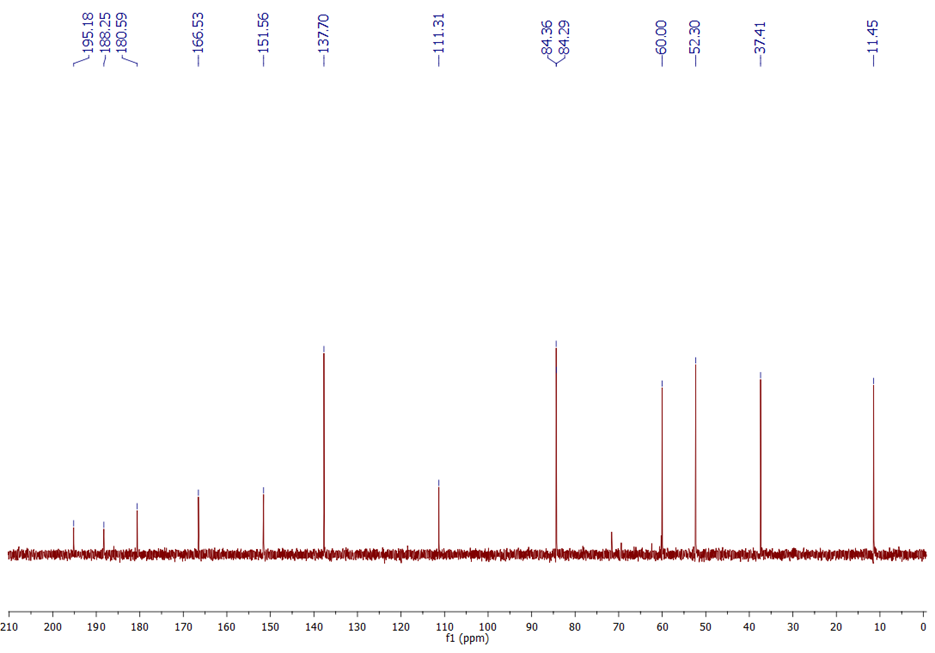


^1^H (600 MHz, DMSO-d_6_) and ^13^C (151 MHz, DMSO-d_6_) NMR spectra of **13** and **14**


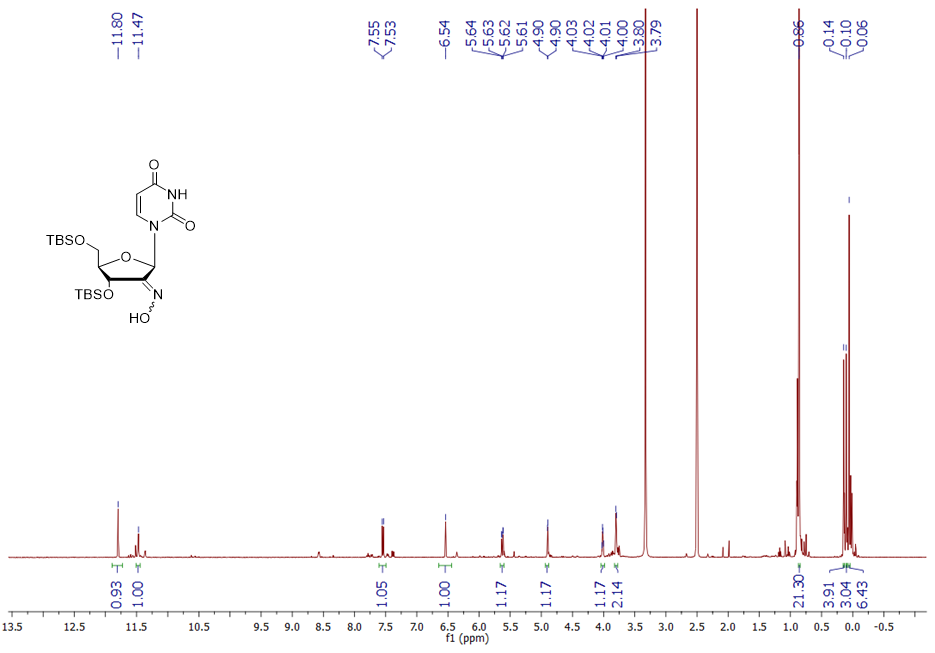


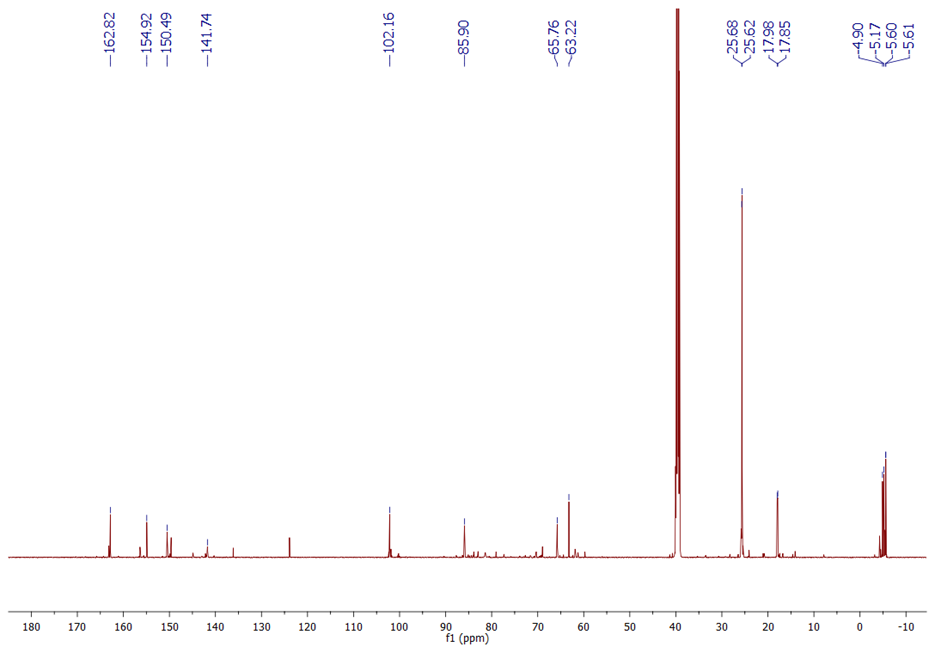


^1^H (600 MHz, DMSO-d_6_) and ^13^C (151 MHz, DMSO-d_6_) NMR spectra of **19**


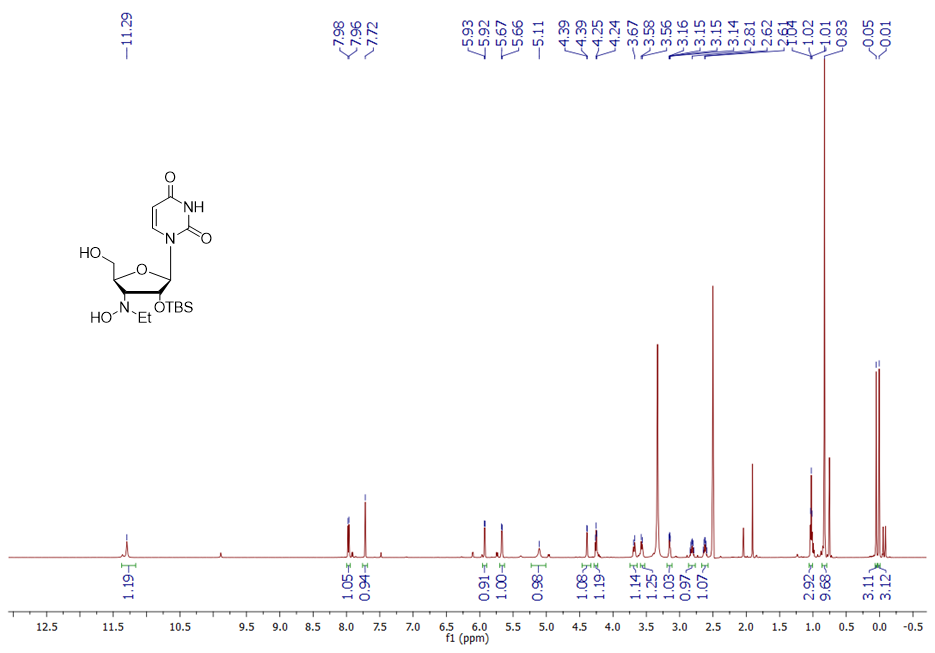


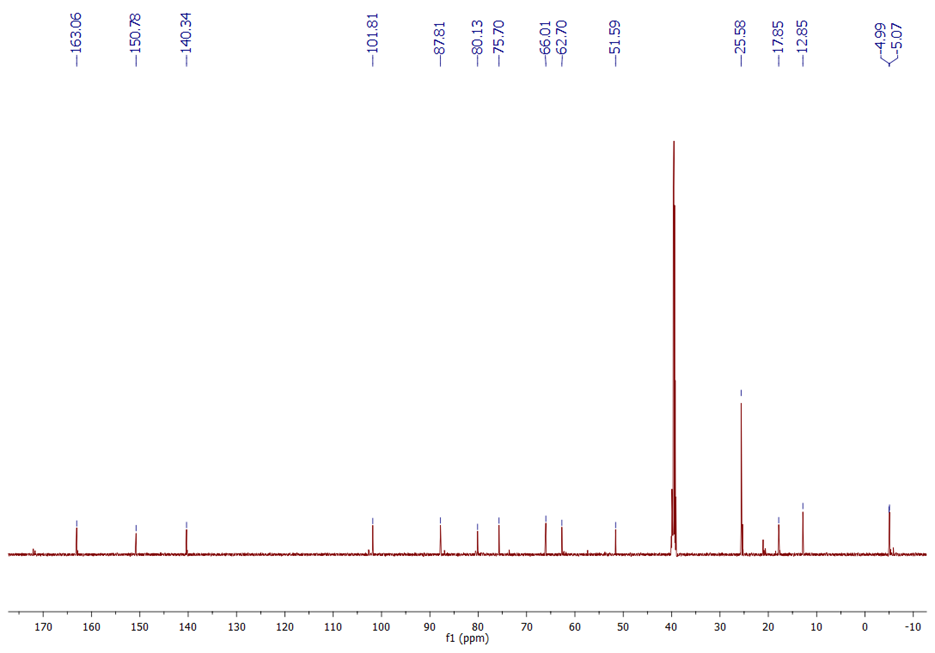


^1^H (600 MHz, DMSO-d_6_) and ^13^C (151 MHz, DMSO-d_6_) NMR spectra of **22**


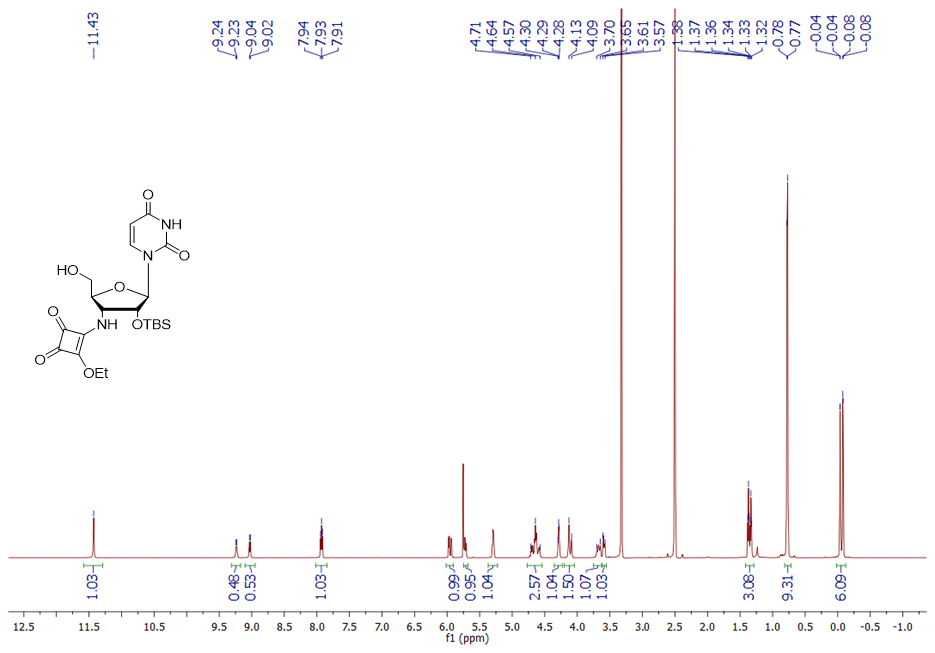


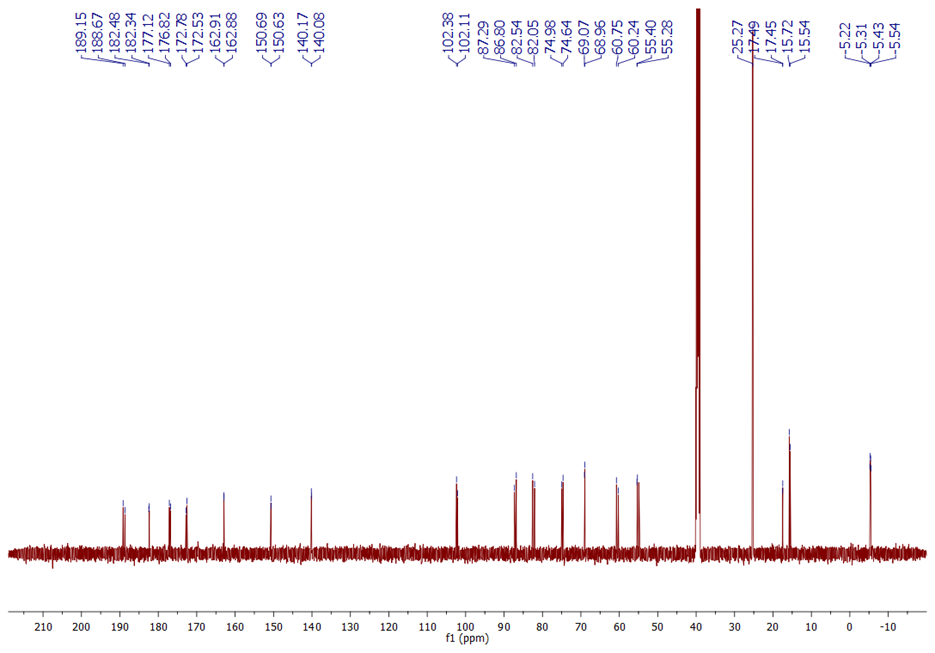


^1^H (600 MHz, acetone-d_6_) and ^13^C (151 MHz, acetone-d_6_) NMR spectra of **23**


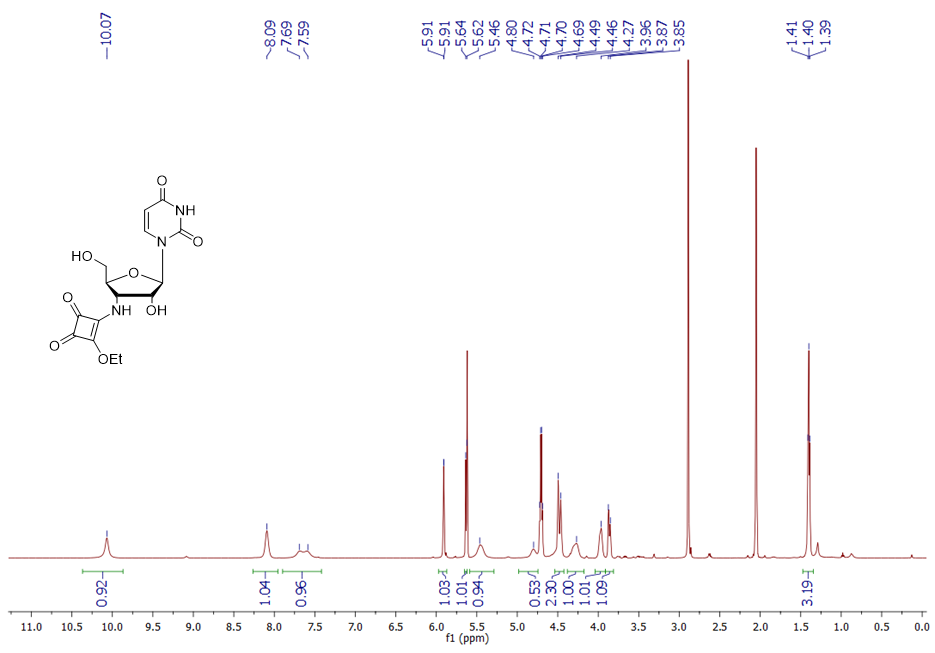


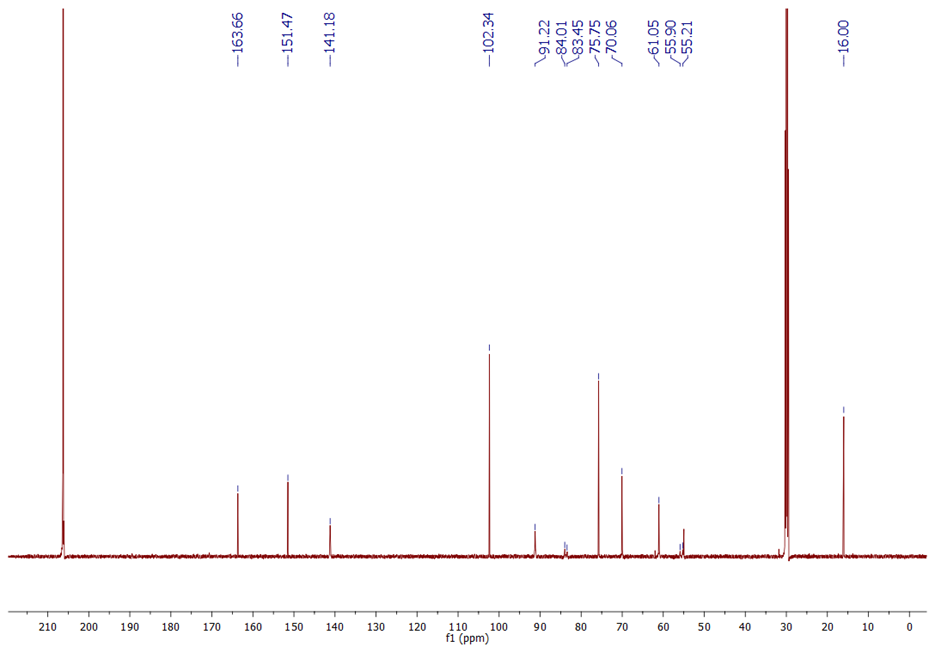


^1^H (600 MHz, DMSO-d_6_) and ^13^C (151 MHz, DMSO-d_6_) NMR spectra of **24**


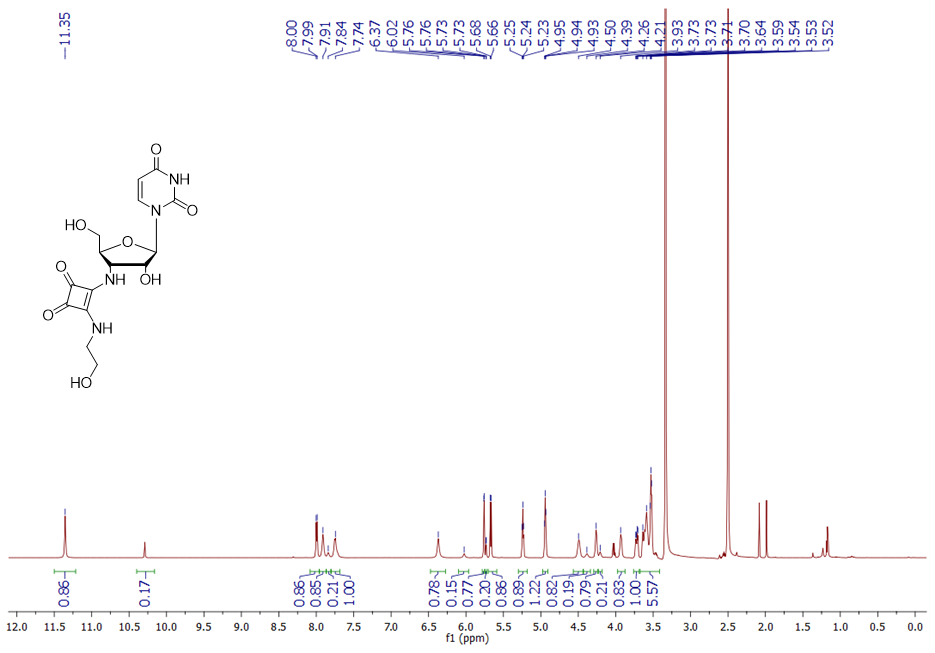


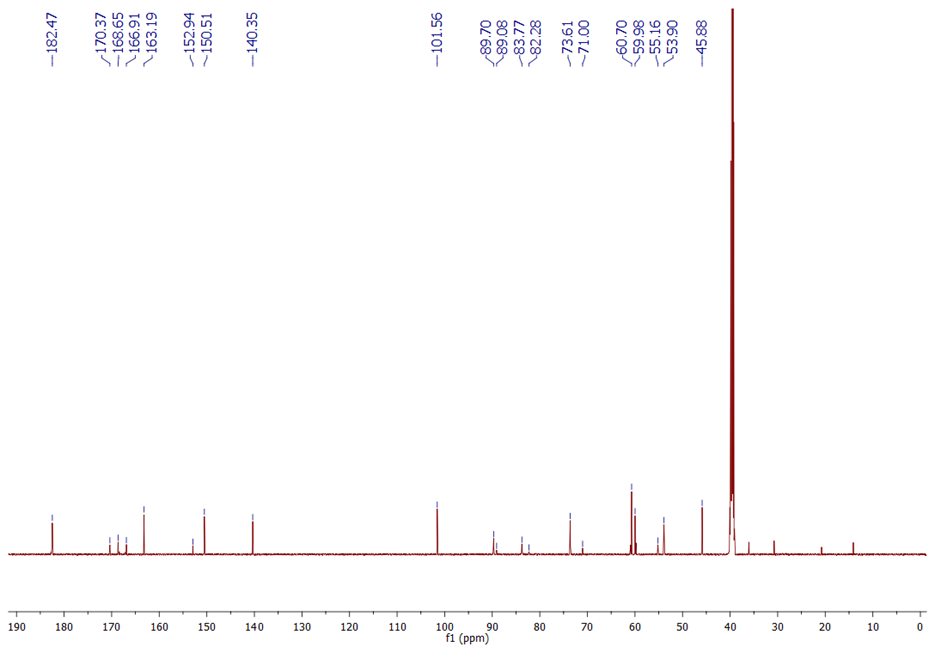


^1^H (600 MHz, DMSO-d_6_) and ^13^C (151 MHz, DMSO-d_6_) NMR spectra of **25**


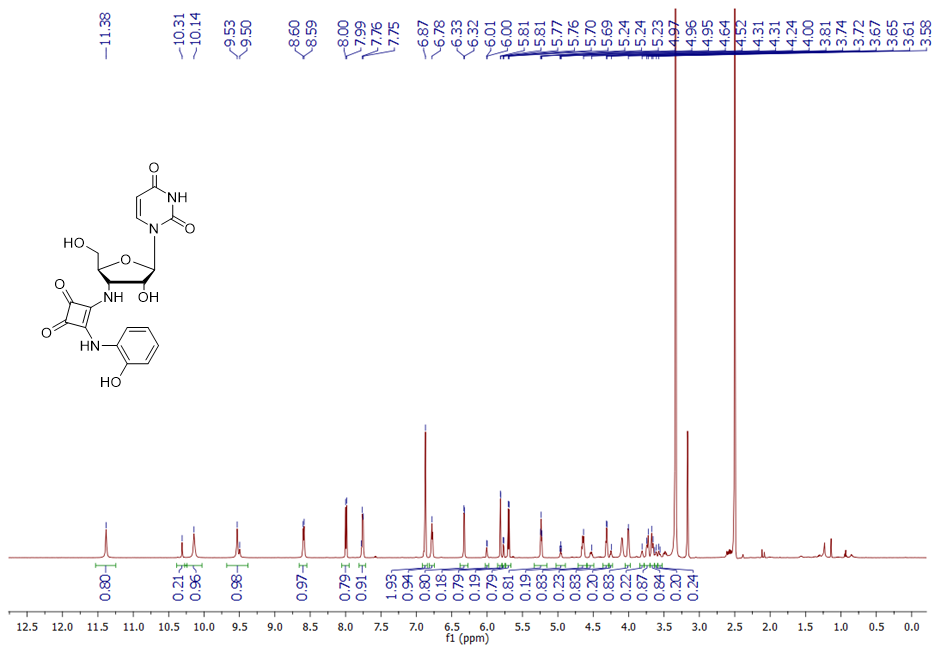


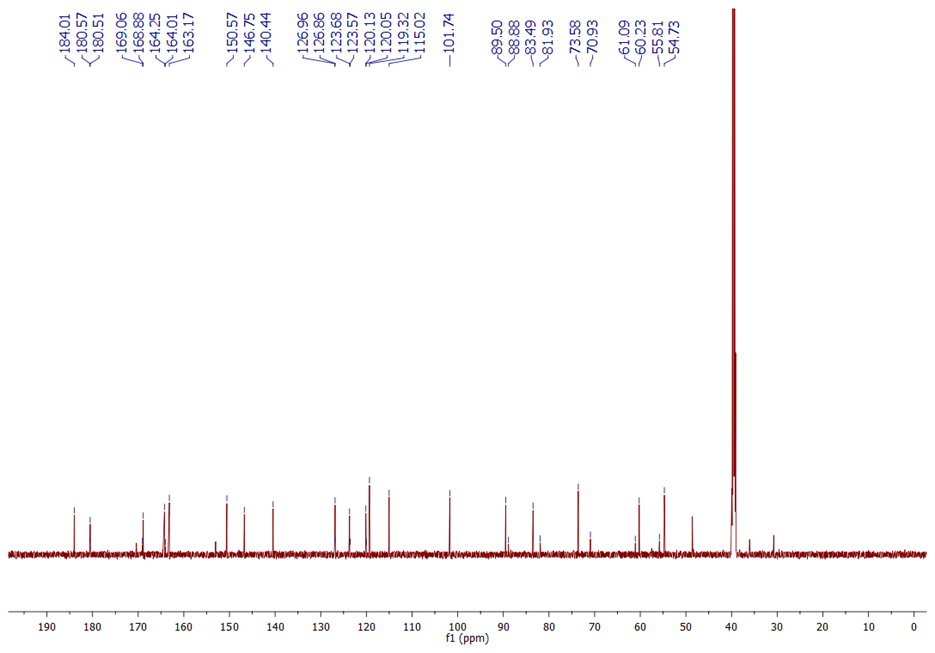


^1^H (600 MHz, DMSO-d_6_) and ^13^C (151 MHz, DMSO-d_6_) NMR spectra of **26**


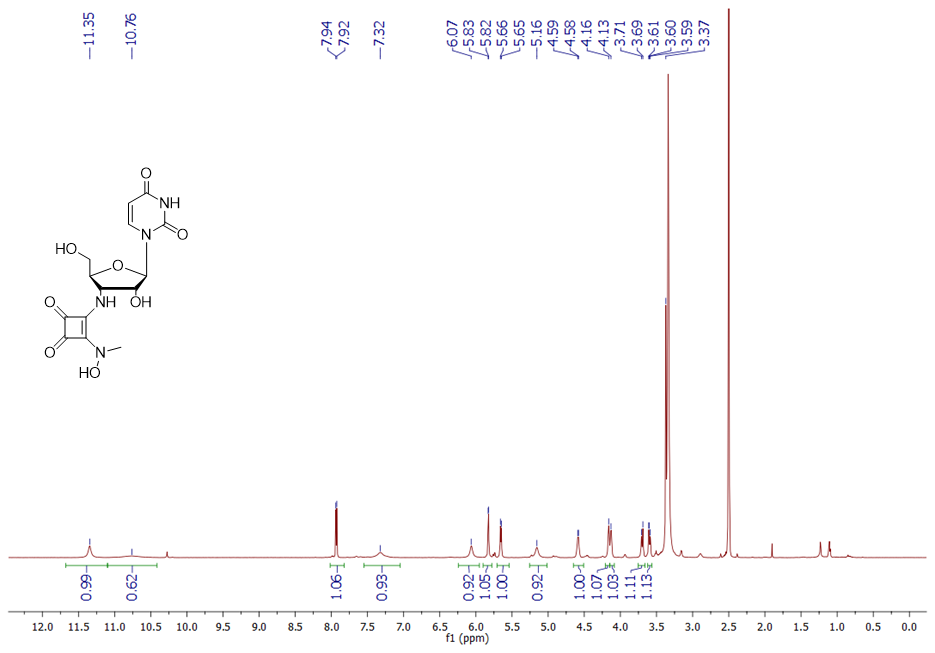


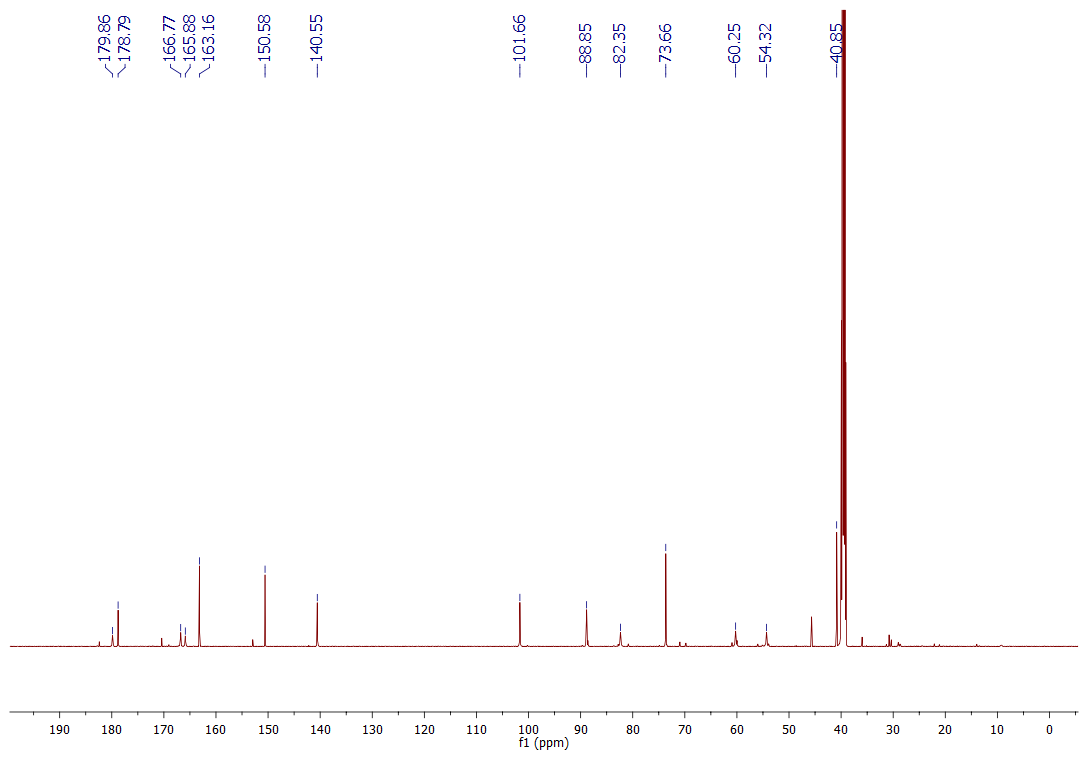


^1^H (600 MHz, D_2_O) and ^13^C (151 MHz, D_2_O) NMR spectra of **27**


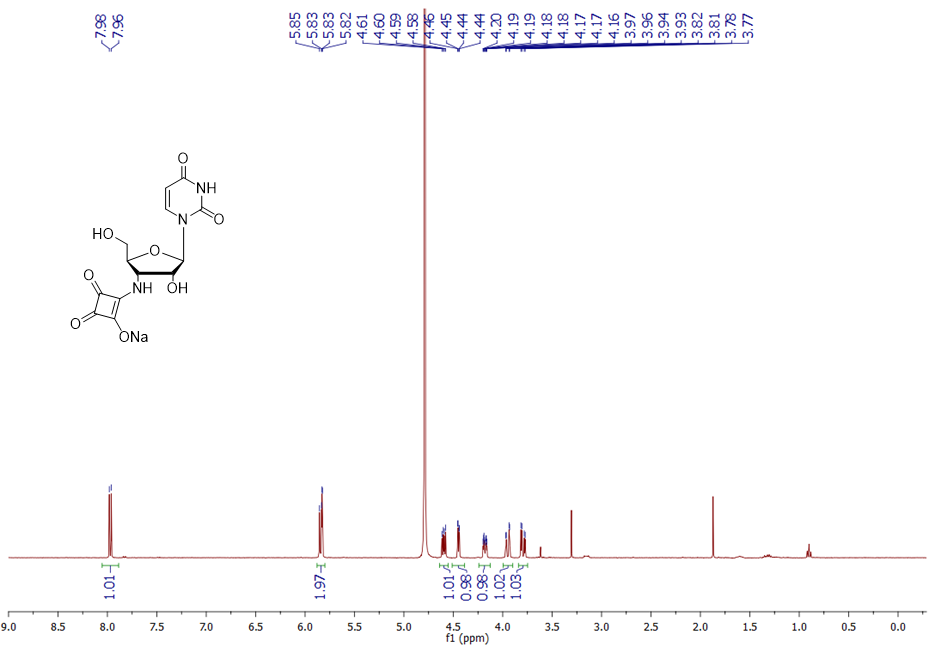


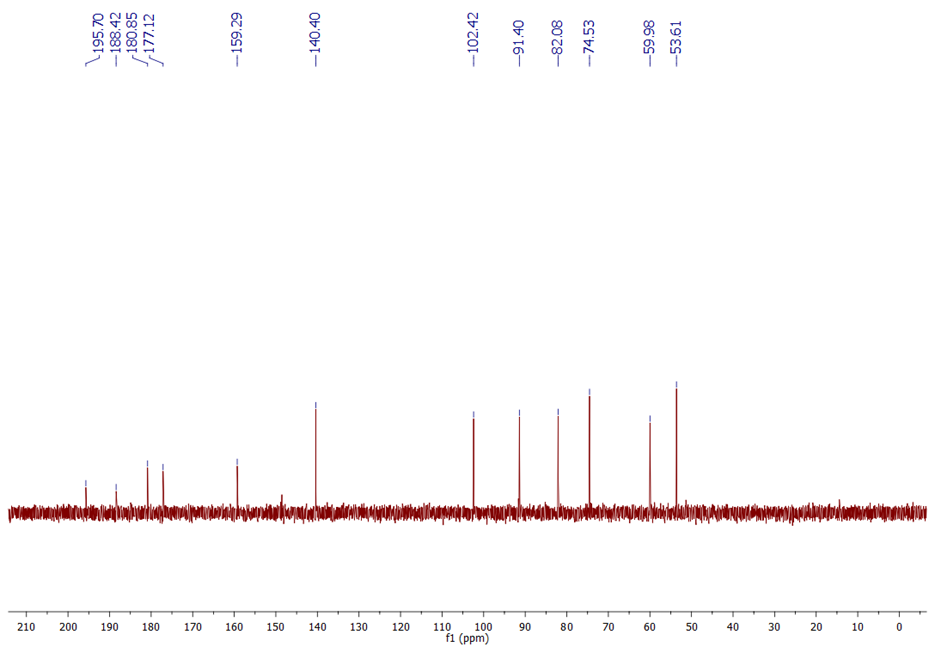


^1^H (400 MHz, DMSO-d_6_) and ^13^C (100 MHz, DMSO-d_6_) NMR spectra of **30**


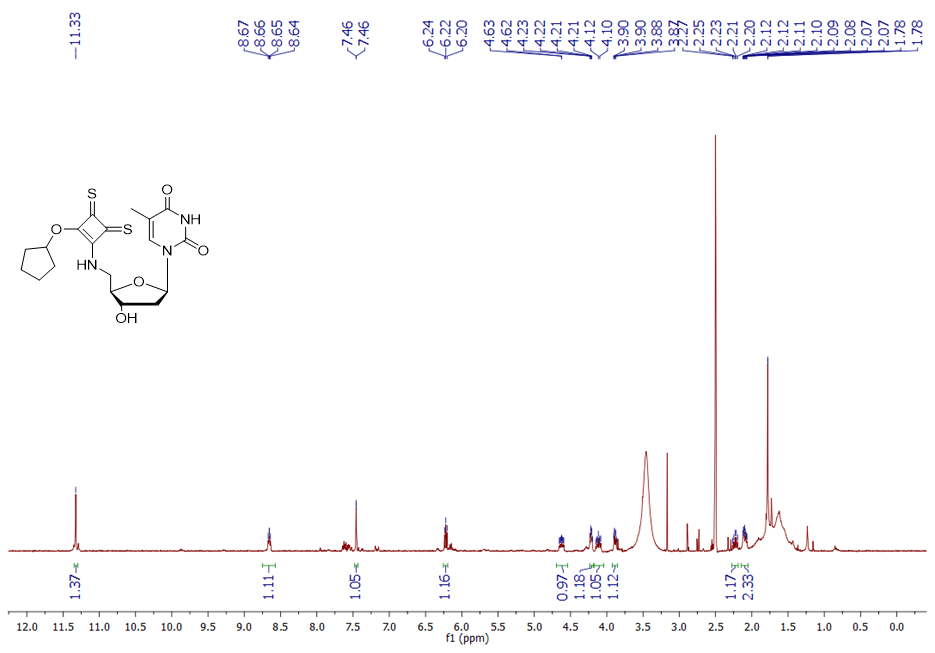

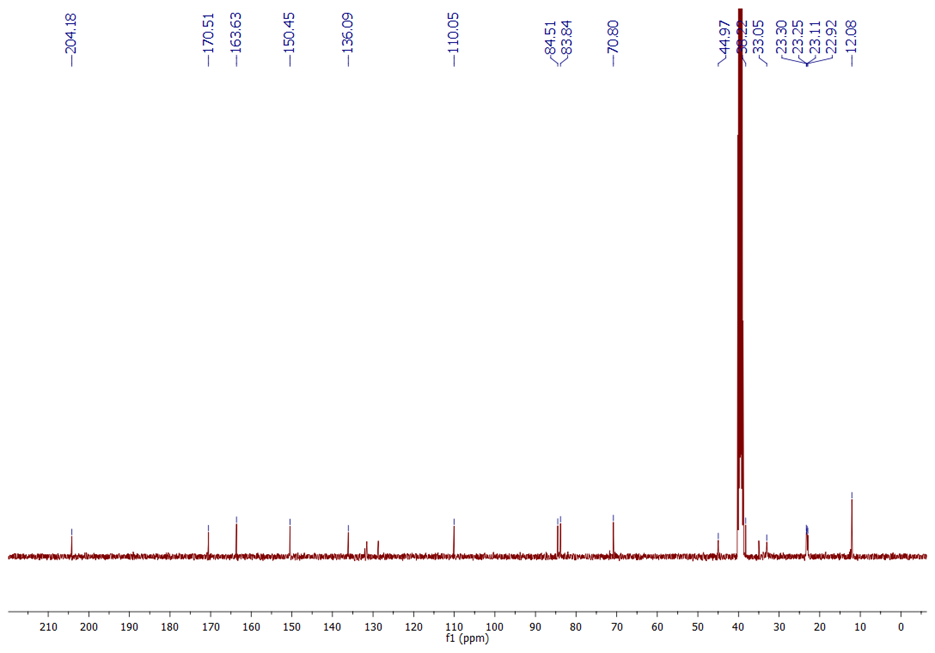


^1^H (400 MHz, DMSO-d_6_) and ^13^C (100 MHz, DMSO-d_6_) NMR spectra of **31**


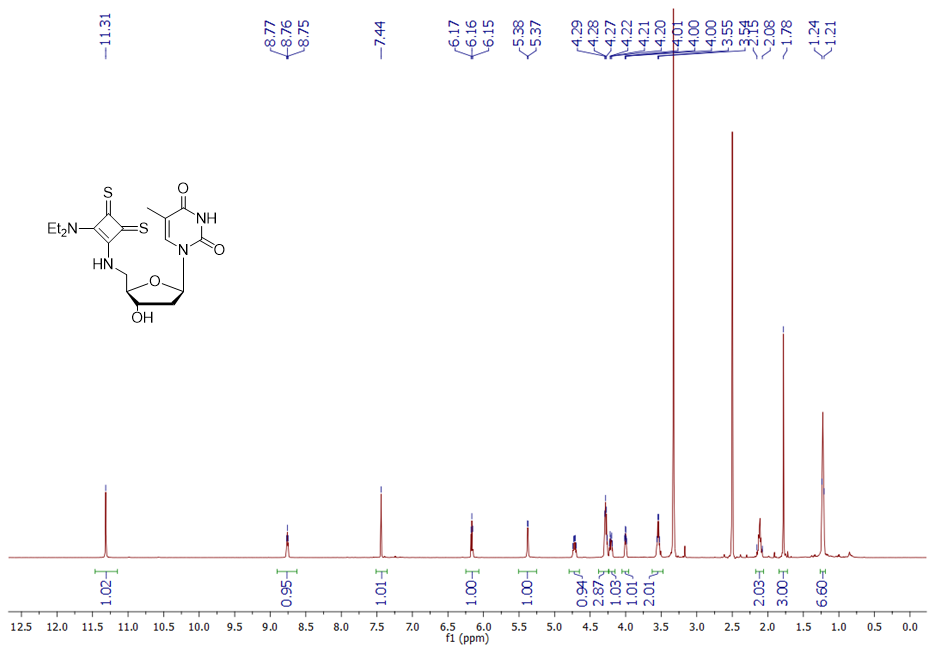


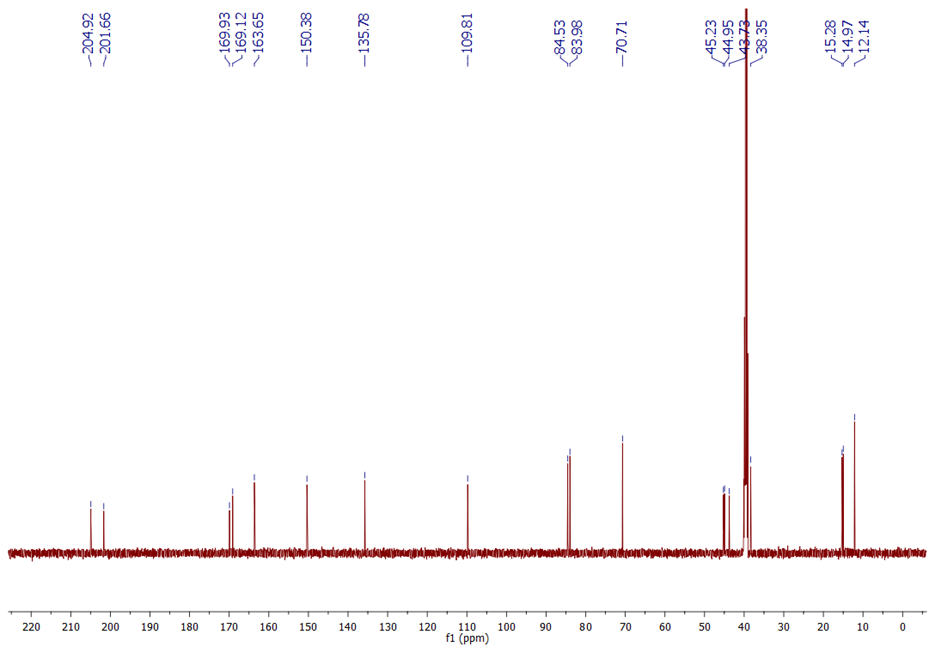


^1^H (600 MHz, DMSO-d_6_) and ^13^C (151 MHz, DMSO-d_6_) NMR spectra of **35**


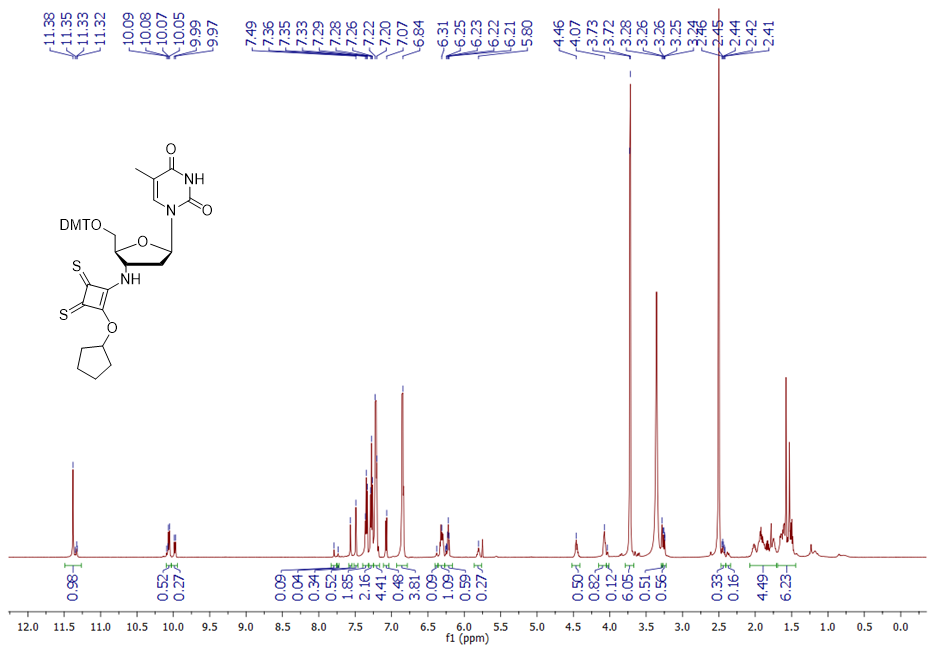

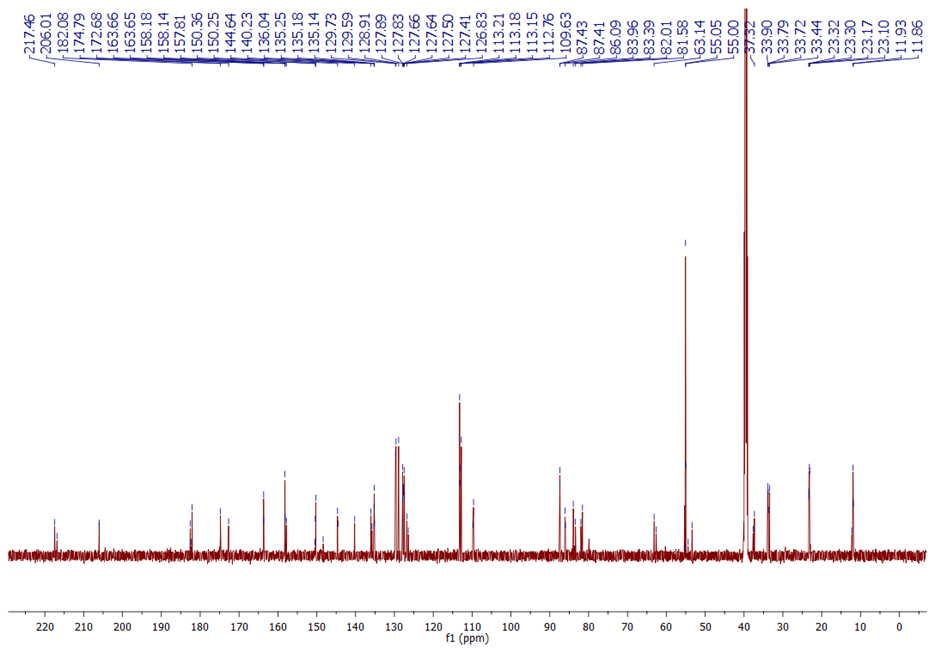


^1^H (600 MHz, acetone-d_6_) and ^13^C (151 MHz, acetone-d_6_) NMR spectra of **36**


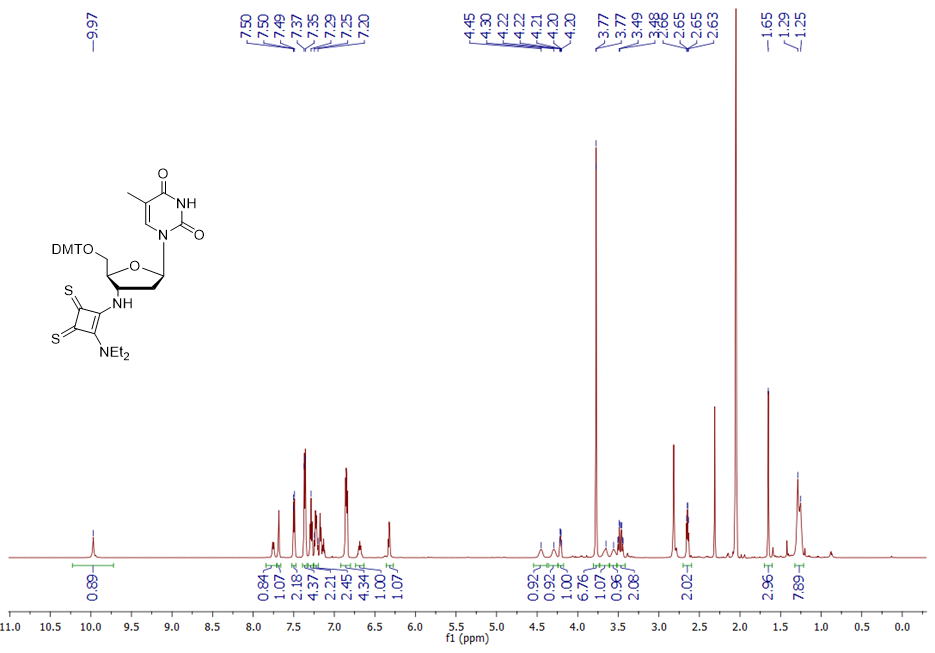


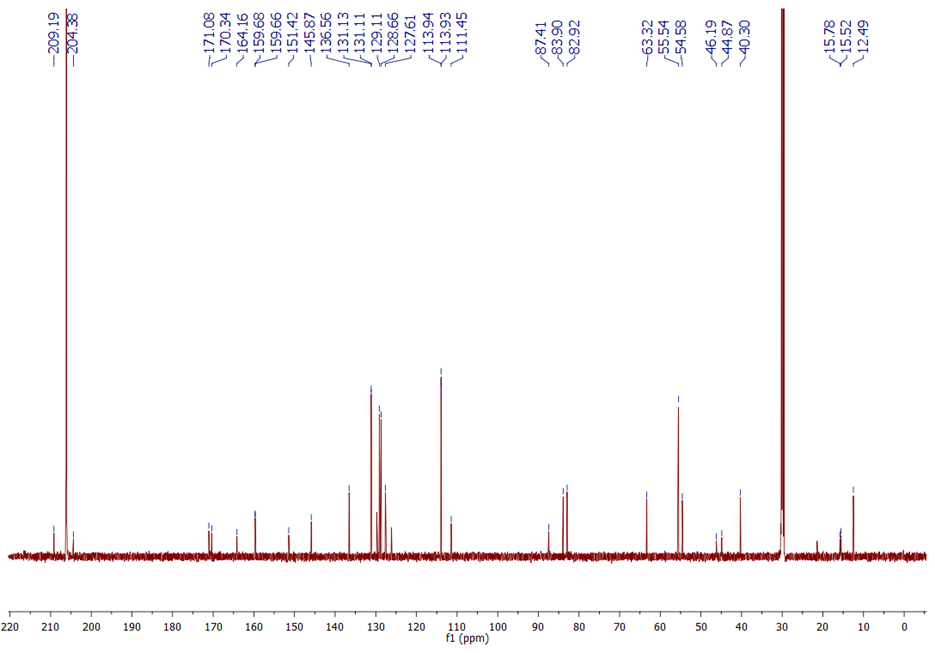


^1^H (600 MHz, acetone-d_6_) and ^13^C (151 MHz, acetone-d_6_) NMR spectra of **37**


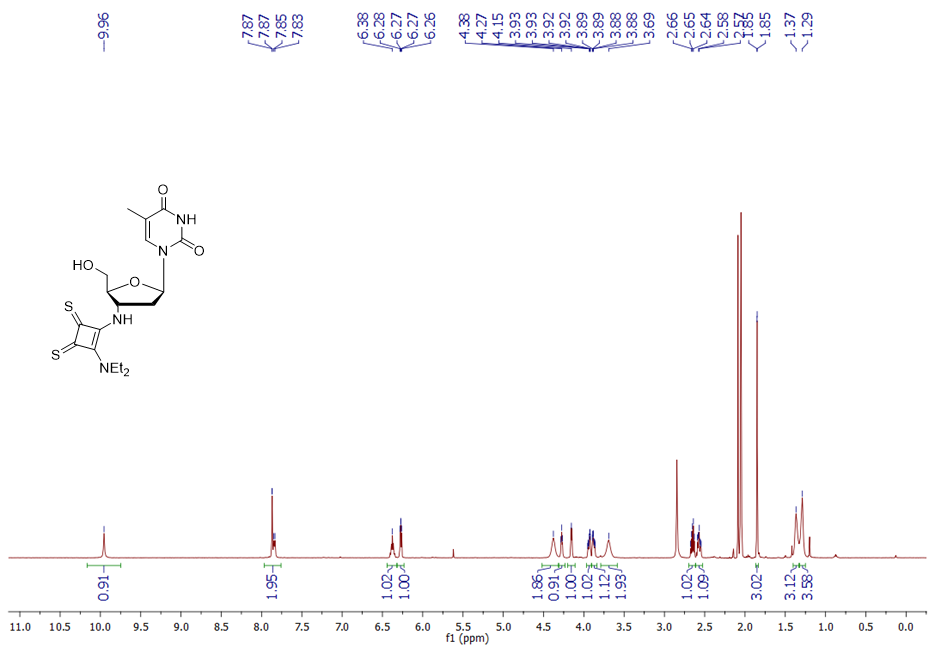


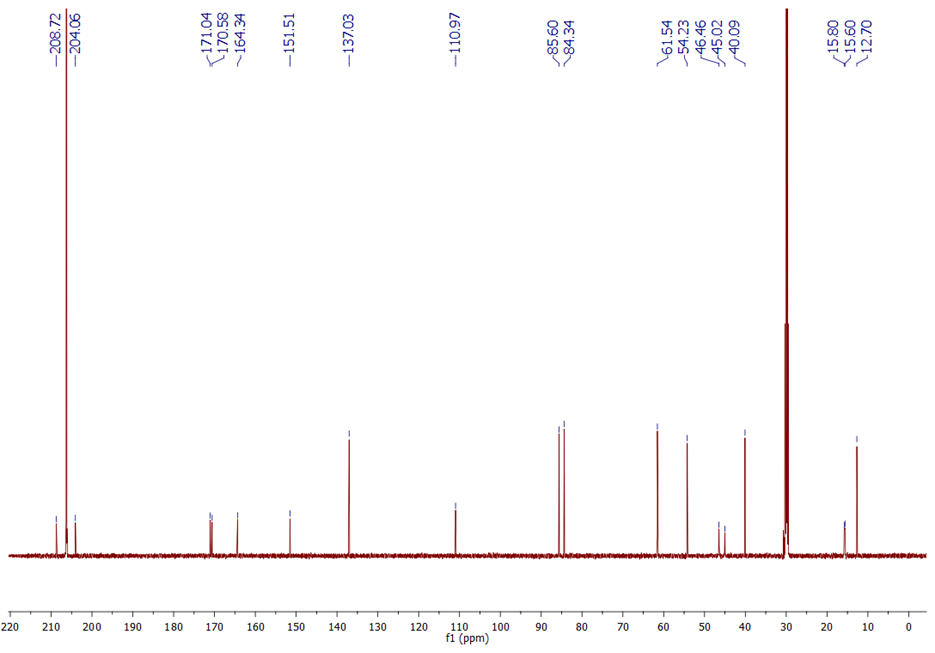


^1^H (600 MHz, DMSO-d_6_) and ^13^C (151 MHz, DMSO-d_6_) NMR spectra of **38**

^
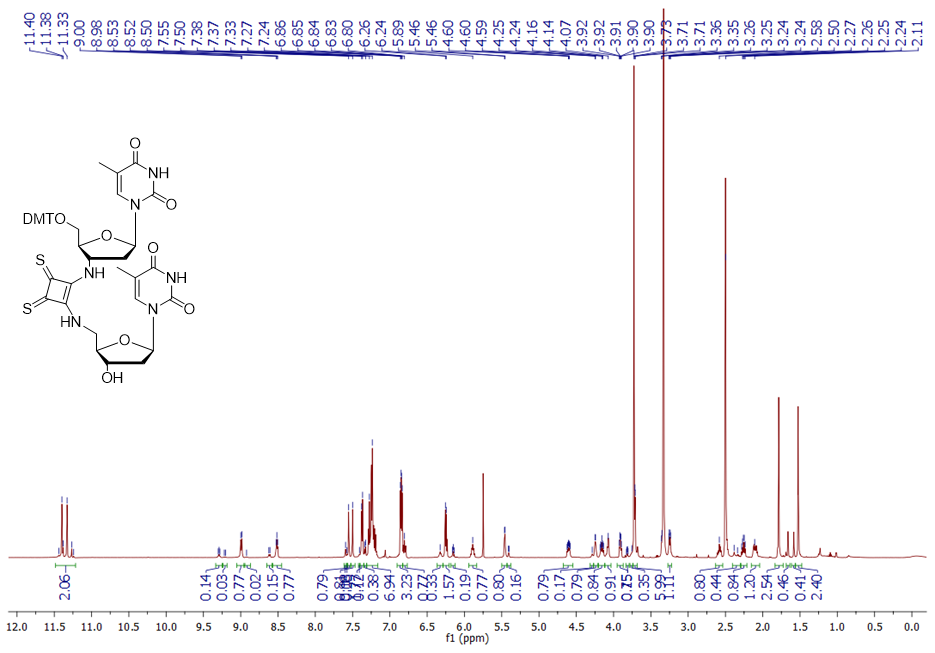

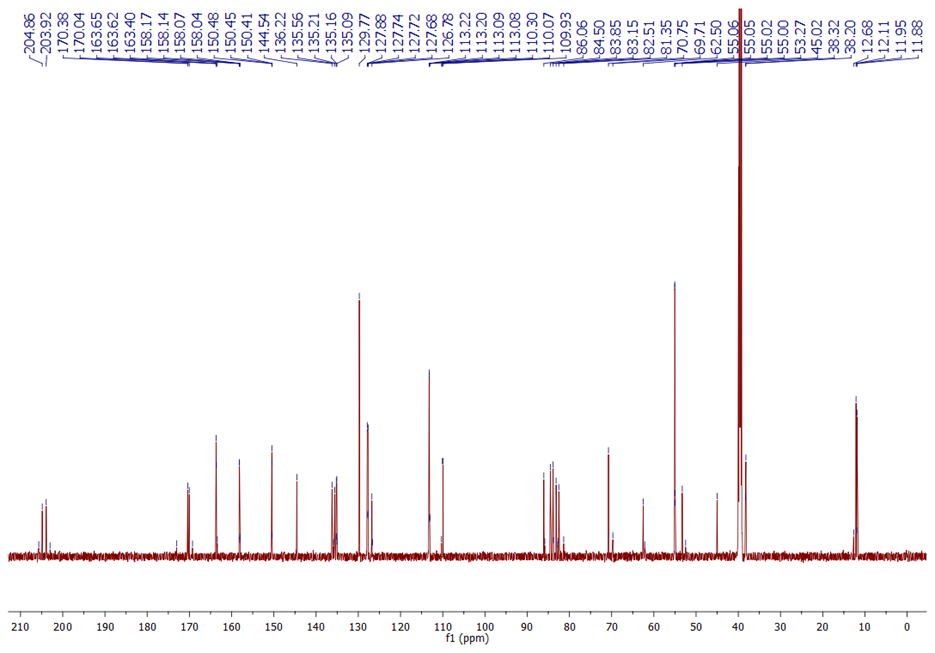
^

^1^H (600 MHz, DMSO-d_6_) and ^13^C (151 MHz, DMSO-d_6_) NMR spectra of **39**


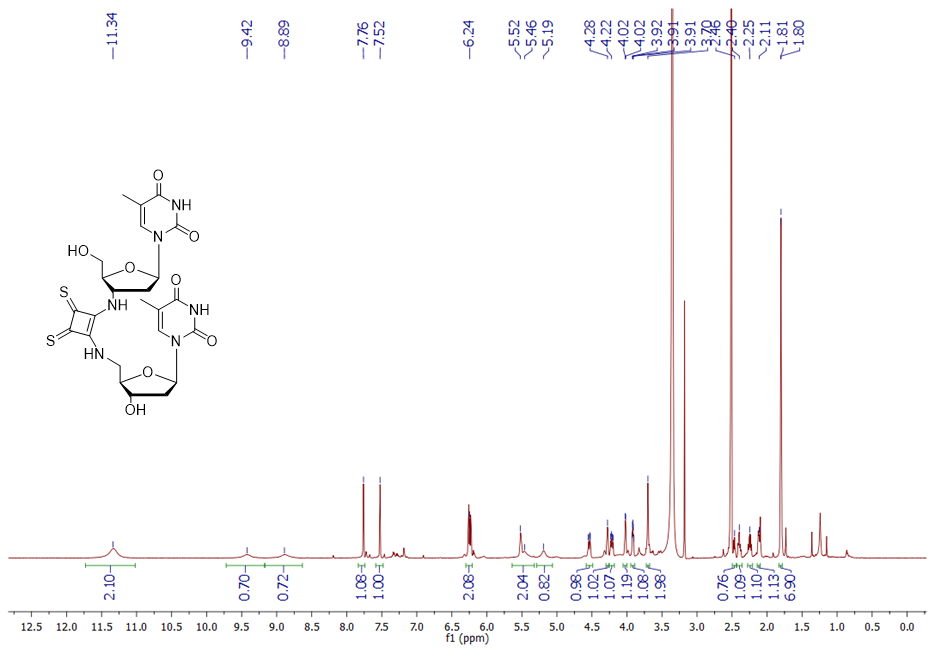


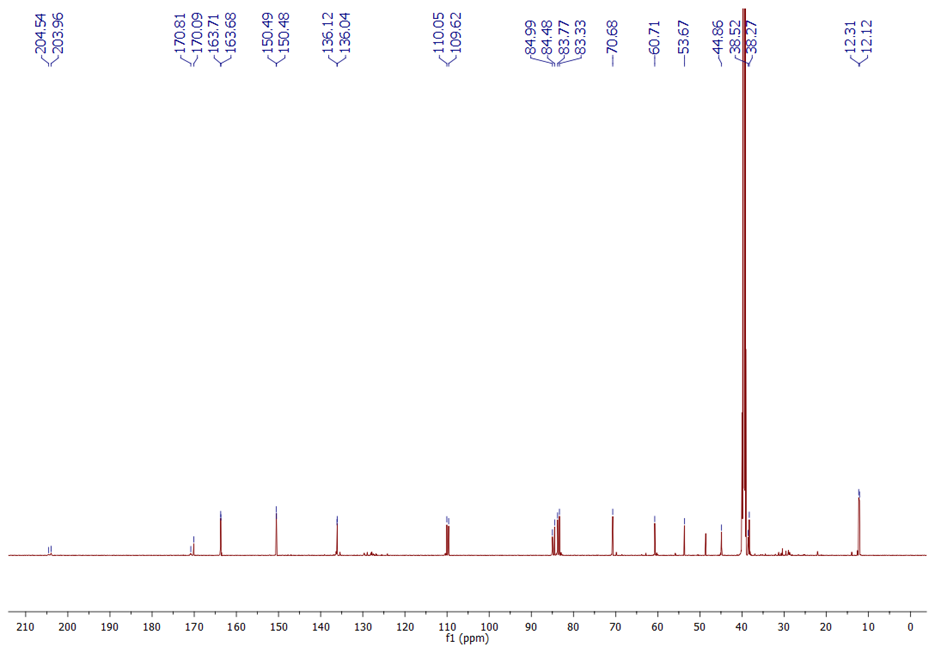


# References

1. Seio, K., Miyashita, T., Sato, K. & Sekine, M. Synthesis and properties of new nucleotide analogues possessing squaramide moieties as new phosphate isosters. *European J. Org. Chem.* **2005**, 5163–5170 (2005).

2. Bege, M., Bereczki, I., Herczeg, M., Kicsák, M., Eszenyi, D., Herczegh, P. & Borbás, A. A low-temperature, photoinduced thiol–ene click reaction: a mild and efficient method for the synthesis of sugar-modified nucleosides. *Org. Biomol. Chem.* **15**, 9226–9233 (2017).

3. Samano, V. & Robins, M. J. Nucleic acid related compounds. 60. Mild periodinane oxidation of protected nucleosides to give 2’- and 3’-ketonucleosides. The first isolation of a purine 2’-deoxy-3’-ketonucleoside derivative. *J. Org. Chem.* **55**, 5186–5188 (1990).

4. Kojima, N., Szabo, I. E. & Bruice, T. C. Synthesis of ribonucleic guanidine: replacement of the negative phosphodiester linkages of RNA with positive guanidinium linkages. *Tetrahedron* **58**, 867–879 (2002).

5. Gogoi, K., Gunjal, A. D., Phalgune, U. D. & Kumar, V. A. Synthesis and RNA binding selectivity of oligonucleotides modified with five-atom thioacetamido nucleic acid backbone structures. *Org. Lett.* **9**, 2697–2700 (2007).

6. Chen, J.-K., Schultz, R. G., Lioyd, D. H. & Gryaznov, S. M. Synthesis of oligodeoxyribonucleotide N3′→P5′ phosphoramidates. *Nucleic Acids Res.* **23**, 2661–2668 (1995).

7. Lee, S. Y., Brem, J., Pettinati, I., Claridge, T. D. W., Gileadi, O., Schofield, C. J. & McHugh, P. J. Cephalosporins inhibit human metallo β-lactamase fold DNA repair nucleases SNM1A and SNM1B/apollo. *Chem. Commun.* **52**, 6727–6730 (2016).

8. Doherty, W., Dürr, E. M., Baddock, H. T., Lee, S. Y., McHugh, P. J., Brown, T., Senge, M. O., Scanlan, E. M. & McGouran, J. F. A hydroxamic-acid-containing nucleoside inhibits DNA repair nuclease SNM1A. *Org. Biomol. Chem.* **17**, 8094–8105 (2019).

9. Schmidt, D. & Lynch, J. Millipore Corporation Application Note. Lit. No. AN1728EN00 (2003).
